# In Silico Study About Substituent Effects, Electronic Properties, and the Biological Potential of 1,3-Butadiene Analogues

**DOI:** 10.3390/ijms26188983

**Published:** 2025-09-15

**Authors:** Karolina Kula, Emilia Kuś

**Affiliations:** Cracow University of Technology, Faculty of Chemical Engineering and Technology, Warszawska 24, 31-155 Cracow, Poland

**Keywords:** conjugated diene, substituent effect, electronic reactivity indices, physicochemical properties, in silico, MEDT, ADME, PASS

## Abstract

1,3-Butadiene and its analogues constitute an important raw material in the petrochemical industry. What is more, due to their specific structure, these compounds are attractive components in the synthesis of heterocyclic compounds. Modification of the 1,3-butadiene structure allows obtaining compounds characterized by different reactivity and possessing various biological properties. In order to thoroughly investigate this phenomenon, an analysis of 20 compounds, including 1,3-butadiene and its analogues, was carried out. For this purpose, a study based on MEDT, ADME, and PASS was performed. In this research, changes in electronic properties and basic physicochemical parameters under the presence of various substituents at various positions in the structure of 1,3-butadiene were studied. At the end, the influence of modifications on biological activities for the tested compounds was evaluated. Based on the presented results, it was found that substituent modifications cause significant changes in both electronic structures and in physicochemical properties of all the compounds. This fact is probably caused by the small size of the considered compounds. On the other hand, the main preferences for the most important active sites in the tested molecules remain the same due to the presence of a strongly conjugated system of double bonds.

## 1. Introduction

**Figure 1:**
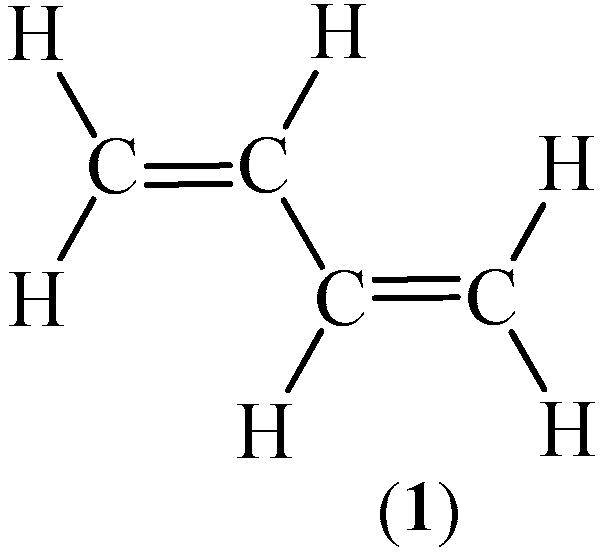
The structure of 1,3-butadiene (**1**).

1,3-Butadiene (**1**) is the simplest member of the conjugated diene group, commonly used in organic synthesis (Figure 1) [1]. The compound (**1**) is obtained mainly through the process of extraction from the C4 fraction produced in the steam cracking process [2]. It is estimated that over 95% of this hydrocarbon (**1**) is obtained using this method [3]. Other technologies rely on catalytic dehydrogenation of butane [4,5] or butane fraction [6,7,8].

The global production of this hydrocarbon (**1**) is constantly growing. The world production of 1,3-butadiene (**1**) in 2024 was estimated to be almost 22 million tons. Until 2028 the increase in the production to ca. 27 million tons is projected [9]. The constantly growing demand for this petrochemical raw material (**1**) results from its wide range of industrial applications [10].

The presence of the conjugated double bond system in the structure makes 1,3-butadiene (**1**) highly susceptible to polymerization and autoxidation reactions. Due to this fact, the main industrial application direction of this hydrocarbon (**1**) includes rubber production such as styrene butadiene rubber (SBR), acrylonitrile butadiene rubber (NBR), and acrylonitrile–butadiene–styrene (ABS), which are feedstocks used to produce tyres and plastic materials [11,12]. Moreover, 1,3-butadiene (**1**) is a key intermediate for chemical production such as adiponitrile, chloroprene, 4-vinylcyclohexene, and others [2,13].

Due to the unique structure of 1,3-butadiene (**1**) as well as the fact that it can be relatively easily condensed to a liquid state [14], the compound (**1**) is widely used in the synthesis of heterocyclic systems. For this purpose, the most commonly used protocol is cycloaddition reactions [15,16]. In particular, the 6π-electron processes are the most important. These reactions are common methods to synthesize carbo- and heterocyclic compounds [17,18]. What is more, the mentioned processes are useful to the preparation of bis-ring systems [19], which are widespread in optoelectronics [20]. In addition, several studies have demonstrated the potential of 1,3-butadiene (**1**) in medicinal chemistry. In particular, this hydrocarbon (**1**) has been effectively employed in the synthesis of active pharmaceutical ingredients (APIs) and a wide range of biological compounds [21].

**Figure 2:**
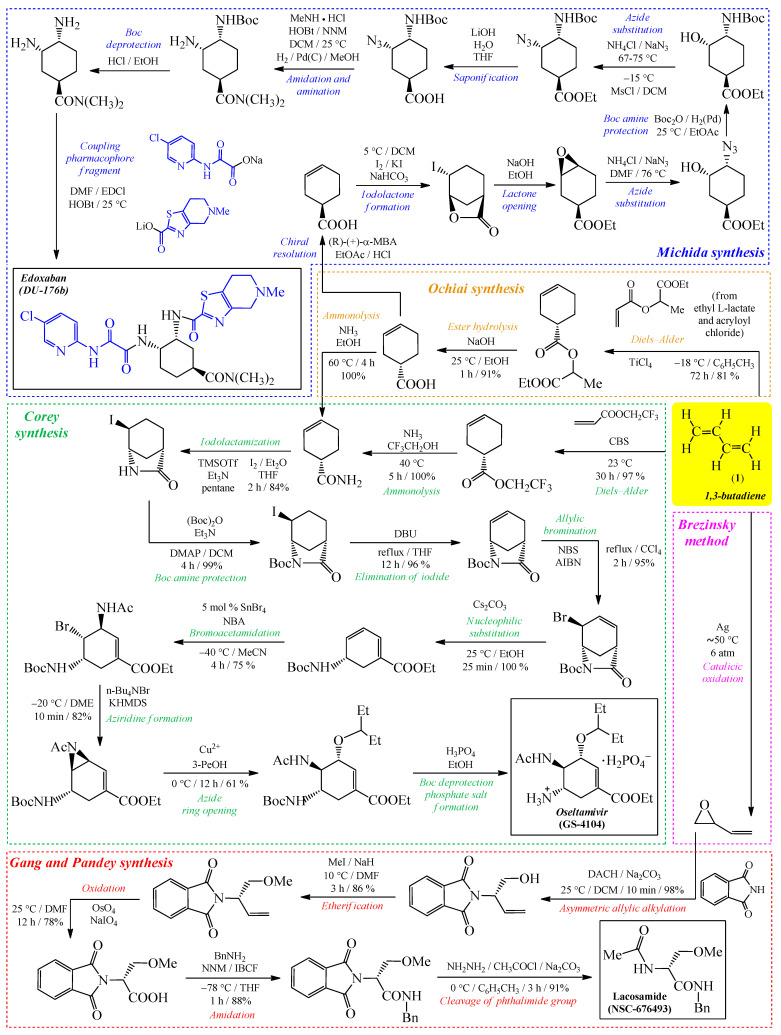
The most significant application routes of 1,3-butadiene (**1**) for the synthesis of APIs and other biologically active compounds.

One of the best-known examples of technology of API production based on 1,3-butadiene (**1**) was reported by *Corey* et al. [22] in 2006. In this study, the authors carried out an eleven-step synthetic sequence, beginning with the Diels–Alder reaction of 1,3-butadiene (**1**) with 2,2,2-trifluoroethyl acrylate, which ultimately afforded *Oseltamivir* (GS-4104) in its phosphate form (Figure 2). In general, *Oseltamivir* is a well-known antiviral drug clinically approved for both the treatment and prophylaxis of influenza A and B infections. Its mechanism of action relies on the inhibition of viral neuraminidase, thereby preventing the release of progeny virions from infected host cells [23]. The authors emphasize that their strategy relies on simple and inexpensive commercially available starting materials, thereby reducing production costs and enhancing the scalability of the process. The overall yield of the described process is estimated to be approximately 30%, which is considered remarkably high given the complexity of the synthetic sequence [22].

In 2022, *Ochiai* et al. [24] reported a reaction that can serve as an efficient alternative for the synthesis of 3-cyclohexene-1-carboxamide. The authors first treated ethyl L-lactate with acryloyl chloride, yielding ethyl lactylacrylate. This intermediate was subsequently subjected to the Diels–Alder reaction with 1,3-butadiene (**1**), ultimately affording 3-cyclohexene-1-carboxylic acid (Figure 2). This compound serves as a precursor not only for the synthesis of *Oseltamivir*, but it can also be employed in the preparation of *Edoxaban* (DU-176b)—a clinically used anticoagulant for the treatment of venous thrombosis [25].

Another example of API synthesis based on a 1,3-butadiene core was reported in 2015 by *Garg* and *Pandey* [26]. The authors described the use of the oxidized form of this compound (**1**) [27], together with phthalimide, for the synthesis of the antiepileptic drug *Lacosamide* (NSC-676493) (Figure 2). They emphasized that the synthetic method presented in their article allows the production of the pure enantiomer with high selectivity, under relatively simple reaction conditions, and in high yield.

All of the presented approaches not only underscore the synthetic utility of 1,3-butadiene (**1**) as a versatile building block but also demonstrate how fundamental pericyclic reactions can be harnessed in the design of efficient and economically viable routes towards clinically relevant pharmaceuticals.

In addition to the fact that 1,3-butadiene (**1**) itself is an important and valuable compound for the chemical industry, especially in the processes of polymerization, autoxidation, and the synthesis of heterocyclic compounds, its analogues are also widely applied [28,29]. In particular, the modification of the main structure of molecule (**1**) can change properties of the target material as necessary, such as increased flexibility [30] or hardness [31], as well as resistance to environmental factors, including high temperatures [32] and chemical resistance [33,34]. What is more, the introduction of various substituents and/or functional groups into the 1,3-butadiene (**1**) structure increases their attractiveness as well as potential applications as a building block in organic synthesis. Thanks to this, the controlled synthesis of heterocycles with desirable biological properties is possible [35,36,37].

Unfortunately, not all structural aspects of these compounds have been sufficiently explored to date. Therefore, this article aims to fill this gap. In continuation of our research of nitro-functionalized analogues of 1,3-butadiene (**1**), their biological potential, as well as their application in the synthesis of heterocyclic systems [38,39,40], comprehensive computational studies were carried out. For this purpose, a library of 20 compounds, including 1,3-butadiene (**1**) and its mono- and disubstituted analogues (**2a**–**6d**), was examined.

**Figure 3:**
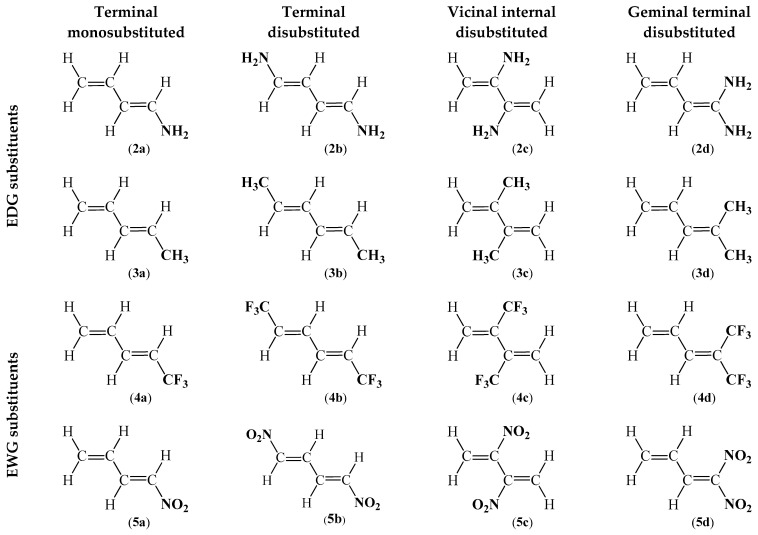
The structure of tested 1,3-butadiene analogues (**2a**–**5d**).

First, it was decided to investigate the effect of a homogeneous substitution by only one type of Electron Donating Group (EDG) or Electron Withdrawing Group (EWG). For this purpose, 16 compounds were evaluated. The structures of the selected compounds differed in two aspects (Figure 3), namely: (I) the type of substituent: -NH_2_ (**2**), -CH_3_ (**3**), -CF_3_ (**4**), and -NO_2_ (**5**); and (II) its position: terminal monosubstituted (**a**), terminal disubstituted (**b**), vicinal internal disubstituted (**c**), and geminal terminal disubstituted (**d**).

**Figure 4:**
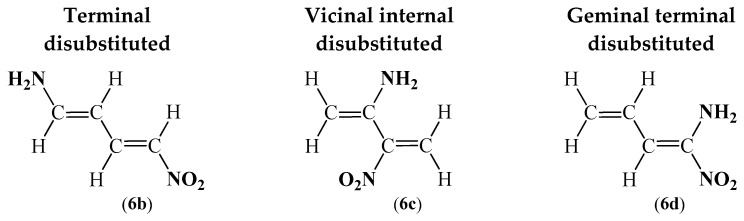
The structure of tested 1,3-butadiene analogues (**6b**–**d**).

In addition, it was decided to investigate how the structure of 1,3-butadiene (**1**) would be influenced by the substitution with an extremely EDG in the form of an amine substituent and an extremely EWG in the form of a nitro group (Figure 4). As is commonly known, compounds containing a conjugated π-bond system in their structure and extremely activating substituents may exhibit the electron *push–pull* effect, which is particularly important for electrical conductivity [41,42]. This approach allows for a comprehensive evaluation of the influence of both the nature and the position of the substituent on the electronic properties of the molecules, the distribution of electron density, the local reactivity profiles, and numerous other critical parameters that dictate both the chemical reactivity of the compounds and their potential biological activity.

It should be underlined that the information presented may prove valuable not only for the future design of similar molecules but also for the application of the studied compounds as reagents with well-defined electronic properties.

## 2. Results and Discussion

The presented research was divided into two main sections. Firstly, a comprehensive analysis of the reactivity of 1,3-butadiene (**1**) and its analogues (**2a**–**6d**) was conducted. For this purpose, selected aspects of Molecular Electron Density Theory (MEDT) [43] were used. What is more, due to the confirmed biological potential of 1,3-butadiene (**1**) and its analogues [44], the in silico study includes evaluation of physicochemical descriptors, pharmacokinetic properties, drug-like nature, and medicinal chemistry friendliness, based on computational ADME (Absorption, Distribution, Metabolism, Excretion) [45] and PASS (Prediction of Activity Spectra for Substances) [46].

### 2.1. Study of Electron Density Distribution Based on MEDT

This computational study has been conducted on 1,3-butadiene (**1**) as the simplest representative of this species and its analogues (**2a**–**6d**) at the ground states. As a result, it was possible to show changes in the structure of the molecules introduced by the presence of various functional groups. Firstly, a topological analysis of Electron Localization Function (ELF) together with Natural Population Analysis (NPA) and Molecular Electrostatic Potential (MEP) mapping has been performed in order to characterize their electronic structures. Next, an analysis of reactivity indices based on Conceptual Density Functional Theory (CDFT) has been carried out.

#### 2.1.1. Analysis of the Electronic Structure Based on ELF, NPA, and MEP

The ELF is a technique that allows prediction of the likelihood of finding an electron in the neighboring space of a reference electron, at a given point and with the same spin [47,48]. As a result, the characterization of electron density distribution in molecules and prediction of their reactivity in reactions are possible [49,50,51,52].

**Figure 5:**
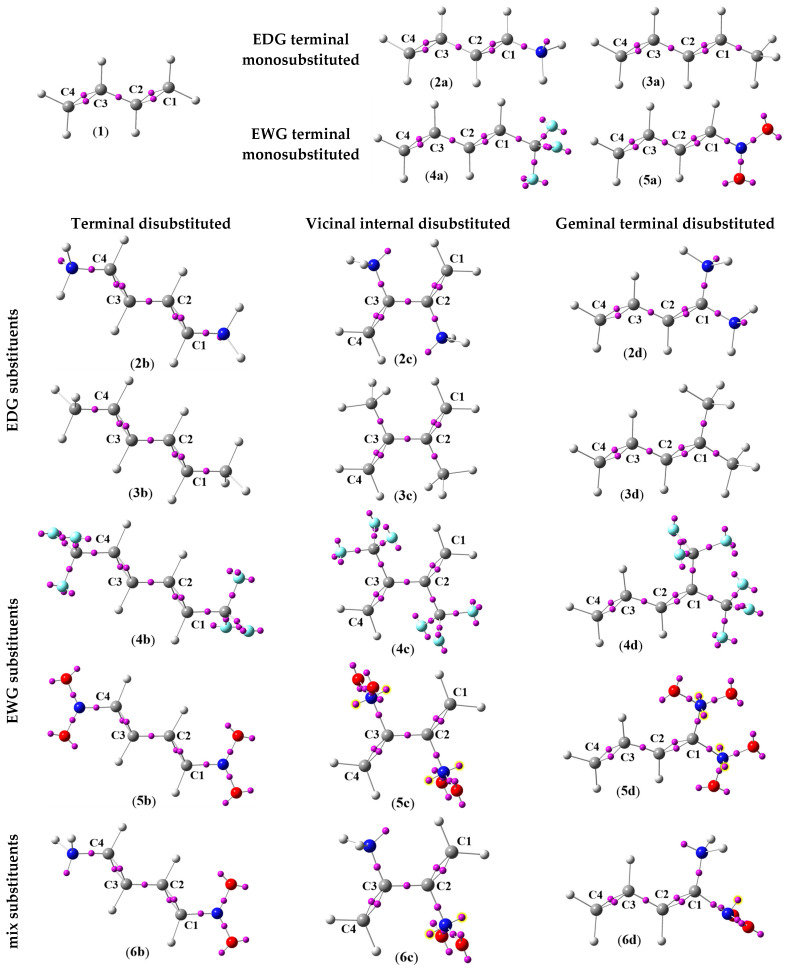
ELF attractor positions (shown as pink spheres) of the core and valence basins, together with the corresponding atom numbers in the butadiene core, for molecules **1** and **2a**–**6d**.

In the ELF analysis, each bond in the molecule is associated with one or more disynaptic basins. Specifically, V (A,B) denotes the disynaptic basin associated with the bond between atoms A and B. In this, V represents the primary disynaptic basin corresponding to the electron density shared between two bonded atoms, while V’ denotes a secondary or complementary basin that also contributes to the same bond, often associated with π or resonance components. The total electron population of the bond, V_t_, is obtained by summing V and V’ (V_t_ = V + V’), providing a quantitative measure of the electrons localized in that bond. Additionally, ELF analysis allows the identification of lone pair electrons in monosynaptic basins and the mapping of electron delocalization in conjugated or aromatic systems, giving a comprehensive picture of electronic structure and reactivity patterns [53,54]. The ELF attractor positions of the core and valence basins for 1,3-butadiene (**1**) and its analogues (**2a**–**6d**) are shown in Figure 5, while the most relevant valence basin populations are given in Table 1.

In the ELF topology of the model conjugated diene, which is 1,3-butadiene (**1**), two sets of disynaptic basins can be distinguished: V (C1,C2) together with V’ (C1,C2), and V (C3,C4) together with V’ (C3,C4) (Figure 5). Each of these basins integrates to an electron population of 1.72 e, giving a combined value of 3.44 e for each bond (Table 1). The occurrence of these pairs reflects the somewhat reduced electron density of the equivalent C1-C2 and C3-C4 double bonds. By contrast, the disynaptic basin V (C2,C3) (Figure 5), which integrates to 2.30 e (Table 1), corresponds to a slightly enhanced electron population in the C2-C3 single bond. Consequently, the ELF analysis clearly indicates that the terminal C1-C2 and C3-C4 bonds, although formally double, show a diminished double-bond character, whereas the central C2-C3 bond possesses an electron distribution more typical of a single bond.

The introduction of EDG or EWG terminal monosubstitution to the structure of 1,3-butadiene (**1**) changes the distribution of electron populations in significant fragments of the structures (**2**–**5a**). In particular, for each compound (**2**–**5a**), an increase in the degree of overpopulation of the central C2-C3 single bond is observed, with the strongest increase for the extreme substituents such as amino (**2a**) and nitro (**2d**) (Table 1). The disappearance of equivalent electron populations between C1-C2 and C3-C4 atoms is also noticeable, whereby the total electron population is always higher in the case of interaction of the C1-C2 atoms, where the substituent is located. This is a consequence of the disruption of the structure of the simplest conjugated diene (**1**). These differences are practically unobservable in the case of 2,5-pentadiene (**3a**), for which the introduction of a methyl group does not cause significant changes. The electron population distribution for compound (**3a**) is the most similar to that of 1,3-butadiene (**1**) (Table 1). Greater differences in the electron population between C1-C2 and C3-C4 atoms occur in the case of 1-amino-1,3-butadiene (**2a**) and 1-trifluoromethyl-1,3-butadiene (**4a**), where the influence of substituents causes differences of ca. 0.08 e (Table 1). However, the strongest disorder of the C1-C2 and C3-C4 equivalent double bond system is observed for 1-nitro-1,3-butadiene (**5a**). In particular, the difference in the electron population for this diene is 0.16 e (Table 1). Such a strong interaction of the nitro group with the conjugated π-bond system is not uncommon and is also observed for less structurally expanded nitroalkenes [55,56,57,58].

The nature of all tested 1,3-butadiene analogues, substituted in both terminal (**2**–**5b**) as well as internal (**2**–**5c**) positions, is practically identical to their monosubstituted counterparts (**2**–**5a**). In particular, compounds **2**–**5b** and **2**–**5c** are characterized by a higher electron population, both for the internal C2-C3 single bond as well as the C1-C2 and C3-C4 double bonds, compared to the simplest conjugated diene (**1**) (Table 1). Among these compounds, it is worth noting that amine derivatives **2b** and **2c** stand out. The total electron populations between C1-C2 and C3-C4 atoms in the 1,4-diamino-1,3-butadiene (**2b**) take the most similar form to a typical double bond (Table 1). On the other hand, in the 2,3-diamino-1,3-butadiene (**2c**), the electron population concentrates around the C2-C3 single bond (Table 1). This perfectly illustrates how the substitution in the conjugated π-bond system can influence the electron location. However, of this group of compounds, the most interesting turned out to be 2,3-dinitro-1,3-butadiene (**5c**). The vicinal internal position of the nitro groups does not relevantly affect the electron populations between the C1-C2 and C3-C4 atoms, but it causes a significant increase in the electron population in the center of the butadiene core between the C2-C3 atoms, to 2.30 e (Table 1). What is also interesting is that this kind of substitution causes the appearance of two monosynaptic basins on both nitrogen atoms of the nitro groups (Figure 5). Each of these basins integrates a population of 0.33 e, which in effect causes the presence of a population of 0.66 e, located on each nitrogen atom in the molecule (**5c**).

The presence of two substituents, located at the same terminal carbon atom, causes significant changes in electron localization. In contrast to the previously discussed examples, compounds (**2**–**5d**) are characterized by the highest degree of disturbance present in electron population in the whole C1-C2-C3-C4 butadiene core. However, the trend of electron population distribution that was observed for terminally monosubstituted analogues of 1,3-butadiene (**2**–**5a**) is still preserved. In particular, the increase in electron population, both between C2-C3 and C1-C2 atoms, is noticeable. Simultaneously, the difference in electron populations between C1-C2 and C3-C4 atoms for compound (**3d**) is the lowest, 0.09 e; it increases for compounds (**2d**) and (**4d**), 0.22 e, up to the value of 0.52 e for compound (**5d**) (Table 1). What is more, in the molecule (**5d**), two monosynaptic basins on both nitrogen atoms of the nitro groups are present (Figure 5). Each of these basins integrates a population of 0.30 e, which in effect causes the presence of a population of 0.60 e, located on each nitrogen atom in the molecule (**5d**).

The effect of the introduction of two extremely EDGs or EWGs in the form of amino or nitro groups, respectively, in the structures of 1,3-butadiene analogues (**6b**–**d**) cannot be clearly defined. For terminal substitution in 1-nitro-4-amino-1,3-butadiene (**6b**), the electron population distribution is higher in between the C3-C4 atoms where the amino group is located (Table 1). This proves that the amino group is an electron donor, while the nitro group is an electron acceptor, and along the whole C1-C2-C3-C4 butadiene core there is a flow of electrons. However, the electron population values exclude the *push–pull* effect [59,60]. In the case of 2-nitro-3-amino-1,3-butadiene (**6c**), a similar trend of increase of the electron population in the whole C1-C2-C3-C4 butadiene core is noticeable, with the highest value being observed between the C3-C4 atoms, in the neighborhood of the amino group (Table 1). On the other hand, the location of both mentioned EDG and EWG groups on the same carbon atom causes that in the 1-amino-1-nitro-1,3-butadiene (**6d**) the highest concentration of electron population is observed in the neighborhood of both these groups (Table 1). It is also worth noting that, similarly to the case of the dinitro-substituted 1,3-butadiene (**5c**) and (**5d**), compounds (**6c**) and (**6d**) have monosynaptic electron basins located on the nitrogen atoms of the nitro groups (Figure 5). Thus, the 2-nitro-3-amino-1,3-butadiene (**6c**) has two monosynaptic basins, integrating together a population of 0.54 e, while in the case of 1-amino-1-nitro-1,3-butadiene (**6d**), only one monosynaptic basin, integrating a population of 0.23 e, is observed.

It should be underlined that the presence of monosynaptic basins, located on the nitrogen atom of nitro groups in compounds (**5c,d**) and (**6c,d**), is not a rare phenomenon [61,62]. The phenomenon is significant from the viewpoint of the reactivity of such molecules. The simultaneous occurrence of a conjugated π-bond system and a strongly electron-withdrawing nitro group stimulates the aforementioned phenomenon [63,64]. As a result, these kinds of compounds are an excellent component for many chemical processes, including the hetero Diels–Alder (HDA) reactions [65,66,67].

**Figure 6:**
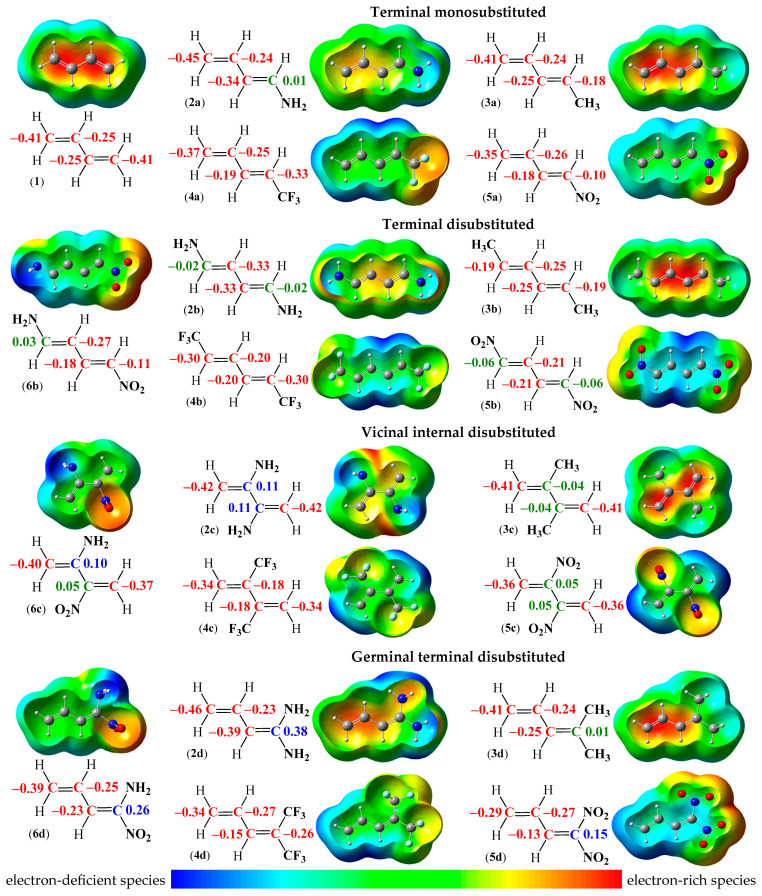
Proposed ELF-based Lewis-like structures with the natural atomic charges for molecules **1** and **2a**–**6d** (given an average number of electrons, **e**) together with the MEP maps. Positive charges are colored blue, negative charges are colored red, while negligible charges are colored green.

To sum up the ELF analysis, it can be concluded that despite the differences occurring in the electron populations of all considered compounds, the conjugated π-bond system is preserved in the whole C1-C2-C3-C4 butadiene core. While the ELF topological analysis provides a bonding pattern concordant with the commonly accepted Lewis structure, the NPA represents the reagents’ electronic structure [68,69]. Therefore, NPA and MEP [70] analyses were carried out. The proposed structures, together with the natural atomic charges as well as the molecular electrostatic potential maps for all considered compounds, are shown in Figure 6.

The NPA of the model conjugated diene, which is 1,3-butadiene (**1**), reveals a negative charge distribution spread across the entire butadiene core. Among the carbon atoms, the terminal positions carry a more pronounced negative charge (−0.41 e), while the internal carbons are less electronegative, with charges of −0.25 e (Figure 6). These findings are consistent with the MEP results, which show a strongly negative electrostatic potential localized around all carbon atoms of the simplest diene (**1**) (red region), contrasted with weaker electron density in the vicinity of the hydrogen atoms (green–blue regions) (Figure 6).

The NPA results of terminally monosubstituted analogues of 1,3-butadiene (**2**–**5a**) show that for the terminal carbon atoms, not connected with the substituent, highly electronegative regions are still present. What is more, values of the natural atomic charges for both of the centric carbon atoms continuously oscillate around 0.25 e, with the highest differences occurring in the case of compounds **2a** and **5a** (Figure 6). Finally, the presence of a substituent, directly connected with a carbon atom, causes depletion of the electronegative region in all cases, with the strongest effect for the 1,3-butadiene with a terminal amino group (**2a**) and the weakest for the 1,3-butadiene with a terminal trifluoromethyl moiety (**4a**) (Figure 6).

Terminal substitution with two groups of the same type practically does not affect the natural atomic charges in the butadiene core. In particular, bilateral chain extension by adding methyl groups in compound **3b** has no effect, while in the case of both compounds **4b** and **5b**, including EDG, a slight electron depletion is observable (Figure 6). Only the presence of amino groups in compound **2b** somewhat increases an electron population (Figure 6). On the other hand, the different effect for the terminal atoms of the butadiene core is noticeable. In each case, regardless of the type of substitution, the high electronegative regions present in 1,3-butadiene (**1**) are noticeably reduced, even to negligible values in the case of compounds **2b** and **5b** (Figure 6).

The effect of the introduction of two extreme EDGs and EWGs in a form of amino and nitro groups at both terminal positions in 1-nitro-4-amino-1,3-butadiene (**6b**) confirms the results of ELF analysis. The compound (**6b**) shows no signs characteristic of a *push–pull* structure. In particular, for both terminal atoms in the 1,3-butadiene core of compound **6b**, natural atomic charges are reduced to 0.03 e and −0.11 e, compared to the simplest conjugated diene (**1**) (Figure 6). This observation is inconsistent with the basic assumption of the NPA distribution for *push-pull* structures, in which an increase in electron population for one terminal atom and a reduction in electron population for the second terminal atom, in the structural fragment under consideration, are noticeably simultaneous [71,72].

In comparison with the terminal disubstitution, a different dependence of the distribution of natural atomic charges is observed for vicinal centric disubstitution. The smallest difference against the simplest conjugated diene (**1**) occurs in the compound **4c**, where a slight electron depletion is noticeable in the whole C1-C2-C3-C4 butadiene core (Figure 6). In the case of compounds **3c** and **5c**, depletion of the electronegative region to practically negligible charges is noticeable. In turn, in compound **2c**, vicinal centric carbon atoms of the 1,3-butadiene core are characterized by a slightly positive charge (Figure 6). It also should be noted that natural atomic charges for the rest of the terminal carbon atoms of the 1,3-butadiene core are barely changed (Figure 6). The interesting fact here is that both amino and nitro groups cause the same effect of decrease in electron populations compared to the simplest conjugated diene (**1**). In turn, values of electron populations for the 2-nitro-3-amino-1,3-butadiene (**6c**) are practically perfectly mapped with regard to the fragments of both compounds **2c** and **5c** (Figure 6).

The greatest changes in the distribution of electron populations for compounds **2**–**5d** are observed in the case of geminal terminal disubstitution, and this effect cannot be clearly defined. In particular, the least noticeable changes occur for 1,3-butadiene analogue **4d**. The NPA for this compound (**4d**) indicates a negative distribution located in the whole butadiene core (Figure 6). For compound **3d,** the distribution of electron population along the 1,3-butadiene core is practically identical to that of the simplest conjugated diene (**1**). The only difference appears for the carbon atom connected to the two methyl groups, where the natural atomic charge is reduced to negligible values (Figure 6).

In turn, both compounds **2d** and **5d** with EDG and EWG in the form of amino and nitro groups, respectively, indicate a positive distribution located on the terminal carbon atom, directly connected to the substituent (Figure 6). On the other hand, the second terminal carbon atom in both compounds **2d** and **5d** is characterized as the highest population of electrons in the whole C1-C2-C3-C4 butadiene core (Figure 6). It means that for these molecules (**2d** and **5d**), the *push* effect is noticeable. In addition, an analogous situation in the case of 1-amino-1-nitro-1,3-butadiene (**6d**) is observed; moreover, for this compound (**6d**), the strength of the effect is in between that of the molecules **2d** and **5d** (Figure 6).

#### 2.1.2. Analysis of the CDFT Reactivity Indices

CDFT is a very important tool in understanding the reactivity of molecules in various processes. The theory connects well-established chemical concepts, like electronic chemical potential μ and chemical hardness η, as well as chemical softness S, with the electronic structure of a molecule. The presented reactivity descriptors are important due to their ability to quantify the tendency of a molecule to donate or accept electrons and to predict its overall chemical reactivity, which can in turn provide insights into potential biological interactions, such as binding to biomolecules, enzyme reactivity, or participation in redox processes. In particular, the electronic chemical potential informs about the tendency of a molecule to gain or lose electrons, which can influence its interactions with biological targets; chemical hardness indicates the resistance of a molecule to changes in its electron number, reflecting its stability; and chemical softness shows the ease with which a molecule can undergo electronic rearrangements, correlating with its potential reactivity in biological processes, including binding, catalysis, or redox reactions [73,74]. Based on those, the indication of global electronic properties of substrates, such as global electrophilicity ω (a measure of the molecule’s ability to accept electrons) and global nucleophilicity N (a measure of the molecule’s tendency to donate electrons) for molecules, can be established. As an effect, it is possible to assign addends a role of either an electrophile or a nucleophile in the studied reactions [75,76,77]. Furthermore, with the application of Parr functions, not only global but also local electronic properties of a molecule can be estimated, thus allowing for the prediction of reactivity of molecules in the studied reactions based only on substrate structures [78,79].

**Figure 7:**
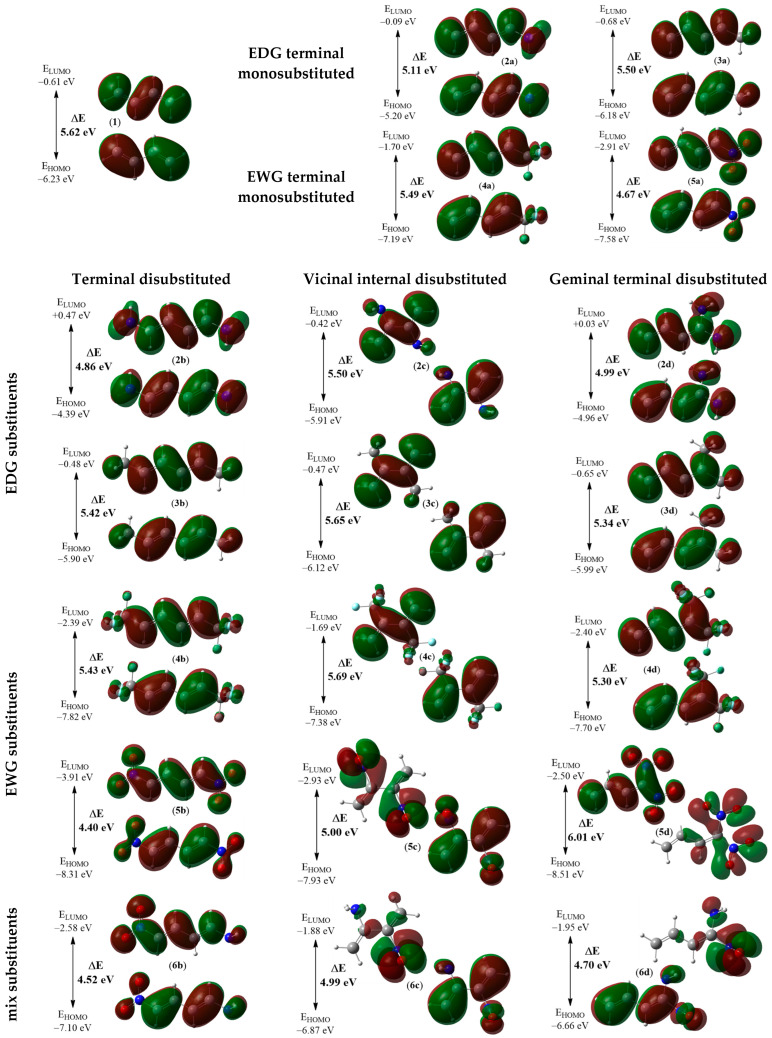
HOMO-LUMO energy gap diagram for molecules **1** and **2a**–**6d**.

The calculation of reactivity descriptors is based on the energy values of the frontal molecular orbitals (MOs) HOMO and LUMO for the compound. It should be noted that the difference in values between the mentioned orbitals alone provides significant information. The HOMO-LUMO energy gap is an important stability index, as it explains the charge transfer interactions within the molecule and is useful in determining molecular electronic transport properties. A molecule with a high frontier orbital (HOMO-LUMO) energy gap has low chemical reactivity and simultaneously high kinetic stability. The phenomenon is related to high excitation energy between the high-lying LUMO and the low-lying HOMO energy levels [80,81,82]. Therefore, the values of HOMO and LUMO energies for 1,3-butadiene (**1**) and its analogues (**2a**–**6d**), together with the values of the HOMO-LUMO energy gap, are collected in Figure 7.

The computed HOMO energy [83] of the model conjugated diene, which is 1,3-butadiene (**1**), is −6.23 eV, and the computed LUMO energy [83] for this molecule (**1**) is −0.61 eV. Consequently, the value of the HOMO-LUMO energy gap is 5.62 eV (Figure 7). The introduction of EDG or EWG in the structures of terminal monosubstituted analogues of 1,3-butadiene (**2**–**5a**) causes depletion of the values of the HOMO-LUMO energy gap, regardless of the type of substituent. It means that all compounds (**2**–**5a**) are simultaneously less stable and more reactive, with the most reactive being 1-nitro-1,3-butadiene (**5a**) (Figure 7). On the other hand, both terminally substituted 1,3-butadiene analogues with methyl (**3a**) or trifluoromethyl (**4a**) groups have the value of the HOMO-LUMO energy gap similar to the simplest conjugated diene (**1**). Only for 1-amino-1,3-butadiene (**2a**) is the decrease of this value noticeable (Figure 7). These observations indicate that, similarly to the ELF and NPA analyses, the greatest differences occur in the case of substitution of 1,3-butadiene (**1**) with amino (**2a**) and nitro (**5a**) groups.

A similar analogy occurs in terminal disubstitution of 1,3-butadiene (**3**–**5b**), where the values of the HOMO-LUMO energy gap for compounds 3b and 4b are close to the value of the simplest conjugated diene (**1**), and the 1,4-dinitro-1,3-butadiene (**5b**) has the smallest value of the HOMO-LUMO energy gap, so the compound (**5b**) is both the least stable and the most reactive in the entire series (**2**–**5b**) (Figure 7). In turn, for vicinal centric disubstituted analogues of 1,3-butadiene (**2**–**5c**), the values of the HOMO-LUMO energy gap for compounds **2**–**4c** oscillate close to the value for the simplest conjugated diene (**1**). On the other hand, the 2,3-dinitro-1,3-butadiene (**5c**) has the lowest value of the HOMO-LUMO energy gap in the entire series (**2**–**5c**) (Figure 7).

In the case of geminal terminally disubstituted analogues of 1,3-butadiene (**2**–**5d**), the trend in values of the HOMO-LUMO energy gap for compounds **2**–**4d** is similar to the series of terminal disubstituted compounds (**2**–**4b**). The most significant difference is related to the noticeable increase in the value of the HOMO-LUMO energy gap for the 1,1-dinitro-1,3-butadiene (**5d**), which surpasses the others (Figure 7). This observation indicates that the compound (**5d**) is simultaneously the most stable and the least reactive in the entire series (**2**–**5d**). This fact is interesting from the point of view of the stability of nitro compounds. As is well known, with the increase in the number of nitro groups in the molecule, the stability of such compounds decreases [36]. For terminal mono- and disubstituted 1,3-butadiene **5a** and **5b,** this trend is observed. In turn, for the rest of two compounds, **5c** and **5d**, higher values of the HOMO-LUMO energy gap may result from the high degree of unsaturation of the molecule and, consequently, the specific arrangement of delocalized π-electrons in the whole butadiene core, caused by the presence of various substituents.

The presence of two EDG or EWG in the form of amino as well as nitro groups, respectively, in the structures of 1,3-butadiene analogues **6b**–**d** does not significantly affect the values of the HOMO-LUMO energy gap. In particular, in the case of terminally disubstituted 1-nitro-4-amino-1,3-butadiene (**6b**), the effect is similar to compounds with amino (**2b**) and nitro (**5b**) moieties simultaneously (Figure 7). In turn, the 2-nitro-3-amino-1,3-butadiene (**6c**) presents properties close to its vicinally centric disubstituted analogue in the form of dinitro derivative **5c** (Figure 7). The smallest value of the HOMO-LUMO energy gap occurring in the entire series **6b**–**d** is observed in the case of substitution with amino and nitro groups in the geminal terminal position. Thus, 1-amino-1-nitro-1,3-butadiene (**6d**) is both the least stable and the most reactive (Figure 7).

**Figure 8:**
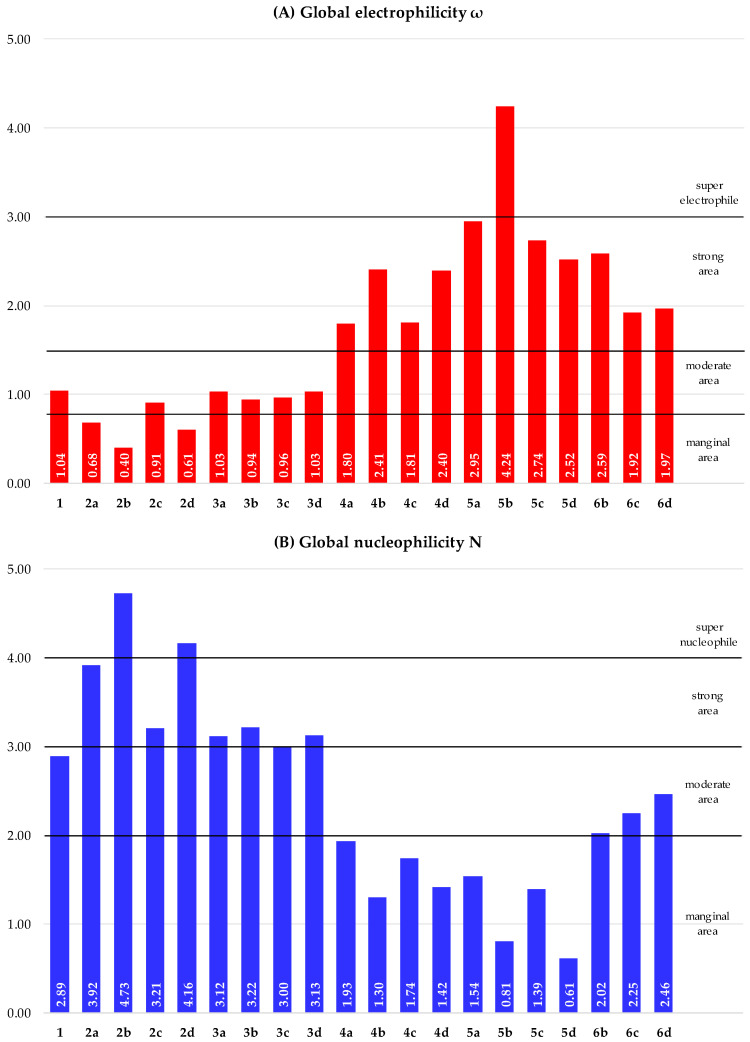
The visualization of the global electrophilicity (**A**) and nucleophilicity (**B**) for molecules **1** and **2a**–**6d**. The values are given in electronvolts, eV.

Based on HOMO as well as LUMO energies of 1,3-butadiene (**1**) and its analogues (**2a**–**6d**), the main reactivity indices were calculated and are given in Table 2. Finally, based on obtained data, the values of global electrophilicity and global nucleophilicity were determined and are presented in the form of a visualization in Figure 8.

The calculated global electrophilicity (ω) [84] index of the model conjugated diene, which is 1,3-butadiene (**1**), is 1.04 eV, while its global nucleophilicity (N) [85] index is 2.89 eV (Figure 8). Based on these values, molecule **1** can be categorized simultaneously as a compound of moderate electrophilic as well as moderate nucleophilic character in polar reactions, according to the established electrophilicity/nucleophilicity scale [86,87]. The substitution of 1,3-butadiene (**1**) changes both global electrophilicity and global nucleophilicity in all considered compounds, according to a specific way, regardless of the number of substituents introduced and how they are attached. Thus, the introduction of an amine substituent to 1,3-butadiene (**1**) in structures (**2a**–**d**) results in a significant reduction of the global electrophilicity and a significant increase of the global nucleophilicity (Figure 8). As a consequence, these compounds (**2a**–**d**) can be classified as marginal electrophiles and, at the same time, as strong nucleophilic species. The replacement of the amino group in compounds **2a**–**d** by a less EWG methyl moiety in structures (**3a**–**d**) causes a slight increase in global electrophilicity and, at the same time, a slight reduction in global nucleophilicity (Figure 8). Therefore, these compounds (**3a**–**d**) can be classified as moderate electrophiles, similarly to 1,3-butadiene (**1**); however, they will have the character of strongly nucleophilic species.

On the other hand, the introduction of the EDG trifluoromethyl group in compounds 4a–d causes both a noticeable increase of global electrophilicity as well as a reduction of global nucleophilicity compared to other compounds with amine (**2a**–**d**) and methyl (**3a**–**d**) moieties (Figure 8). Consequently, these compounds (**4a**–**d**) can be classified as strong electrophiles as well as marginal nucleophiles. The replacement of the trifluoromethyl group in compounds **4a**–**d** by a more EDG nitro moiety in structures **5a**–**d** causes both a noticeable increase of global electrophilicity as well as a reduction of global nucleophilicity (Figure 8). As a result, all compounds (**5a**–**d**) still can be classified as strong electrophiles as well as marginal nucleophiles.

The position of both the amino and nitro groups in compounds 6b–d does not significantly influence the value of the global electronic properties. In particular, the terminal position in 1-nitro-4-amino-1,3-butadiene (**6b**) determines the strongest electrophilic character in the series (**6b**–**d**), while simultaneously the weakest nucleophilic character (Figure 8). Both the vicinally internal and geminal terminal substitutions for 2-nitro-3-amino-1,3-butadiene (**6c**) and 1-amino-1-nitro-1,3-butadiene (**6d**) are characterized by similar values of global electrophilicity, and their global nucleophilicities differ only slightly (Figure 8). Regardless of this, all considered compounds (**6b**–**d**) can be classified as strong electrophiles as well as moderate nucleophiles.

Unlike the series (**6b**–**d**), for the remaining compounds (**2a**–**5d**), the position of the substituent is significant. It can be observed that the most extreme character of the molecule occurs in the case of terminal positioning (**2**–**5b**). In particular, 2,4-hexadiene (**3b**) has the highest value of global nucleophilicity, N = 3.22 eV (Figure 8B), and the lowest value of global electrophilicity, ω = 0.94 eV (Figure 8A), among compounds (**3a**–**d**).

In turn, 1,4-trifluoromethyl-1,3-butadiene (**4b**) is characterized as having the best electrophilic properties, ω = 2.41 eV (Figure 8A), and the worst nucleophilic properties, N = 1.30 eV (Figure 8B), among compounds (**4a**–**d**). Based on both the HOMO and LUMO energy values as well as the reactivity descriptor values, it can be concluded that compounds (**2b**) and (**5b**) are the most suitable reaction components. Both compounds (**2b**) and (**5b**) are characterized by low HOMO-LUMO energy gap values (Figure 7), indicating their high reactivity [80,81,82]. Moreover, among all analyzed 1,3-butadiene analogues (2a–**6d**), the 1,4-diamino-1,3-butadiene (**2b**) has the highest global nucleophilicity value, N = 3.92 eV (Figure 8B), while 1,4-dinitro-1,3-butadiene (**5b**) has the highest global electrophilicity value, ω = 4.24 eV (Figure 8A). Simultaneously, the global electrophilicity value for 1,4-diamino-1,3-butadiene (**2b**) is ω = 0.40 eV (Figure 8A), while the global nucleophilicity value for 1,4-dinitro-1,3-butadiene (**5b**) is N = 0.81 eV (Figure 8B). Such extreme differences in the values of global reactivity parameters mean that compound (**2b**) can be assigned the role of a super nucleophile, while compound (**5b**) can be assigned the role of a super electrophile, regardless of the choice of the second reagent [88].

**Figure 9:**
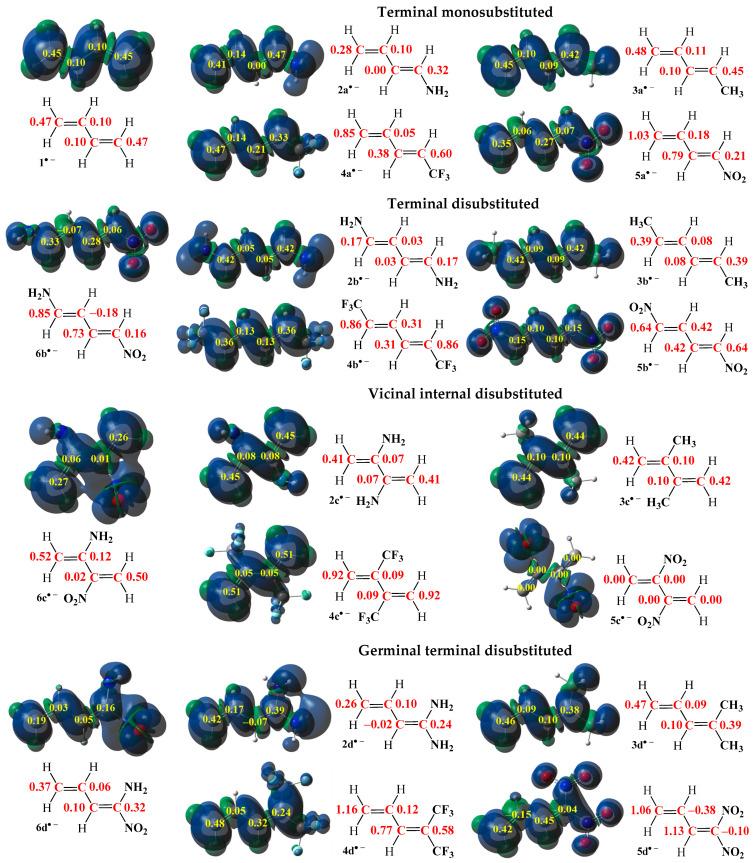
Local electronic properties of molecules **1** and **2a**–**6d** presented as three-dimensional representations (3D) of Mulliken atomic spin densities for radical anions together with the electrophilic P_k_^+^ Parr functions values (given in yellow) as well as the indices of the local electrophilicity ω_k_ (given in red, in eV).

**Figure 10:**
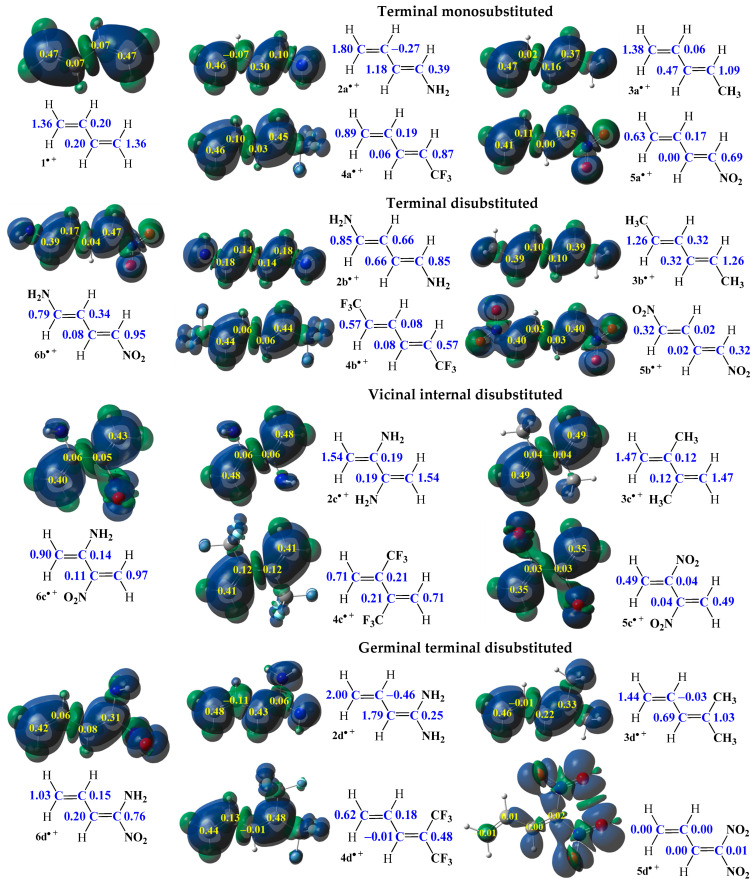
Local electronic properties of molecules **1** and **2a**–**6d** presented as three-dimensional representations (3D) of Mulliken atomic spin densities for radical cations together with the nucleophilic P_k_^−^ Parr functions values (given in yellow) as well as the indices of the local nucleophilicity N_k_ (given in blue, in eV).

The shown information is particularly important, since in reactions involving non-symmetrical reagents, regioselectivity is determined by the interaction between the most electrophilic center of the electrophile and the most nucleophilic center of the nucleophile [89]. In order to identify these reactive sites in the model 1,3-butadiene (**1**) and its analogues (**2a**–**6d**), the electrophilic P_k_^+^ and nucleophilic P_k_^−^ Parr functions, along with local electrophilicities ω_k_ and local nucleophilicities N_k_, were computed and are summarized in Figure 9 and Figure 10 [90].

The analysis of the electrophilic P_k_^+^ Parr function [91] of the simplest conjugated diene (**1**) indicates that the terminal carbon atoms are the most electrophilic centers of this species, both presenting the value P_C_^+^ = 0.45. At these atoms, the value of the local electrophilicity ω_k_ index is ω_C_ = 0.47 eV (Figure 9). In turn, electrophilic P_k_^+^ Parr functions [91] for the centric atoms of this diene (**1**) are reduced to P_k_^+^ = 0.10 (ω_C_ = 0.10 eV) (Figure 9). On the other hand, analysis of the nucleophilic P_k_^−^ Parr functions of 1,3-butadiene (**1**) indicates that the terminal carbon atoms are also the most nucleophilic centers of this species, presenting the value P_C_^−^ = 0.47. At these atoms, the value of the local nucleophilicity N_k_ index is N_C_ = 1.36 eV (Figure 10). In turn, values of nucleophilic P_k_^−^ Parr function for the centric atoms of diene **1** are extremely reduced to P_C_^−^ = 0.07 (N_C_ = 0.20 eV) (Figure 10). The differences between nucleophilic properties for non-symmetrical atoms in diene **1** are slightly greater than for electrophilic properties. However, the terminal carbon atoms are both the most electrophilic and nucleophilic centers in the entire molecule.

The smallest differences in local values, both electrophilic and nucleophilic, occur in the case of 1,3-pentadiene (**3a**). In this molecule (**3a**), the terminal carbon atom of the vinyl moiety is both the most electrophilic (ω_C_ = 0.48 eV, Figure 9) and the most nucleophilic (N_C_ = 1.38 eV, Figure 10) center. A similar situation is observed in the case of 1-trifluoromethyl-1,3-butadiene (**4a**), where the terminal carbon atom of the vinyl moiety is also both the most electrophilic (ω_C_ = 0.85 eV, Figure 9) and the most nucleophilic (N_C_ = 0.89 eV, Figure 10) center. Another preference occurs for both compounds **2a** and **5a**. In particular, in 1-amino-1,3-butadiene (**2a**), the introduction of the amino group in one of the terminal positions results in this atom being the most electrophilic center (ω_C_ = 0.32 eV, Figure 9), while the most nucleophilic center (N_C_ = 1.80 eV, Figure 10) will remain on the second terminal atom of the vinyl moiety. Finally, in the case of 1-nitro-1,3-butadiene (**5a**), the introduction of the nitro group in one of the terminal positions causes a different effect: the 1-terminal carbon atom is the most nucleophilic center (N_C_ = 0.69 eV, Figure 10), while the most electrophilic center (ω_C_ = 1.03 eV, Figure 9) will remain on the second terminal atom of the vinyl moiety. Due to the greatest differences between local electronic properties, the best candidates as substrates for reactions are molecules: **2a** as a nucleophile and **5a** as an electrophilic agent.

A similar trend is observed in the case of geminal terminal substitution (**2**–**5d**), where the differences between the values of local electrophilicity and nucleophilicity are generally strengthened. The mentioned tendency is clearly observed in the case of compounds **3d** and **4d**, where the differences of local electronics between terminal atoms in the whole C1-C2-C3-C4 butadiene core are higher than in monosubstituted analogues **3a** and **4a**. In particular, in the case of 2-methyl-2,5-pentadiene (**3d**), the terminal carbon atom of the vinyl moiety is both the most electrophilic (ω_C_ = 0.47 eV, Figure 9) and the most nucleophilic (N_C_ = 1.44 eV, Figure 10) center. In addition, in 1,1-ditrifluoromethyl-1,3-butadiene (**4d**), the terminal carbon atom not adjacent to trifluoromethyl groups is also both the most electrophilic (ω_C_ = 1.16 eV, Figure 9) and the most nucleophilic (N_C_ = 0.62 eV, Figure 10) center. In turn, in 1,1-diamino-1,3-butadiene (**2d**), the terminal carbon atom not adjacent to the amino groups is both the most electrophilic (ω_C_ = 0.26 eV, Figure 9) and the most nucleophilic center (N_C_ = 2.00 eV, Figure 10) in this molecule. The differences in the electrophilic properties between terminal carbon atoms are slight, while those between nucleophilic parameters are significant and greater in molecule **3d** than for molecule **2d** (Figure 10). The conclusion about electrophilic properties is that in contrast to the observations for compound **2a**, in which the most electrophilic center is located at the second terminal carbon atom (Figure 9). Nevertheless, the differences in local properties for both compounds **2a** and **2d** are so small that it is impossible to unambiguously determine which position will be more electrophilically preferred. At the end, in the case of 1,1-nitro-1,3-butadiene (**5d**), the presence of two nitro groups at the same terminal carbon atom causes practically the same effect as for the molecule **2d**. In particular, the terminal carbon atom not adjacent to these nitro groups is the most electrophilic center (ω_C_ = 1.06 eV, Figure 9), while the most nucleophilic center is located at the second terminal carbon atom (N_C_ = 0.01 eV, Figure 10); however, the negligible nucleophilicity present at this atom generally makes compound **5d** an unsuitable candidate as a nucleophilic agent in chemical reactions [92].

In the case of symmetrical molecules in the terminal disubstituted series (**2**–**5b**) as well as the vicinally centrically disubstituted series (**2**–**5c**), the most reactive center will always be the terminal carbon atoms. The presented observation is interesting due to the fact that this preference is not dependent on the type of substituents, nor the positions of these substituents (centric or terminal), nor even on the electrophilic (Figure 9) or nucleophilic (Figure 10) nature of the molecules (**2**–**5b** and **2**–**5c**). What is more, the differences in values of local electrophilicities (Figure 9) and nucleophilicities (Figure 10) of considered compounds **2**–**5b** and **2**–**5c** in the whole C1-C2-C3-C4 butadiene core practically exclude reactions involving centric carbon atoms [92].

The presence of two EDGs or EWGs in the form of amino as well as nitro groups in the structure of 1,3-butadiene analogues in the terminal positions for 1-nitro-4-amino-1,3-butadiene (**6b**) as well as vicinal centric positions for 2-nitro-3-amino-1,3-butadiene (**6c**) makes a similar situation as in the case of series **2**–**5b** and **2**–**5c**. The terminal carbon atoms are the most reactive centers. Taking into account the effect of two different substituents, it can be noted the terminal carbon atoms adjacent to amino groups are the most electrophilic centers (ω_C_ = 0.85 eV for **6b** and ω_C_ = 0.52 eV for **6c**, Figure 9), whereas the terminal carbon atoms adjacent to nitro groups are the most nucleophilic centers (N_C_ = 0.95 eV for **6b** and ω_C_ = 0.97 eV for **6c**, Figure 10). Thus, it should be concluded that along the conjugated systems the amino and nitro substituents interact with each other [64]. In the case of terminal substitution, the differences are more pronounced. At last, the geminal terminal substitution for 1-amino-1-nitro-1,3-butadiene (**6d**) makes the terminal position of the carbon atom not bonded to the substituents attractive as both an electrophilic (ω_C_ = 0.37 eV, Figure 9) and a nucleophilic (N_C_ = 1.03 eV, Figure 10) center.

A thorough analysis of the electronic properties of a molecule is essential for understanding its potential reactivity and the sites that may interact with biomolecules. This information allows the prediction of which molecular fragments can bind to receptors or enzymes, thereby influencing the predicted biological activity as well as pharmacokinetic properties.

### 2.2. Biological Potential Based on ADME and PASS Studies

Since 1,3-butadiene (**1**) is an important raw material for industry [11,12,13,14,15,16,17,18,19,20,21,22,23,24,25,26], its impact of exposure on organisms has been studied since the 1990s [93,94]. Both this compound (**1**) and its metabolites are toxic and mutagenic substances [95,96,97]. Nevertheless, there is some research on the application of 1,3-butadiene analogues as potential biologically active compounds. In this context, trifluoromethyl as well as nitro-substituted compounds received special attention. *Aydinli* [98] and *Deniz* with coworkers [99] described various nitro group–substituted perhalogenobuta-1,3-dienes that can be successfully used against bacteria such as *Escherichia coli*, *Staphylococcus aureus*, and *Mycobacterium luteum*, as well as fungi *Candida tenuis* and *Aspergillus niger*. In 1970, *Durden* et al. [100] tested 1,4-dinitro-1,3-butadiene (**5b**) against *Uromyces phaseoli*. According to in vivo results, the authors reported that this compound (**5b**) is a promising candidate to be applied in the agrochemical industry as a means to prevent bean rust [100].

Moreover, the conjugated system together with EDG and EWG substituents is widely and commonly used as a building block in the synthesis of compounds with biological potential, such as carbazoles, benzoxazolines, benzimidazolines, pyrimidines, imidazolidines, thiazolidinones, pyrazoles, pyrimidines, isothiazoles, dihydroisoxazoles, thiophenes, benzo[h]quinolones, and others [101,102]. For example, nitroperchloro-butadienes are important precursors in the synthesis of ketene dithioacetal analogues, which demonstrate anticonvulsant action comparable with valproic acid and prolonged antidepressant-like properties comparable with a reference drug, Amitriptyline [103]. Other polyhalogenated nitrobutadienes are also used as building blocks to obtain conjugated systems that can be applied as candidates to counteract the negative inflammatory response triggers by human IFN in some autoimmune diseases like systemic lupus erythematosus and amyotrophic lateral sclerosis, among others, where the production of this cytokine is exacerbated and is considered to be one of the causes of their etiopathologies [104,105].

In view of the above, we decided to prepare a simple in silico consideration of possibilities and potential biological applications of 1,3-butadiene (**1**) and its analogues (**2a**–**6d**). To determine the basic physicochemical as well as selected pharmacokinetic properties, the SwissADME web tool [106] has been used. The obtained parameters were then evaluated according to rules of *Lipinski* et al. [107], *Ghose* et al. [108], *Veber* et al. [109], *Egan* et al. [110], and *Muegge* et al. [111]. At the end, potential directions of application for the considered compounds **1** and **2a**–**6d** were indicated using the PASS 2025 software [112].

#### 2.2.1. Analysis of Pharmacokinetic Properties and Drug-likeness Based on ADME

**Figure 11:**
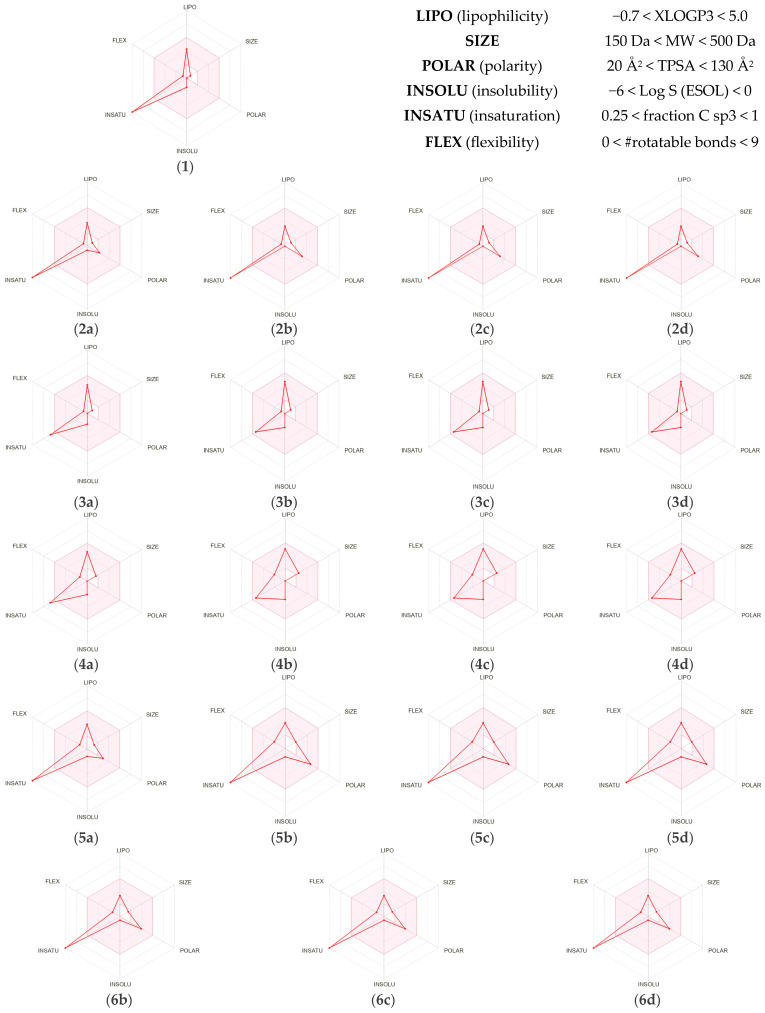
The bioavailability radar of the drug-likeness for molecules **1** and **2a**–**6d**, determined using the SwissADME [113]. The pink area represents the optimal range for each property, like lipophilicity, size, polarity, insolubility, insaturation, and flexibility.

ADME studies play a crucial role in the early stages of drug development. Experimental evaluation of pharmacokinetic parameters is often time-consuming and costly. Therefore, to streamline research efforts and optimize resource allocation, computational tools are increasingly employed for predicting ADME characteristics. In this context, key physicochemical properties such as lipophilicity, solubility, pharmacokinetics, and medicinal chemistry friendliness of the investigated compounds were analyzed. The obtained results of mentioned characteristics for 1,3-butadiene (**1**) and its analogues (**2a**–**6d**) are summarized in Table 3. Additionally, bioavailability radars of these molecules (**1** and **2a**–**6d**) are summarized in Figure 11. Based on the calculated parameters, the drug-likeness of the considered compounds (**1** and **2a**–**6d**) was also evaluated. For this purpose, the freely accessible online platform SwissADME [113] was utilized, as it provides some of the most reliable predictions to support the design of potential drug candidates [114,115].

Based on physicochemical properties and predicted ADME parameters shown in Table 3, it can be concluded that both 1,3-butadiene (**1**) and its considered analogues **2a**–**6d** are promising candidates for medicines based on the filters by *Lipinski* et al. [107], *Veber* et al. [109], and *Egan* et al. [110]. In particular, according to the mentioned rules, all tested compounds (**1** and **2a**–**6d**) have an appropriate low molecular weight (no more than 500 Da), number of rotatable bonds, H-bond donor–acceptor ratio, and satisfactory value of Topological Polar Surface Area (TPSA) (Table 3), which is a parameter describing the total surface area of polar atoms, such as nitrogen, oxygen, or sulfur. The parameter helps predict biological membrane permeability and drug bioavailability. It is generally accepted that TPSA values below 140 Å^2^ favour good oral bioavailability, while values below 90 Å^2^ facilitate penetration of the blood–brain barrier [116,117]. What is more, all tested compounds (**1** and **2a**–**6d**) are characterized by a TPSA below the reference values according to the Veber filter [109] (less than 140 Å^2^) as well as the Egan filter [110] (less than 131.6 Å^2^), so they may have appropriate lipophilic properties [118,119], which constitute a relevant parameter due to the potential bioavailability and efficacy of the hypothetical therapeutic agent [120,121]. However, both the reference 1,3-butadiene (**1**) and its methylated (**3a**–**d**) as well as trifluoromethylated (**4a**–**d**) analogues have a TPSA value of 0 Å^2^ (Table 3). This situation indicates that this kind of compound is completely nonpolar, which favors excellent permeability through lipid membranes, including the blood–brain barrier. However, the absence of polarity limits the ability to form specific and strong interactions with the biological target, potentially reducing biological activity. Moreover, molecules with a TPSA value of 0 Å^2^ may exhibit excessive lipophilicity, leading to nonspecific interactions and an increased risk of toxicity [116,117].

According to more demanding filters such as those developed by *Ghose* et al. [108] and *Muegge* et al. [111], both 1,3-butadiene (**1**) and its considered analogues (**2a**–**6d**) are not good potential candidates as medicaments. This is related to the fact that both mentioned filters define a minimum molecular weight. The *Ghose* filter specifies, among other criteria, that the molecular weight should be in the range of approximately 160–480 Da [108]. The *Muegge* filter allows molecules with a molecular weight in the range of about 200–600 Da [111]. On the other hand, all studied molecules (**1** and **2a**–**6d**) have a molecular weight below 150 Da (Table 3). The minimum molecular weight is important because molecules that are too small often bind non-specifically to many proteins, which can lead to side effects or lack of efficacy. Small molecules may not have enough functional groups to form strong and stable interactions with the biological target. Low molecular weight also favors rapid elimination of the compound from the body before it can produce desired pharmacological effects. Moreover, most active sites of proteins have a specific size, and molecules that are too small may not fill the binding pocket, reducing the strength and specificity of the interaction [122,123].

Low molar mass is a direct consequence of an insufficient number of atoms in the molecule. According to the *Ghose* filter [108], the number of atoms in a molecule should be in the range of approximately 20–70. An appropriate number of atoms is important because it affects the size, polarizability, and the molecule’s ability to form stable and specific interactions with biological targets. Too few atoms may indicate that the molecule is too simple and lacks functionality, limiting its effectiveness, while too many atoms can lead to excessive mass and rigidity, hindering membrane permeability and increasing the risk of nonspecific interactions. The *Muegge* filter [111] also considers the range of total atoms, usually around 10–70; it additionally specifies that the number of carbon atoms should be greater than 4 and the number of heteroatoms greater than 1. This emphasizes the importance of a proper balance between molecular size and chemical complexity in the selection of potential drug candidates [124,125]. While in all compounds the *Muegge* filter [111] concerning the minimum total number of atoms is satisfied, in the case of the *Ghose* filter [108], none of the tested compounds (**1** and **2a**–**6d**) satisfies the required assumption (Table 3). What is more, in the case of the *Muegge* [111] drug similarity rules, neither the reference 1,3-butadiene (**1**) nor its amine (**2a**–**d**) and nitro (**5a**–**d**) analogues contain the recommended number of more than five carbon atoms (Table 3). A similar situation occurs for compounds containing both these groups (**6b**–**d**) (Table 3). In turn, in the case of the methylated (**3a**–**d**) as well as the trifluoromethylated (**4a**–**d**) analogues of the reference 1,3-butadiene (**1**), the minimum carbon atom filter is satisfied, but these compounds (**3a**–**d**) and (**4a**–**d**) do not contain the recommended heteroatoms (Table 3).

The consequence of too low a molecular weight is that all considered compounds (**1** and **2a**–**6d**) have an inappropriate value of the molar refraction parameter. For all tested compounds (**1** and **2a**–**6d**), the values of this parameter are less than 37 (Table 3). Molar refractivity is an important parameter that reflects the polarizability and size of a molecule. This is crucial for its ability to interact with biological targets. According to the *Ghose* filter [108], an appropriate molar refractivity value is within the range of 40–130 cm^3^/mol and indicates an optimal molecular size and flexibility, promoting strong and specific binding to protein active sites. Too low molar refractivity may indicate a molecule that is too small and poorly polarizable, resulting in insufficiently stable interactions and rapid elimination from the body, limiting its pharmacological effectiveness. Conversely, too high molar refractivity suggests a large, rigid, and less flexible molecule; this may hinder membrane permeability, reduce solubility, and increase the risk of unwanted interactions with other proteins, negatively affecting the drug’s bioavailability and safety [126].

The analysis of the rest of the parameters, namely lipophilicity and solubility in water, shows that all considered compounds (**1** and **2a**–**6d**) not only have good lipophilic properties, but they are also water-soluble (Table 3). These physicochemical properties are essential for drug absorption and subsequent metabolism [127,128]. Good lipophilicity enables membrane permeability, while water solubility ensures adequate concentration in bodily fluids. Both properties are essential for effective absorption from the gastrointestinal tract. Additionally, lipophilicity influences metabolic enzyme activity, determining a compound’s stability and elimination [129,130]. In this context, referring to the consensus Log (P_o/w_) parameters [131], which is the average value of the logarithm of the octanol/water partition coefficient [132], it can be observed that most of the computed values oscillate within the range of 0–3 (Table 3). These values constitute the optimal lipophilicity range for most drugs, as they ensure both good water solubility and adequate permeability [133]. Consensus Log (P_o/w_) values between 3 and 5 for ditrifluoromethylated 1,3-butadiene analogues (**4b**–**d**) indicate slightly increased lipophilicity, which facilitates membrane permeation but may increase the risk of accumulation in fatty tissues and toxicity (Table 3). In turn, the most deviating negative consensus Log (P_o/w_) value for 1,4-dinitro-1,3-butadiene (**5b**) may denote a highly hydrophilic compound with limited absorption capacity (Table 3). It is also worth noting that both 1,3-butadiene (**1**) and its selected analogues (**3a**–**d** and **4a**–**d**) show higher lipophilicity values than the rest of the considered compounds (**2a**–**d**, **5a**–**d**, and **6b**–**d**), which is directly related to the TPSA indices (Table 3) [116,117]. Greater lipophilicity is associated with lower water solubility, as lipophilic molecules have a higher affinity for nonpolar environments and a reduced ability to form hydrogen bonds with water. This trend is observed for all tested compounds (**1** and **2a**–**6d**), where, according to the current scale [113,134,135], compounds with Log S above −4 are classified as water-soluble, and their solubility increases with the increase of this parameter. Therefore, both the reference 1,3-butadiene (**1**) and its analogues (**3a**–**d** and **4a**–**d**) can be described as water-soluble, while the remaining considered compounds (**2a**–**d**, **5a**–**d**, and **6b**–**d**) will be characterized by better water solubility (Table 3).

According to predictions based on SwissADME software (http://www.swissadme.ch) [106], it should be noted that all tested compounds (**1** and **2a**–**6d**) are characterized by good gastrointestinal (GI) absorption (Table 3). High GI absorption is crucial because it indicates that the compound can efficiently pass through the intestinal lining and enter systemic circulation, which is essential for oral bioavailability and therapeutic effectiveness [134,135]. What is more, all mentioned compounds (**1** and **2a**–**6d**) do not inhibit any of the presented cytochrome P450 isoforms, which are significant in drug elimination through the process of metabolic biotransformation (Table 3). This is important information due to the fact that the lack of inhibition of cytochrome P450 enzymes suggests lower potential for drug–drug interactions and a favorable metabolic profile. It is crucial for the safety and efficacy of potential drugs [133]. Finally, according to the considered pharmacokinetic parameters presented in Table 3, all tested compounds (**1** and **2a**–**6d**) do not easily permeate the blood–brain barrier. This fact is directly related to insufficient TPSA values for these compounds (**1** and **2a**–**6d**). Compounds that effectively cross the BBB can exert pharmacological effects in the brain, which is crucial for medicines that can be applied as drugs for the central nervous system. On the other hand, for medicines that should not have an effect on the brain, low BBB permeability is desirable, as it reduces the risk of central nervous system side effects [136,137].

The information presented is in perfect agreement with the bioavailability radars generated by the SwissADME software [106]. In particular, it should be underlined that the main problem is observed in the case of the size of molecules as well as their instauration (Figure 11). Only the ditrifluoromethylated (**4b**–**d**) analogues of the reference 1,3-butadiene (**1**) have a molecular weight above the required limit, and for the nitro-substituted compounds (**5b**–**d**) the molecular weight is at the borderline (Figure 11). In addition, the number of carbon atoms with sp^2^ hybridization poses a problem. Only in structures **3b**–**d** and **4b**–**d** is the degree of unsaturation adequate (Figure 11). The flexibility parameter, namely the number of rotatable single bonds in a molecule, depends on the final properties of the molecule. Flexible molecules can more easily adapt to various protein binding sites, which is advantageous for drugs interacting with dynamic or variable molecular targets, such as enzymes or receptors with variable conformation. In turn, rigid molecules often exhibit high selectivity and lower binding entropy, which is advantageous for drugs acting on very specific targets [138]. In this context, both the reference 1,3-butadiene (**1**) as well as its amine (**2a**–**d**) and methyl (**3a**–**d**) analogues are characterized by marginal flexibility properties, while the increase of flexibility is noticeable both in ditrifluoromethylated (**4b**–**d**) and nitro-substituted compounds (**5b**–**d**) (Figure 11). Based on this, it can be assumed that the introduction of either the trifluoromethyl or nitro groups into the compound structure results in increased flexibility. This conclusion is confirmed in the case of compounds **6b**–**d** (Figure 11). Other parameters included in the bioavailability radar, such as polarity (TPSA [116]), lipophilicity (XLOGP3 [139]), and insolubility (ESOL [140]), have already been covered in this chapter, and, therefore, discussion of them is omitted.

#### 2.2.2. Assessment of Biological Potential Based on PASS

Due to the previously mentioned information on potential biological applications of 1,3-butadiene analogues [98,99,100], a prediction based on the PASS tool [46] was performed on all tested compounds (**1** and **2a**–**6d**). In general, the PASS, which is a component of the Way2Drug portal [117], is a simple yet highly versatile computational tool designed to predict the potential biological activity spectrum of a molecule. It enables the preliminary assessment of drug-likeness, possible mechanisms of action, pharmacological and adverse effects, toxicity, and other relevant parameters, thereby providing valuable guidance in the early stages of drug discovery and development [141,142]. Although the PASS program is based solely on 2D structural descriptors, it can be successfully used for the preliminary screening and evaluation of the potential applications of new compounds. Thanks to its rapid prediction speed and high accuracy, PASS provides a reliable starting point for further in silico studies and experimental research. The results from PASS express the probability of a molecule’s activity (Pa) versus inactivity (Pi), with values ranging from 0.000 to 1.000 [143]. A necessary condition for a compound to be considered active is that the probability of activity (Pa) exceeds the probability of inactivity (Pi), (Pa > Pi) [46]. When Pa exceeds 0.700, the compound is considered to have a high probability of exhibiting the predicted biological activity. This threshold is widely regarded as the formal cut-off in PASS-based analyses and serves as an indication that the compound should be prioritized for experimental verification to confirm the predicted activity. Values in the range of 0.500–0.700 indicate a moderate probability of the predicted biological activity, suggesting that the outcome is less certain yet remains potentially valid. In such cases, when supported by additional in silico evidence, experimental validation is recommended to further assess the compound’s potential. Conversely, Pa < 0.500 indicates a low probability that the predicted activity will be confirmed experimentally. Predictions in this range are typically deprioritized in subsequent research stages, unless other compelling factors [144]. In some cases, an additional, less formal threshold of Pa > 0.900 is applied, which narrows the analysis to activity profiles with the highest predicted probability [145]. Thus, the results obtained using the PASS program, expressed as Pa values for the selected biological activities that meet the criteria of Pa > Pi and Pa > 0.900 for all tested compounds (**1** and **2a**–**6d**), are summarized in Table 4. The complete set of activities, including both Pa and Pi values for cases with Pa > 0.700, is provided in Tables S1–S20 in Supplementary Materials.

According to the prediction based on PASS software, it can be concluded that all considered compounds (**1** and **2a**–**6d**) do not have a very wide range of applications in the analyzed area of Pa > 9.000. Altogether, the PASS prediction has shown 26 possible activities for 20 tested compounds (Table 4). This suggests that most of these activities are unique to individual molecules. The most promising applications for considered compounds are applications as inhibitors of aspulvinone dimethylallyltransferase (predicted for eight compounds) [146] as well as fatty-acyl-CoA synthase (predicted for five compounds), which blocks the enzyme responsible for converting free fatty acids into their active acyl-CoA forms, disrupting metabolic pathways such as β-oxidation and lipid biosynthesis, thereby affecting cell growth and energy homeostasis [147]. Other potential applications include antineoplastic activity for breast and lung cancer (each predicted for 4 compounds) (Table 4).

The compounds with the highest potential activities include 1,3-butadiene analogues with methyl groups (**3a**–**d**) (18 potential activities in total) and the trifluoromethyl moiety (**4a**–**d**) (13 potential activities in total) (Table 4). In particular, compounds **4b** (7 potential activities), **3c** (6 potential activities), and **3d** (7 potential activities) deserve special attention (Table 4). This observation offers valuable insight, because both mentioned structural fragments (CF_3_ and CH_3_) contain sp^3^-hybridized carbon atoms. The positive impact of introducing a methyl and/or trifluoromethyl group into the structure of the considered compound can serve as a guideline for the future design of active substances based on a 1,3-butadiene core.

The introduction of one substituent to the structure of 1,3-butadiene (**1**) in compounds **2**–**5a** does not significantly improve their properties, as is the case with the presence of two-substituted compounds **2**–**5b**–**d**. What is more, the preferred positions for introducing structural modifications relative to the reference 1,3-butadiene (**1**) are definitely the terminal disubstituted position (17 potential activities in total), followed by the internal vicinal disubstituted position (12 potential activities in total) and lastly the geminal terminal disubstituted position (9 potential activities in total) (Table 4). Although diversity was introduced by incorporating different substituents, namely amino and nitro groups, in compounds **6b**–**d**, none exhibited potential activities with Pa > 0.900 (Tables S18–S20 in Supplementary Materials). This observation further reveals that the structure of 1,3-butadiene (**1**) on its own is insufficient and that the mere introduction of substituents does not remedy this limitation. A prerequisite step is the extension of the structural chain through the incorporation of sp^3^-hybridized carbon atoms, thereby reducing the overall degree of unsaturation. Consequently, while 1,3-butadiene (**1**) and its analogues (**2a**–**6d**) may serve as valuable synthetic building blocks, these compounds do not constitute molecules with promising biological potential as individual structures.

## 3. Materials and Methods

All computations were carried out with the Gaussian 16 package [147] on the Ares cluster at the CYFRONET Regional Computer Centre in Krakow. Presented calculations were performed based on the B3LYP functional [148] in combination with the 6–31G(d) basis set [149]. The applied computational level is commonly used to evaluate molecules within the DFT as well as the MEDT approach [150,151], especially for compounds containing a conjugated system in their structures [152,153,154,155,156]. Additionally, this level of theory is often combined with experimental studies, frequently affording strong correlations and accurate predictions, thus streamlining the research process from its earliest stages [157,158,159,160]. Critical structures for all considered compounds were optimized in the gas phase at a temperature of 298 K and a pressure of 1 atm. All localized stationary points were verified through vibrational analysis [161], which confirmed that all structures possessed positive definite Hessian matrices [162].

The electronic structures of 1,3-butadiene and its selected analogues were described by the Electron Localization Function (ELF) [49], the Natural Population Analysis (NPA) [68,69], and the Molecular Electrostatic Potential (MEP) [70]. The ELF analysis was carried out using the TopMod program [163]. The HOMO and LUMO energies were determined based on the optimized molecular geometries to reflect the electronic structure at the energy minimum. Global electronic properties were analyzed through reactivity descriptors based on frontier orbital energies, in accordance with Domingo’s recommendations [75,76,77]. In particular, the electronic chemical potentials μ (Equation (1)), chemical hardness η (Equation (2)), and chemical softness S (Equation (3)) were evaluated in terms of one-electron energies of frontier MOs (E_HOMO_ and E_LUMO_) using the following equations:

μ ≈ (Ε_HOMO_ + Ε_LUMO_)/2 (1)

η ≈ E_HOMO_ − E_LUMO_ (2)

S ≈ 1/η(3)

In turn, the values of global electrophilicity ω (Equation (4)) and global nucleophilicity N (Equation (5)) were calculated according to the formulas:

ω = μ^2^/η (4)

N = E_HOMO_ − E_HOMO (TCE)_ (5)

where E_HOMO (TCE)_ is the HOMO energy for tetracyanoethylene (TCE); it is the reference because it presents the lowest HOMO (E_HOMO (TCE)_ = −9.368 eV).

In parallel, electrophilic P_k_^+^ Parr functions and nucleophilic P_k_^−^ Parr functions were determined from the Atomic Spin Density (ASD) of the reagents’ radical ions [89,90]. Finally, the local electrophilicity ω_k_ (Equation (6)) and the local nucleophilicity N_k_ (Equation (7)) concentrated on atom k were calculated based on global properties and the Parr function (P_k_^+^ or P_k_^−^), according to the formulas [89,90]:

ω_k_ = P_k_^+^ · ω (6)

N_k_ = P_k_^−^ · N (7)

The physicochemical as well as pharmacokinetic properties were estimated by the SwissADME online server [106]. In order to assess drug-likeness, models based on rules of *Lipinski* et al. [107], *Ghose* et al. [108], *Veber* et al. [109], *Egan* et al. [110], and Muegge et al. [111] were applied. In turn, the analysis and prediction of potential activity spectra for the considered compounds were performed by the PASS online server [112].

In order to visualize the molecular geometries of all considered structures, including ELF, MEP, and HOMO-LUMO orbitals, as well as 3D representations of the radical anions and radical cations, the GaussView 6.0 software [164] was employed. The bioavailability radars were adopted from the SwissADME online server [106].

## 4. Conclusions and Future Prospective

In this study, a comprehensive computational analysis of the reactivity of 1,3-butadiene and its analogues was performed. Additionally, a preliminary assessment of their biological potential, drug-likeness, and likely applications based on ADME and PASS simulations was performed.

ELF analysis shows that terminal bonds concentrate populations with weaker double-bond character, while internal bonds concentrate populations stronger than single bonds. Substitution in the C1-C4 core does not qualitatively alter valence basin populations but modifies them quantitatively, most notably for nitro groups. In close proximity, nitro groups induce monosynaptic basins on nitrogen atoms, suggesting potential synthetic utility. Frontier orbital and HOMO–LUMO analyses reveal that substitution generally increases reactivity [80,81,82], particularly with nitro or amino groups, regardless of the position of substitution. Notably, 1,4-dinitro-1,3-butadiene is the least stable and most reactive, whereas 1,1-dinitro-1,3-butadiene is paradoxically the most stable and least reactive. The simultaneous presence of amino and nitro groups does not significantly alter HOMO–LUMO gaps.

Global reactivity descriptors classify 1,3-butadiene as both a moderate electrophile and nucleophile. Substitution alters these properties systematically: amino analogues act as marginal electrophiles and strong nucleophiles; methyl analogues as moderate electrophiles and strong nucleophiles; and trifluoromethyl and nitro analogues as strong electrophiles and marginal nucleophiles, with nitro compounds showing more pronounced differences. Among all compounds, 1,4-diamino-1,3-butadiene acts as a super nucleophile, and 1,4-dinitro-1,3-butadiene as a super electrophile, regardless of the second reagent [88]. Local reactivity analysis identifies terminal carbons as the most electrophilic and nucleophilic centers in 1,3-butadiene and in symmetrical disubstituted systems. In asymmetrical mono- or geminally substituted molecules, preferences vary: methyl or trifluoromethyl analogues show reactivity at the unsubstituted terminal carbon, whereas amino/nitro derivatives require individual evaluation due to strong substituent effects.

Physicochemical descriptors and ADME predictions indicate that neither 1,3-butadiene nor its studied analogues alone are independent promising drug candidates. Their limitations include low molecular weight, low molar refractivity, and excessive unsaturation. Structural modifications are thus necessary.

Two development directions can be considered. First, due to low molecular weight and high unsaturation, these molecules are suitable building blocks for synthesizing more complex structures. Substitution across different positions generates diverse electronic properties, enabling rational selection of analogues depending on the reaction partner. Second, more extensive modifications of the butadiene core can enhance drug-likeness. According to *Muegge* et al. [111], biological potential requires more than four carbons and at least one heteroatom. Thus, introducing additional carbon atoms, reducing unsaturation, and incorporating heteroatom-bearing substituents is justified. This can be achieved by chain extension and the introduction of stabilizing trifluoromethyl groups [165,166]. The inclusion of amino/nitro substituents to improve bioavailability can also be desirable [167,168].

Thus, the presented results provide a basis for employing the tested compounds as useful building blocks in the synthesis of more complex molecules. These findings may assist in the design of compounds with biological potential, not only derived from the 1,3-butadiene core but also from other conjugated systems. Additionally, PASS analysis enables identification of prospective application pathways for structures closely related to the tested molecules.

## Figures and Tables

**Table 1:** The most significant ELF valence basin populations N for molecules **1** and **2a**–**6d** (given an average number of electrons, **e**). V represents the disynaptic ELF basin of a bond, V’ corresponds to the second basin of the same bond, and V_t_ is the total electron population of the bond (V_t_ = V + V’).

	Valence Basin Populations, N [e]						
	V (C1,C2)	V’ (C1,C2)	V_t_ (C1,C2)	V (C3,C4)	V’ (C3,C4)	V_t_ (C3,C4)	V (C2,C3)
**1**	1.72	1.72	3.44	1.72	1.72	3.44	2.18
**2a**	1.77	1.81	3.58	1.74	1.77	3.51	2.23
**2b**	1.83	1.83	3.66	1.83	1.83	3.66	2.20
**2c**	1.80	1.80	3.60	1.80	1.80	3.60	2.29
**2d**	1.85	1.88	3.73	1.75	1.76	3.51	2.23
**3a**	1.70	1.79	3.49	1.69	1.78	3.47	2.21
**3b**	1.75	1.75	3.50	1.75	1.75	3.50	2.20
**3c**	1.75	1.75	3.50	1.75	1.75	3.50	2.22
**3d**	1.72	1.82	3.54	1.71	1.74	3.45	2.21
**4a**	1.74	1.74	3.48	1.70	1.70	3.40	2.21
**4b**	1.68	1.78	3.46	1.68	1.78	3.46	2.20
**4c**	1.75	1.75	3.50	1.75	1.75	3.50	2.20
**4d**	1.78	1.80	3.58	1.66	1.70	3.36	2.23
**5a**	1.76	1.76	3.52	1.68	1.68	3.36	2.22
**5b**	1.73	1.73	3.46	1.73	1.73	3.46	2.23
**5c**	1.74	1.84	3.58	1.74	1.84	3.58	2.30
**5d**	1.89	1.89	3.78	1.63	1.63	3.26	2.27
**6b**	1.73	1.73	3.46	1.76	1.76	3.52	2.24
**6c**	1.78	1.78	3.56	1.81	1.81	3.62	2.29
**6d**	1.89	1.90	3.79	1.66	1.75	3.41	2.20

**Table 2:** The main reactivity indices for molecules **1** and **2a**–**6d** (given in electronvolts, eV).

	Main Reactivity Descriptors [eV]		
	μ	η	S
**1**	−3.42	5.62	0.18
**2a**	−2.65	5.11	0.20
**2b**	−1.96	4.86	0.21
**2c**	−3.17	5.50	0.18
**2d**	−2.47	4.99	0.20
**3a**	−3.33	5.35	0.19
**3b**	−3.19	5.42	0.18
**3c**	−3.30	5.64	0.18
**3d**	−3.32	5.34	0.19
**4a**	−4.45	5.49	0.18
**4b**	−5.11	5.43	0.18
**4c**	−4.54	5.69	0.18
**4d**	−5.05	5.30	0.19
**5a**	−5.25	4.67	0.21
**5b**	−6.11	4.40	0.23
**5c**	−5.23	5.00	0.20
**5d**	−5.51	6.01	0.17
**6b**	−4.84	4.52	0.22
**6c**	−4.38	4.99	0.20
**6d**	−4.30	4.70	0.21

**Table 3:** Drug-likeness parameters for molecules **1** and **2a**–**6d**, determined using the SwissADME [113].

		1	2a	2b	2c	2d	3a	3b	3c	3d	4a	4b	4c	4d	5a	5b	5c	5d	6b	6c	6d
**Physchem. properties**	**MW [g/mol]**	54.09	69.11	84.12	84.12	84.12	68.12	82.14	82.14	82.14	122.09	190.09	190.09	190.09	99.09	144.09	144.09	144.09	114.10	114.10	114.10
	**#heavy atoms**	4	5	6	6	6	5	6	6	6	8	12	12	12	7	10	10	10	8	8	8
	**#arom. heavy atoms**	0	0	0	0	0	0	0	0	0	0	0	0	0	0	0	0	0	0	0	0
	**#rotatable bonds**	1	1	1	1	1	1	1	1	1	2	3	3	3	2	3	3	3	2	2	2
	**#H-bond acceptors**	0	0	0	0	0	0	0	0	0	3	6	6	6	2	4	4	4	2	2	2
	**#H-bond donors**	0	1	2	2	2	0	0	0	0	0	0	0	0	0	0	0	0	1	1	1
	**Molar refractivity**	20.39	23.10	25.81	25.81	25.81	25.20	30.01	30.01	30.01	25.39	30.39	30.39	30.39	28.50	36.60	36.60	36.60	31.21	31.21	31.21
	**TPSA [Å^2^]**	0.00	26.02	52.04	52.04	52.04	0.00	0.00	0.00	0.00	0.00	0.00	0.00	0.00	45.82	91.64	91.64	91.64	71.84	71.84	71.84
**Lipophilicity**	**Log P_o/w_ (iLOGP)**	1.68	1.29	0.93	0.94	1.11	1.91	2.14	2.03	2.13	1.83	2.13	2.02	2.05	1.21	−2.36	0.65	0.34	0.73	1.52	0.92
	**Log P_o/w_ (XLOGP3)**	1.99	0.82	−0.21	0.07	0.34	2.40	2.80	3.09	2.70	2.60	3.35	4.13	3.74	1.33	0.81	1.09	1.36	0.30	2.09	0.85
	**Log P_o/w_ (WLOGP)**	1.36	0.64	−0.07	−0.07	−0.07	1.75	2.14	2.14	2.14	3.55	5.74	5.74	5.74	1.49	1.61	1.61	1.61	0.77	1.88	0.77
	**Log P_o/w_ (MLOGP)**	1.56	0.49	−0.43	−0.43	−0.03	1.97	2.35	2.35	2.35	2.53	3.36	3.36	3.36	−0.22	−0.78	−1.59	−0.78	−1.07	0.19	−0.25
	**Log P_o/w_ (SILICOS-IT)**	0.88	0.01	−0.86	−0.82	−0.84	1.15	1.42	1.45	1.44	2.07	3.16	3.20	3.18	−0.73	−2.34	−2.31	−2.33	−1.60	−0.51	−1.58
	**Consensus Log P_o/w_**	1.49	0.65	−0.13	−0.06	0.10	1.84	2.17	2.21	2.15	2.52	3.55	3.69	3.61	0.62	−0.61	−0.11	0.04	−0.17	1.03	0.14
**Water solubility**	**Log S (ESOL)**	−1.36	−0.72	−0.16	−0.34	−0.51	−1.71	−2.05	−2.23	−1.98	−2.10	−2.93	−3.42	−3.18	−1.16	−1.05	−1.22	−1.39	−0.60	−1.73	−0.95
	**solubility [mg/mL]**	2.34	13.2	57.8	38.5	26.0	1.33	0.737	0.484	0.854	0.963	0.223	0.072	0.127	6.85	13.0	8.64	5.84	28.4	2.13	12.8
	**Log S (Ali)**	−1.62	−0.95	−0.43	−0.72	−1.00	−2.04	−2.46	−2.76	−2.35	−2.25	−3.03	−3.84	−3.43	−1.89	−2.32	−2.61	−2.89	−1.37	−2.68	−1.94
	**solubility [mg/mL]**	1.31	7.18	31.5	16.2	8.47	0.619	0.287	0.144	0.364	0.688	0.178	0.028	0.070	1.27	0.696	0.356	0.187	4.85	0.235	1.30
	**Log S (SILICOS-IT)**	−0.58	0.09	0.77	0.06	0.41	−0.65	−0.72	−1.43	−1.08	−1.26	−1.83	−2.55	−2.19	−0.20	0.24	−0.47	−0.11	0.50	−0.96	0.14
	**solubility [mg/mL]**	14.3	85.0	293	95.7	217	15.1	15.7	3.04	6.90	6.77	2.78	0.538	1.22	63.2	253	49.0	112	358	12.4	158
**Pharmacokinetics**	**CYP1A2 inhibitor**	No	No	No	No	No	No	No	No	No	No	No	No	No	No	No	No	No	No	No	No
	**CYP2C19 inhibitor**	No	No	No	No	No	No	No	No	No	No	No	No	No	No	No	No	No	No	No	No
	**CYP2C9 inhibitor**	No	No	No	No	No	No	No	No	No	No	No	No	No	No	No	No	No	No	No	No
	**CYP2D6 inhibitor**	No	No	No	No	No	No	No	No	No	No	No	No	No	No	No	No	No	No	No	No
	**CYP3A4 inhibitor**	No	No	No	No	No	No	No	No	No	No	No	No	No	No	No	No	No	No	No	No
	**IG absorption**	High	High	High	High	High	High	High	High	High	High	High	High	High	High	High	High	High	High	High	High
	**BBB permeant**	No	No	No	No	No	No	No	No	No	No	No	No	No	No	No	No	No	No	No	No
**Druglikeness**	***Lipinski* et al. (Pfizer)** [107]	√	√	√	√	√	√	√	√	√	√	√	√	√	√	√	√	√	√	√	√
	***Ghose* et al. (Amgen)** [108]	×	×	×	×	×	×	×	×	×	×	×	×	×	×	×	×	×	×	×	×
	***Veber* et al. (GSK)** [109]	√	√	√	√	√	√	√	√	√	√	√	√	√	√	√	√	√	√	√	√
	***Egan* et al. (Pharmacia)** [110]	√	√	√	√	√	√	√	√	√	√	√	√	√	√	√	√	√	√	√	√
	***Muegge* et al. (Bayer)** [111]	×	×	×	×	×	×	×	×	×	×	×	×	×	×	×	×	×	×	×	×

**Table 4:** The PASS prediction [112] of the main potential activities for molecules **1** and **2a**–**5d**. The results are expressed for a molecule’s probability of being active, Pa > 0.900.

	1	2a	2b	2c	2d	3a	3b	3c	3d	4a	4b	4c	4d	5a	5b	5c	5d	Sum.
Aspulvinone dimethylallyltransferase inhibitor	0.940			0.913		0.916	0.917	0.965	0.936			0.942				0.913		**8**
Antieczematic	0.918																	**1**
Fatty-acyl-CoA synthase inhibitor	0.917					0.935	0.919	0.903	0.914									**5**
Beta-adrenergic receptor kinase inhibitor	0.902								0.911									**2**
G-protein-coupled receptor kinase inhibitor	0.902								0.911									**2**
Arachidonate-CoA ligase inhibitor			0.917															**1**
Phobic disorder treatment			0.908	0.904														**2**
NADPH peroxidase inhibitor			0.900	0.903														**2**
Integrin alphaVbeta3 antagonist				0.909														**1**
Apoptosis agonist									0.909									**1**
Mucomembranous protector									0.908									**1**
Ubiquinol-cytochrome-c reductase inhibitor							0.934											**1**
Phosphatidylcholine-retinol O-acyltransferase inhibitor							0.910				0.928							**2**
Cl--transporting ATPase inhibitor								0.918										**1**
Cardiotonic								0.902										**1**
Antineoplastic (breast cancer)								0.938		0.938	0.936						0.934	**4**
Antineoplastic (lung cancer)								0.929		0.929	0.928							**3**
Epidermal growth factor antagonist											0.980							**1**
Growth factor antagonist											0.946							**1**
Epidermal growth factor receptor kinase inhibitor											0.936							**1**
Antineoplastic (colorectal cancer)											0.921							**1**
Vitamin D-like												0.919	0.966					**2**
Atherosclerosis treatment												0.927	0.949					**2**
Saccharopepsin inhibitor															0.912			**1**
Acrocylindropepsin inhibitor															0.912			**1**
Chymosin inhibitor															0.912			**1**
**Sum.**	**5**	**0**	**3**	**4**	**0**	**2**	**4**	**6**	**6**	**2**	**7**	**2**	**2**	**0**	**3**	**0**	**1**	

## Data Availability

The data presented in this study are available on request from the corresponding author.

## Supplementary Materials

The following supporting information can be downloaded at: https://www.mdpi.com/article/10.3390/ijms26188983/s1.
