# Supplementary material for: In Silico Study About Substituent Effects, Electronic Properties, and the Biological Potential of 1,3-Butadiene Analogues
Source: Int J Mol Sci. 2025 Sep 15;26(18):8983. doi: 10.3390/ijms26188983 (PMC12469405; doi:10.3390/ijms26188983)
Supplement: Supplementary file 1 [file ijms-26-08983-s001.zip › ijms-3849910-supplementary.pdf]

SUPPLEMENTARY MATERIALS

In-silico Study About Substituent Effect, Electronic Properties and Biological Potential of 1,3-Butadiene Analogues

Karolina Kula [1,*] and Emilia Kuś [1]

[1] Cracow University of Technology, Faculty of Chemical Engineering and Technology, Warszawska 24, 31-155 Cracow, Poland

* Correspondence adress: karolina.kula@pk.edu.pl (K.K)

INDEX

Figure S1. The structure of all tested compounds (**1** and **2a-6d**)……………………………………………………3

Table S1-S20. Prediction of the selected activity (Pa > 0.7) using PASS software…………………………………..4

Table S21-S40. Cartesian coordinates according to B3LYP/6-31G(d) level theory in gas phase ………………51

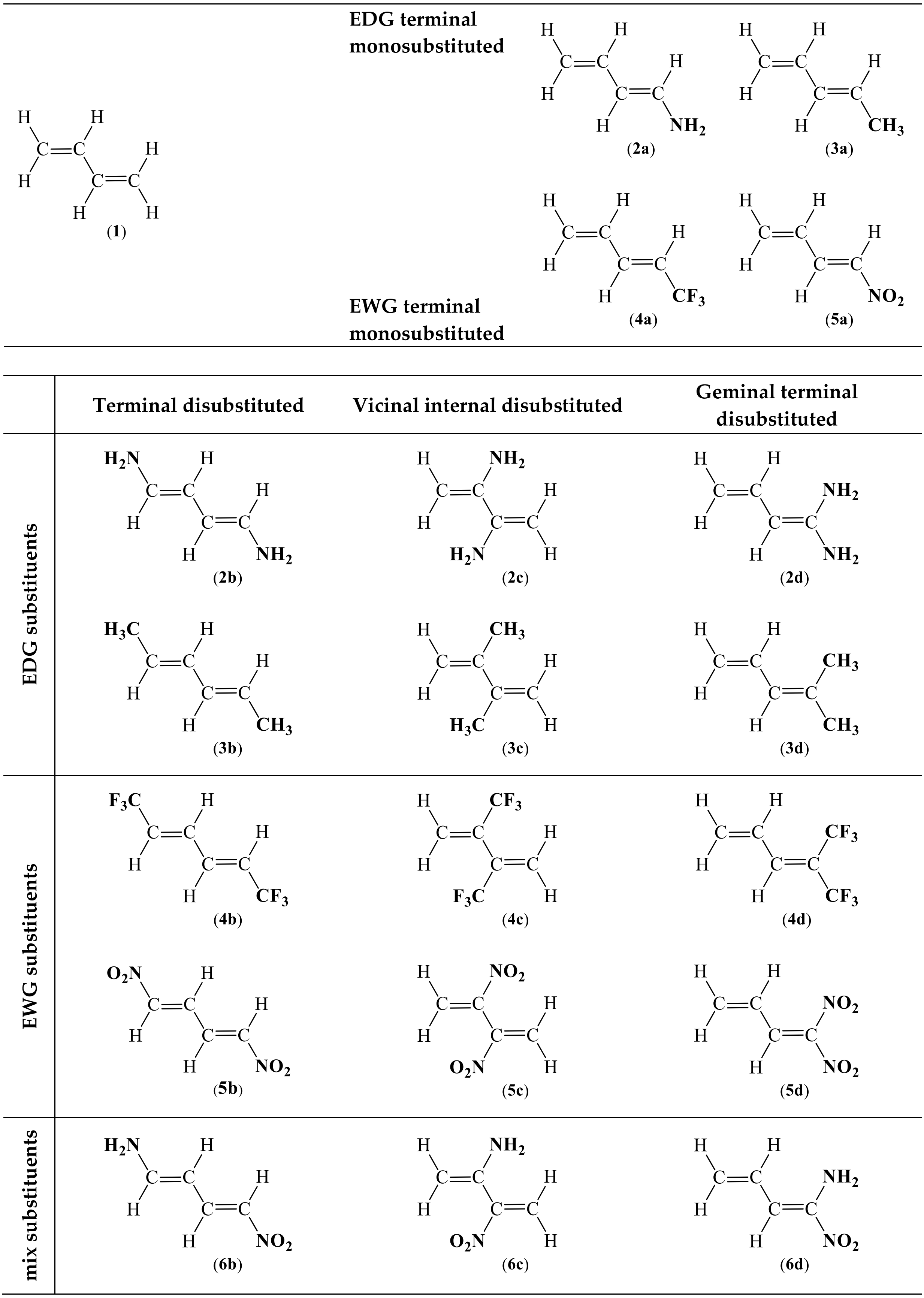

Figure S1. The structure of all tested compounds (**1** and **2a-6d**).

Table S1. Prediction of the selected activity (Pa > 0.7) of molecule (**1**) using PASS software. The results are expressed as probability to be active (Pa) or inactive (Pi).

	**Pa**	**Pi**
Aspulvinone dimethylallyltransferase inhibitor	0.940	0.004
Fatty-acyl-CoA synthase inhibitor	0.917	0.002
Antieczematic	0.918	0.004
Beta-adrenergic receptor kinase inhibitor	0.902	0.004
G-protein-coupled receptor kinase inhibitor	0.902	0.004
Chloride peroxidase inhibitor	0.895	0.002
Cl--transporting ATPase inhibitor	0.893	0.003
Gluconate 2-dehydrogenase (acceptor) inhibitor	0.891	0.004
Carminative	0.887	0.002
Antineoplastic (breast cancer)	0.885	0.004
Mucomembranous protector	0.881	0.005
Testosterone 17beta-dehydrogenase (NADP+) inhibitor	0.877	0.009
Chlordecone reductase inhibitor	0.874	0.008
Antineoplastic	0.871	0.005
Sugar-phosphatase inhibitor	0.873	0.007
Phosphoenolpyruvate-protein phosphotransferase inhibitor	0.867	0.002
NADPH peroxidase inhibitor	0.867	0.005
Apoptosis agonist	0.865	0.005
NADH kinase inhibitor	0.862	0.003
Feruloyl esterase inhibitor	0.865	0.006
Phobic disorders treatment	0.870	0.011
Ubiquinol-cytochrome-c reductase inhibitor	0.868	0.012
Complement factor D inhibitor	0.858	0.004
Glycosylphosphatidylinositol phospholipase D inhibitor	0.857	0.005
Glutamyl endopeptidase II inhibitor	0.856	0.005
Phosphatidylcholine-retinol O-acyltransferase inhibitor	0.855	0.004
Chymosin inhibitor	0.859	0.010
Acrocylindropepsin inhibitor	0.859	0.010
Saccharopepsin inhibitor	0.859	0.010
Antineoplastic (pancreatic cancer)	0.846	0.002
Ribulose-phosphate 3-epimerase inhibitor	0.844	0.004
5-O-(4-coumaroyl)-D-quinate 3'-monooxygenase inhibitor	0.844	0.004
Nicotinic alpha6beta3beta4alpha5 receptor antagonist	0.843	0.005
CYP2J substrate	0.846	0.011
Dehydro-L-gulonate decarboxylase inhibitor	0.839	0.005
All-trans-retinyl-palmitate hydrolase inhibitor	0.835	0.004
Antineoplastic (ovarian cancer)	0.833	0.003
Carboxypeptidase Taq inhibitor	0.834	0.005
Antineoplastic (lung cancer)	0.830	0.004
Bisphosphoglycerate phosphatase inhibitor	0.830	0.005
UDP-N-acetylglucosamine 4-epimerase inhibitor	0.829	0.004
Pullulanase inhibitor	0.824	0.006
2-Hydroxymuconate-semialdehyde hydrolase inhibitor	0.822	0.005
Aldehyde dehydrogenase (pyrroloquinoline-quinone) inhibitor	0.819	0.004

Nicotinic alpha2beta2 receptor antagonist	0.822	0.007
GST A substrate	0.820	0.008
Fusarinine-C ornithinesterase inhibitor	0.816	0.005
CYP2J2 substrate	0.818	0.010
Alkylacetylglycerophosphatase inhibitor	0.815	0.008
Phthalate 4,5-dioxygenase inhibitor	0.811	0.004
2,3-Oxidosqualene-lanosterol cyclase inhibitor	0.807	0.001
6-Pyruvoyltetrahydropterin synthase inhibitor	0.809	0.003
Creatininase inhibitor	0.811	0.005
IgA-specific serine endopeptidase inhibitor	0.811	0.005
Membrane permeability inhibitor	0.814	0.008
Glucan endo-1,6-beta-glucosidase inhibitor	0.810	0.007
4-Nitrophenol 2-monooxygenase inhibitor	0.807	0.004
Acylcarnitine hydrolase inhibitor	0.814	0.012
Alkenylglycerophosphocholine hydrolase inhibitor	0.818	0.015
Electron-transferring-flavoprotein dehydrogenase inhibitor	0.806	0.004
Polyamine-transporting ATPase inhibitor	0.807	0.005
Phospholipid-translocating ATPase inhibitor	0.805	0.005
Membrane integrity agonist	0.829	0.029
L-glutamate oxidase inhibitor	0.804	0.004
Chenodeoxycholoyltaurine hydrolase inhibitor	0.803	0.004
Allyl-alcohol dehydrogenase inhibitor	0.801	0.004
Glutathione thiolesterase inhibitor	0.803	0.007
Gluconate 5-dehydrogenase inhibitor	0.799	0.004
NADPH-cytochrome-c2 reductase inhibitor	0.798	0.006
Pterin deaminase inhibitor	0.794	0.005
Crotonoyl-[acyl-carrier-protein] hydratase inhibitor	0.793	0.003
Cutinase inhibitor	0.794	0.005
Lysostaphin inhibitor	0.791	0.004
3-Hydroxybenzoate 6-monooxygenase inhibitor	0.792	0.005
Acetylesterase inhibitor	0.792	0.005
Glucan 1,4-alpha-maltotriohydrolase inhibitor	0.790	0.004
Arginine 2-monooxygenase inhibitor	0.795	0.009
Taurine dehydrogenase inhibitor	0.798	0.013
Dimethylargininase inhibitor	0.791	0.007
Venombin AB inhibitor	0.790	0.007
Amine dehydrogenase inhibitor	0.788	0.004
Methylamine-glutamate N-methyltransferase inhibitor	0.783	0.005
Pro-opiomelanocortin converting enzyme inhibitor	0.792	0.015
Thioredoxin inhibitor	0.780	0.004
Fragilysin inhibitor	0.785	0.009
CYP2C12 substrate	0.808	0.034
Glutamine-phenylpyruvate transaminase inhibitor	0.778	0.005
Polyneuridine-aldehyde esterase inhibitor	0.776	0.003
Arylacetonitrilase inhibitor	0.784	0.011
tRNA-pseudouridine synthase I inhibitor	0.775	0.004
Cyclohexyl-isocyanide hydratase inhibitor	0.772	0.004
Alkane 1-monooxygenase inhibitor	0.773	0.008

5 Hydroxytryptamine release stimulant	0.780	0.016
Omptin inhibitor	0.774	0.010
2-Haloacid dehalogenase inhibitor	0.767	0.003
Polyporopepsin inhibitor	0.786	0.022
Macrophage colony stimulating factor agonist	0.769	0.006
Ferredoxin-NAD+ reductase inhibitor	0.767	0.004
Naphthalene 1,2-dioxygenase inhibitor	0.767	0.004
S-alkylcysteine lyase inhibitor	0.767	0.005
Limulus clotting factor B inhibitor	0.768	0.006
Glucan 1,4-alpha-maltotetraohydrolase inhibitor	0.766	0.004
Exoribonuclease II inhibitor	0.771	0.010
Glucose oxidase inhibitor	0.774	0.015
N-Acyl-D-aspartate deacylase inhibitor	0.763	0.004
Shikimate O-hydroxycinnamoyltransferase inhibitor	0.763	0.004
Poly(alpha-L-guluronate) lyase inhibitor	0.764	0.005
Steroid N-acetylglucosaminyltransferase inhibitor	0.762	0.004
Aspartate-phenylpyruvate transaminase inhibitor	0.763	0.005
N-formylmethionyl-peptidase inhibitor	0.761	0.004
(S)-6-hydroxynicotine oxidase inhibitor	0.759	0.004
Methylenetetrahydrofolate reductase (NADPH) inhibitor	0.775	0.022
Transcription factor NF kappa B stimulant	0.755	0.003
Transcription factor stimulant	0.755	0.003
JAK2 expression inhibitor	0.761	0.011
N-benzyloxycarbonylglycine hydrolase inhibitor	0.756	0.007
GABA aminotransferase inhibitor	0.751	0.004
4-Hydroxyglutamate transaminase inhibitor	0.750	0.003
Arylmalonate decarboxylase inhibitor	0.750	0.004
Pseudolysin inhibitor	0.756	0.011
Sphinganine kinase inhibitor	0.760	0.018
Gamma-guanidinobutyraldehyde dehydrogenase inhibitor	0.747	0.005
CYP2F1 substrate	0.746	0.004
CYP2A8 substrate	0.745	0.005
Taurocyamine kinase inhibitor	0.743	0.004
Opheline kinase inhibitor	0.743	0.004
Phenol O-methyltransferase inhibitor	0.743	0.005
Aminocarboxymuconate-semialdehyde decarboxylase inhibitor	0.739	0.004
2-Hydroxyquinoline 8-monooxygenase inhibitor	0.744	0.010
ADP-thymidine kinase inhibitor	0.745	0.011
Xylan endo-1,3-beta-xylosidase inhibitor	0.738	0.006
N-acetylneuraminate 7-O(or 9-O)-acetyltransferase inhibitor	0.742	0.010
Antiseborrheic	0.759	0.027
Glyoxylate reductase inhibitor	0.737	0.004
Pediculicide	0.733	0.002
Tryptophanamidase inhibitor	0.735	0.005
27-Hydroxycholesterol 7alpha-monooxygenase inhibitor	0.738	0.009
Long-chain-aldehyde dehydrogenase inhibitor	0.732	0.005
Mannan endo-1,4-beta-mannosidase inhibitor	0.731	0.004
2-Hydroxy-3-oxoadipate synthase inhibitor	0.730	0.003

Aspartate-ammonia ligase inhibitor	0.732	0.006
Horrilysin inhibitor	0.730	0.004
Formaldehyde transketolase inhibitor	0.734	0.008
Albendazole monooxygenase inhibitor	0.728	0.004
Aminobutyraldehyde dehydrogenase inhibitor	0.728	0.005
Hydrogen dehydrogenase inhibitor	0.729	0.007
Ovulation inhibitor	0.726	0.005
Lysine 2,3-aminomutase inhibitor	0.733	0.012
CYP2E1 inhibitor	0.725	0.004
Methanol dehydrogenase inhibitor	0.724	0.004
Threonine aldolase inhibitor	0.726	0.008
Manganese peroxidase inhibitor	0.727	0.010
(R)-6-hydroxynicotine oxidase inhibitor	0.723	0.006
Poly(beta-D-mannuronate) lyase inhibitor	0.721	0.005
Arylalkyl acylamidase inhibitor	0.721	0.005
Antineoplastic (lymphoma)	0.719	0.004
N-acylmannosamine kinase inhibitor	0.720	0.005
Deoxyribose-phosphate aldolase inhibitor	0.715	0.002
1,4-Lactonase inhibitor	0.720	0.008
Sulfite reductase inhibitor	0.719	0.007
Snapalysin inhibitor	0.716	0.004
IgA-specific metalloendopeptidase inhibitor	0.717	0.007
CYP2B5 substrate	0.717	0.008
Corticosteroid side-chain-isomerase inhibitor	0.716	0.008
Mucinaminylserine mucinaminidase inhibitor	0.718	0.010
GST P substrate	0.710	0.004
Spermidine dehydrogenase inhibitor	0.713	0.008
GABA C receptor agonist	0.707	0.003
N-hydroxyarylamine O-acetyltransferase inhibitor	0.708	0.005
Ecdysone 20-monooxygenase inhibitor	0.711	0.008
Trimethylamine-oxide aldolase inhibitor	0.710	0.007
Glyoxylate oxidase inhibitor	0.703	0.004
CYP2D16 substrate	0.703	0.005
Limulus clotting factor C inhibitor	0.707	0.010
Fibrolase inhibitor	0.704	0.007
Fructan beta-fructosidase inhibitor	0.702	0.005
Carbon-monoxide dehydrogenase inhibitor	0.701	0.004
Fucosterol-epoxide lyase inhibitor	0.703	0.015
Protein-disulfide reductase (glutathione) inhibitor	0.702	0.020

Table S2. Prediction of the selected activity (Pa > 0.7) of molecule (**2a**) using PASS software. The results are expressed as probability to be active (Pa) or inactive (Pi).

	**Pa**	**Pi**
Fatty-acyl-CoA synthase inhibitor	0.886	0.003
Beta-adrenergic receptor kinase inhibitor	0.878	0.006
G-protein-coupled receptor kinase inhibitor	0.878	0.006
Aspulvinone dimethylallyltransferase inhibitor	0.875	0.014
Chloride peroxidase inhibitor	0.850	0.003
Phobic disorders treatment	0.849	0.017
NADPH peroxidase inhibitor	0.834	0.008
Mucomembranous protector	0.831	0.012
Acrocylindropepsin inhibitor	0.827	0.015
Chymosin inhibitor	0.827	0.015
Saccharopepsin inhibitor	0.827	0.015
Complement factor D inhibitor	0.814	0.005
Pro-opiomelanocortin converting enzyme inhibitor	0.815	0.012
Arachidonate-CoA ligase inhibitor	0.795	0.000
Cl--transporting ATPase inhibitor	0.790	0.007
Glutamine-phenylpyruvate transaminase inhibitor	0.780	0.005
Polyamine-transporting ATPase inhibitor	0.775	0.006
CYP2E1 substrate	0.764	0.005
Acylcarnitine hydrolase inhibitor	0.775	0.016
Fusarinine-C ornithinesterase inhibitor	0.769	0.011
CYP2E substrate	0.762	0.005
Nicotinic alpha6beta3beta4alpha5 receptor antagonist	0.770	0.017
Carminative	0.758	0.005
Testosterone 17beta-dehydrogenase (NADP+) inhibitor	0.781	0.030
Ubiquinol-cytochrome-c reductase inhibitor	0.786	0.038
Glucose oxidase inhibitor	0.762	0.016
Antieczematic	0.768	0.026
Albendazole monooxygenase inhibitor	0.745	0.003
Venombin AB inhibitor	0.747	0.011
Omptin inhibitor	0.749	0.013
Glycosylphosphatidylinositol phospholipase D inhibitor	0.756	0.022
Pterin deaminase inhibitor	0.740	0.008
NADPH-cytochrome-c2 reductase inhibitor	0.742	0.012
Limulus clotting factor B inhibitor	0.737	0.009
Macrophage colony stimulating factor agonist	0.737	0.009
UDP-N-acetylglucosamine 4-epimerase inhibitor	0.738	0.012
Dimethylargininase inhibitor	0.736	0.011
Glutamyl endopeptidase II inhibitor	0.743	0.019
GST A substrate	0.741	0.017
Sugar-phosphatase inhibitor	0.748	0.025
S-alkylcysteine lyase inhibitor	0.729	0.006
Pseudolysin inhibitor	0.730	0.015
Phthalate 4,5-dioxygenase inhibitor	0.725	0.011
Membrane permeability inhibitor	0.738	0.025

NADH kinase inhibitor	0.716	0.006
Fibrolase inhibitor	0.715	0.006
Phosphoenolpyruvate-protein phosphotransferase inhibitor	0.711	0.003
Chlordecone reductase inhibitor	0.740	0.032
Exoribonuclease II inhibitor	0.719	0.015
Fragilysin inhibitor	0.719	0.015
Carboxypeptidase Taq inhibitor	0.716	0.017
CYP2J substrate	0.736	0.038
Arginine 2-monooxygenase inhibitor	0.715	0.018
Nicotinic alpha2beta2 receptor antagonist	0.720	0.023
Hydrogen dehydrogenase inhibitor	0.706	0.009
Threonine aldolase inhibitor	0.706	0.009
Dehydro-L-gulonate decarboxylase inhibitor	0.716	0.020
Polyporopepsin inhibitor	0.720	0.035
5-O-(4-coumaroyl)-D-quinate 3'-monooxygenase inhibitor	0.706	0.022
Feruloyl esterase inhibitor	0.702	0.021
Antiseborrheic	0.711	0.036
Gluconate 2-dehydrogenase (acceptor) inhibitor	0.719	0.046
CDP-glycerol glycerophosphotransferase inhibitor	0.710	0.051

Table S3. Prediction of the selected activity (Pa > 0.7) of molecule (**2b**) using PASS software. The results are expressed as probability to be active (Pa) or inactive (Pi).

	**Pa**	**Pi**
Arachidonate-CoA ligase inhibitor	0.917	0.000
Phobic disorders treatment	0.908	0.005
NADPH peroxidase inhibitor	0.900	0.004
Acrocylindropepsin inhibitor	0.892	0.006
Chymosin inhibitor	0.892	0.006
Saccharopepsin inhibitor	0.892	0.006
Complement factor D inhibitor	0.886	0.003
Pro-opiomelanocortin converting enzyme inhibitor	0.885	0.005
Arylacetonitrilase inhibitor	0.869	0.005
Aspulvinone dimethylallyltransferase inhibitor	0.877	0.013
Ubiquinol-cytochrome-c reductase inhibitor	0.873	0.011
Glutamine-phenylpyruvate transaminase inhibitor	0.863	0.003
Glucose oxidase inhibitor	0.863	0.006
Fatty-acyl-CoA synthase inhibitor	0.859	0.003
Polyamine-transporting ATPase inhibitor	0.859	0.003
Acylcarnitine hydrolase inhibitor	0.859	0.007
Nicotinic alpha6beta3beta4alpha5 receptor antagonist	0.857	0.005
Membrane integrity agonist	0.870	0.019
Testosterone 17beta-dehydrogenase (NADP+) inhibitor	0.861	0.012
Chloride peroxidase inhibitor	0.851	0.003
Fusarinine-C ornithinesterase inhibitor	0.850	0.004
Venombin AB inhibitor	0.848	0.004
Glycosylphosphatidylinositol phospholipase D inhibitor	0.848	0.006
Fragilysin inhibitor	0.840	0.005
Albendazole monooxygenase inhibitor	0.837	0.002
Pterin deaminase inhibitor	0.838	0.004
Feruloyl esterase inhibitor	0.841	0.008
CDP-glycerol glycerophosphotransferase inhibitor	0.852	0.019
UDP-N-acetylglucosamine 4-epimerase inhibitor	0.837	0.004
Dimethylargininase inhibitor	0.837	0.004
NADPH-cytochrome-c2 reductase inhibitor	0.837	0.004
Nicotinic alpha2beta2 receptor antagonist	0.837	0.006
S-alkylcysteine lyase inhibitor	0.834	0.003
Antiseborrheic	0.841	0.012
Arginine 2-monooxygenase inhibitor	0.834	0.006
Sugar-phosphatase inhibitor	0.839	0.011
Glutamyl endopeptidase II inhibitor	0.834	0.006
Omptin inhibitor	0.831	0.005
Limulus clotting factor B inhibitor	0.825	0.004
Methylenetetrahydrofolate reductase (NADPH) inhibitor	0.835	0.014
Dehydro-L-gulonate decarboxylase inhibitor	0.827	0.007
Threonine aldolase inhibitor	0.824	0.004
Carboxypeptidase Taq inhibitor	0.825	0.005
Pseudolysin inhibitor	0.824	0.005

Polyporopepsin inhibitor	0.833	0.014
N-benzyloxycarbonylglycine hydrolase inhibitor	0.823	0.004
Exoribonuclease II inhibitor	0.822	0.006
Macrophage colony stimulating factor agonist	0.820	0.004
Phthalate 4,5-dioxygenase inhibitor	0.819	0.004
Sulfite reductase inhibitor	0.817	0.004
CYP2J substrate	0.826	0.015
Fibrolase inhibitor	0.810	0.003
Phosphatidylcholine-retinol O-acyltransferase inhibitor	0.811	0.005
Creatininase inhibitor	0.806	0.005
Sphinganine kinase inhibitor	0.812	0.012
5-O-(4-coumaroyl)-D-quinate 3'-monooxygenase inhibitor	0.806	0.007
Superoxide dismutase inhibitor	0.805	0.006
Ribulose-phosphate 3-epimerase inhibitor	0.803	0.007
Aspartate-ammonia ligase inhibitor	0.800	0.004
Manganese peroxidase inhibitor	0.798	0.005
Cl--transporting ATPase inhibitor	0.798	0.006
Pullulanase inhibitor	0.800	0.008
Hydrogen dehydrogenase inhibitor	0.796	0.004
GABA C receptor agonist	0.793	0.002
Thioredoxin inhibitor	0.793	0.004
Chenodeoxycholoyltaurine hydrolase inhibitor	0.792	0.004
Sulfite oxidase inhibitor	0.790	0.004
Formaldehyde transketolase inhibitor	0.789	0.005
Limulus clotting factor C inhibitor	0.790	0.005
Electron-transferring-flavoprotein dehydrogenase inhibitor	0.789	0.004
Taurine dehydrogenase inhibitor	0.797	0.013
Styrene-oxide isomerase inhibitor	0.786	0.003
CYP2J2 substrate	0.796	0.014
Glucan endo-1,6-beta-glucosidase inhibitor	0.791	0.009
Mucomembranous protector	0.800	0.018
Fructan beta-fructosidase inhibitor	0.784	0.004
All-trans-retinyl-palmitate hydrolase inhibitor	0.785	0.005
Glutathione thiolesterase inhibitor	0.788	0.008
Lysine 2,3-aminomutase inhibitor	0.784	0.007
L-glutamate oxidase inhibitor	0.781	0.005
Lysostaphin inhibitor	0.780	0.004
G-protein-coupled receptor kinase inhibitor	0.789	0.014
Beta-adrenergic receptor kinase inhibitor	0.789	0.014
Histidine N-acetyltransferase inhibitor	0.778	0.004
Allyl-alcohol dehydrogenase inhibitor	0.776	0.004
Mucinaminylserine mucinaminidase inhibitor	0.777	0.006
Bisphosphoglycerate phosphatase inhibitor	0.778	0.008
Phosphatidylserine decarboxylase inhibitor	0.774	0.005
Phospholipid-translocating ATPase inhibitor	0.774	0.006
Fucosterol-epoxide lyase inhibitor	0.777	0.009
Peptide-N4-(N-acetyl-beta-glucosaminyl)asparagine amidase inhibitor	0.771	0.005
D-alanine 2-hydroxymethyltransferase inhibitor	0.769	0.003

Mannotetraose 2-alpha-N-acetylglucosaminyltransferase inhibitor	0.781	0.016
Endopeptidase So inhibitor	0.768	0.004
IgA-specific serine endopeptidase inhibitor	0.771	0.008
Meprin B inhibitor	0.765	0.003
Aspartate-phenylpyruvate transaminase inhibitor	0.767	0.005
Polyneuridine-aldehyde esterase inhibitor	0.763	0.003
tRNA-pseudouridine synthase I inhibitor	0.763	0.004
2-Hydroxymuconate-semialdehyde hydrolase inhibitor	0.766	0.009
Alkylacetylglycerophosphatase inhibitor	0.764	0.013
Ferredoxin-NAD+ reductase inhibitor	0.755	0.004
Naphthalene 1,2-dioxygenase inhibitor	0.755	0.004
Nucleoside oxidase (H2O2-forming) inhibitor	0.758	0.008
Leucolysin inhibitor	0.752	0.004
Methylamine-glutamate N-methyltransferase inhibitor	0.755	0.007
Glycine dehydrogenase (decarboxylating) inhibitor	0.749	0.003
Phenylalanine(histidine) transaminase inhibitor	0.749	0.003
Acetylesterase inhibitor	0.753	0.008
(S)-6-hydroxynicotine oxidase inhibitor	0.749	0.004
GST A substrate	0.759	0.015
Protein-disulfide reductase (glutathione) inhibitor	0.756	0.013
Gluconate 5-dehydrogenase inhibitor	0.749	0.007
Alkane 1-monooxygenase inhibitor	0.753	0.010
Methylumbelliferyl-acetate deacetylase inhibitor	0.747	0.006
ADP-thymidine kinase inhibitor	0.749	0.010
Carnitinamidase inhibitor	0.743	0.005
Glucan 1,4-alpha-maltotriohydrolase inhibitor	0.742	0.006
Acetylornithine deacetylase inhibitor	0.740	0.004
N-acetylneuraminate 7-O(or 9-O)-acetyltransferase inhibitor	0.746	0.010
Gamma-guanidinobutyraldehyde dehydrogenase inhibitor	0.741	0.005
3-Hydroxybenzoate 6-monooxygenase inhibitor	0.741	0.008
Gamma-D-Glutamyl-meso-diaminopimelate peptidase inhibitor	0.735	0.003
Alkenylglycerophosphocholine hydrolase inhibitor	0.756	0.024
D-xylulose reductase inhibitor	0.735	0.004
Taurine-2-oxoglutarate transaminase inhibitor	0.732	0.003
2-Dehydropantoate 2-reductase inhibitor	0.741	0.012
Crotonoyl-[acyl-carrier-protein] hydratase inhibitor	0.733	0.004
Arylalkyl acylamidase inhibitor	0.731	0.005
2-Hydroxyquinoline 8-monooxygenase inhibitor	0.733	0.011
Shikimate O-hydroxycinnamoyltransferase inhibitor	0.726	0.005
4-Hydroxyglutamate transaminase inhibitor	0.724	0.004
Inulinase inhibitor	0.723	0.004
CYP2A8 substrate	0.724	0.006
Chlordecone reductase inhibitor	0.747	0.030
Peptide alpha-N-acetyltransferase inhibitor	0.723	0.006
Mucositis treatment	0.736	0.020
CYP2C12 substrate	0.761	0.045
Corticosteroid side-chain-isomerase inhibitor	0.723	0.007
Preneoplastic conditions treatment	0.721	0.007

Arylsulfate sulfotransferase inhibitor	0.727	0.012
NADH kinase inhibitor	0.719	0.006
Cutinase inhibitor	0.722	0.009
Horrilysin inhibitor	0.715	0.004
5 Hydroxytryptamine release stimulant	0.732	0.022
Serine-pyruvate transaminase inhibitor	0.712	0.003
2-Haloacid dehalogenase inhibitor	0.712	0.004
Mitochondrial processing peptidase inhibitor	0.716	0.009
(R)-6-hydroxynicotine oxidase inhibitor	0.714	0.007
Methanol dehydrogenase inhibitor	0.709	0.004
Tryptophanamidase inhibitor	0.710	0.005
Glyoxylate reductase inhibitor	0.710	0.005
Cyclohexyl-isocyanide hydratase inhibitor	0.709	0.005
Aminocarboxymuconate-semialdehyde decarboxylase inhibitor	0.708	0.004
Glucan 1,4-alpha-maltotetraohydrolase inhibitor	0.706	0.005
Histidinol-phosphatase inhibitor	0.705	0.005
NAD(P)+-arginine ADP-ribosyltransferase inhibitor	0.710	0.011
Biotinidase inhibitor	0.705	0.007
Alopecia treatment	0.704	0.006
Peroxidase inhibitor	0.709	0.011
Mannan endo-1,4-beta-mannosidase inhibitor	0.701	0.005
Thiol oxidase inhibitor	0.701	0.005
Lactase inhibitor	0.701	0.006
27-Hydroxycholesterol 7alpha-monooxygenase inhibitor	0.707	0.012
Linoleate diol synthase inhibitor	0.704	0.011
Lysase inhibitor	0.714	0.021
Peptidyl-dipeptidase Dcp inhibitor	0.709	0.016
Membrane permeability inhibitor	0.719	0.031
JAK2 expression inhibitor	0.701	0.017

Table S4. Prediction of the selected activity (Pa > 0.7) of molecule (**2c**) using PASS software. The results are expressed as probability to be active (Pa) or inactive (Pi).

	**Pa**	**Pi**
Integrin alphaVbeta3 antagonist	0.909	0.000
Aspulvinone dimethylallyltransferase inhibitor	0.913	0.005
NADPH peroxidase inhibitor	0.903	0.004
Phobic disorders treatment	0.904	0.005
Chloride peroxidase inhibitor	0.882	0.002
Fatty-acyl-CoA synthase inhibitor	0.871	0.003
Complement factor D inhibitor	0.870	0.004
Cl--transporting ATPase inhibitor	0.867	0.004
Antibiotic Glycopeptide-like	0.860	0.002
Acylcarnitine hydrolase inhibitor	0.859	0.007
Arylacetonitrilase inhibitor	0.848	0.006
Fusarinine-C ornithinesterase inhibitor	0.840	0.004
Saccharopepsin inhibitor	0.847	0.012
Acrocylindropepsin inhibitor	0.847	0.012
Chymosin inhibitor	0.847	0.012
Glucose oxidase inhibitor	0.842	0.008
Polyamine-transporting ATPase inhibitor	0.837	0.004
S-alkylcysteine lyase inhibitor	0.833	0.003
Ubiquinol-cytochrome-c reductase inhibitor	0.846	0.018
Testosterone 17beta-dehydrogenase (NADP+) inhibitor	0.842	0.016
Venombin AB inhibitor	0.824	0.005
Pterin deaminase inhibitor	0.822	0.004
Gluconate 2-dehydrogenase (acceptor) inhibitor	0.827	0.011
Beta-adrenergic receptor kinase inhibitor	0.823	0.011
G-protein-coupled receptor kinase inhibitor	0.823	0.011
Chlordecone reductase inhibitor	0.827	0.015
5 Hydroxytryptamine release stimulant	0.822	0.012
Glycosylphosphatidylinositol phospholipase D inhibitor	0.819	0.010
Glutamine-phenylpyruvate transaminase inhibitor	0.814	0.004
Sugar-phosphatase inhibitor	0.822	0.013
Omptin inhibitor	0.812	0.006
UDP-N-acetylglucosamine 4-epimerase inhibitor	0.810	0.005
Dimethylargininase inhibitor	0.810	0.005
Glutamyl endopeptidase II inhibitor	0.813	0.008
NADPH-cytochrome-c2 reductase inhibitor	0.809	0.005
Exoribonuclease II inhibitor	0.809	0.007
Fragilysin inhibitor	0.809	0.007
Arginine 2-monooxygenase inhibitor	0.808	0.008
Limulus clotting factor B inhibitor	0.803	0.004
Mucomembranous protector	0.813	0.015
5-O-(4-coumaroyl)-D-quinate 3'-monooxygenase inhibitor	0.801	0.007
NADH kinase inhibitor	0.796	0.004
Nicotinic alpha6beta3beta4alpha5 receptor antagonist	0.802	0.011
Pseudolysin inhibitor	0.796	0.007

Kynurenine 3 monooxygenase inhibitor	0.790	0.000
Pro-opiomelanocortin converting enzyme inhibitor	0.803	0.014
Chemoprotective	0.790	0.002
Methylenetetrahydrofolate reductase (NADPH) inhibitor	0.806	0.018
Antiseborrheic	0.804	0.018
Carboxypeptidase Taq inhibitor	0.793	0.008
Phthalate 4.5-dioxygenase inhibitor	0.790	0.005
GST A substrate	0.794	0.010
Dehydro-L-gulonate decarboxylase inhibitor	0.791	0.010
Nicotinic alpha2beta2 receptor antagonist	0.792	0.011
Fibrolase inhibitor	0.784	0.004
Sphinganine kinase inhibitor	0.794	0.014
Hepatic disorders treatment	0.780	0.004
Sulfite reductase inhibitor	0.779	0.004
Hydrogen dehydrogenase inhibitor	0.779	0.005
Albendazole monooxygenase inhibitor	0.777	0.003
L-glutamate oxidase inhibitor	0.777	0.005
Aldehyde dehydrogenase (pyrroloquinoline-quinone) inhibitor	0.777	0.004
N-benzyloxycarbonylglycine hydrolase inhibitor	0.773	0.006
Manganese peroxidase inhibitor	0.772	0.006
Phospholipid-translocating ATPase inhibitor	0.772	0.006
Ribulose-phosphate 3-epimerase inhibitor	0.773	0.010
Aspartate-ammonia ligase inhibitor	0.767	0.005
Lysine 2.3-aminomutase inhibitor	0.770	0.008
IgA-specific serine endopeptidase inhibitor	0.770	0.008
Thioredoxin inhibitor	0.766	0.005
Mucinaminylserine mucinaminidase inhibitor	0.768	0.007
Threonine aldolase inhibitor	0.766	0.005
Styrene-oxide isomerase inhibitor	0.763	0.003
Membrane integrity agonist	0.797	0.037
Fructan beta-fructosidase inhibitor	0.763	0.004
Limulus clotting factor C inhibitor	0.765	0.007
Macrophage colony stimulating factor agonist	0.763	0.007
Creatininase inhibitor	0.761	0.008
Bisphosphoglycerate phosphatase inhibitor	0.761	0.009
Sulfite oxidase inhibitor	0.755	0.004
Amine dehydrogenase inhibitor	0.756	0.005
CYP2J substrate	0.775	0.027
Formaldehyde transketolase inhibitor	0.751	0.007
Taurine dehydrogenase inhibitor	0.763	0.019
Superoxide dismutase inhibitor	0.752	0.009
D-alanine 2-hydroxymethyltransferase inhibitor	0.746	0.003
Histidine N-acetyltransferase inhibitor	0.744	0.006
Membrane permeability inhibitor	0.756	0.019
Glucan 1.4-alpha-maltotriohydrolase inhibitor	0.742	0.006
Lysostaphin inhibitor	0.741	0.006
Taurine-2-oxoglutarate transaminase inhibitor	0.737	0.003
Peptide-N4-(N-acetyl-beta-glucosaminyl)asparagine amidase inhibitor	0.740	0.006

Aspartate-phenylpyruvate transaminase inhibitor	0.739	0.006
Phosphatidylserine decarboxylase inhibitor	0.739	0.008
Endopeptidase So inhibitor	0.731	0.005
Meprin B inhibitor	0.729	0.003
Polyporopepsin inhibitor	0.754	0.028
Pullulanase inhibitor	0.738	0.013
Glucan endo-1.6-beta-glucosidase inhibitor	0.738	0.014
Alkylacetylglycerophosphatase inhibitor	0.738	0.015
CDP-glycerol glycerophosphotransferase inhibitor	0.761	0.039
Glutathione thiolesterase inhibitor	0.735	0.014
ADP-thymidine kinase inhibitor	0.732	0.012
Electron-transferring-flavoprotein dehydrogenase inhibitor	0.727	0.009
Glycine dehydrogenase (decarboxylating) inhibitor	0.720	0.004
Carminative	0.720	0.006
Nucleoside oxidase (H2O2-forming) inhibitor	0.724	0.010
CYP2J2 substrate	0.738	0.024
Methylumbelliferyl-acetate deacetylase inhibitor	0.721	0.007
4-Nitrophenol 2-monooxygenase inhibitor	0.717	0.007
D-xylulose reductase inhibitor	0.713	0.004
3-Hydroxybenzoate 6-monooxygenase inhibitor	0.717	0.010
6-Pyruvoyltetrahydropterin synthase inhibitor	0.711	0.004
Methylamine-glutamate N-methyltransferase inhibitor	0.715	0.010
tRNA-pseudouridine synthase I inhibitor	0.712	0.007
2-Dehydropantoate 2-reductase inhibitor	0.718	0.014
Phosphatidylcholine-retinol O-acyltransferase inhibitor	0.717	0.013
Glyoxylate reductase inhibitor	0.709	0.005
Gluconate 5-dehydrogenase inhibitor	0.713	0.010
GABA C receptor agonist	0.706	0.003
Acetylesterase inhibitor	0.712	0.011
Leukopoiesis stimulant	0.706	0.006
Feruloyl esterase inhibitor	0.718	0.020
(S)-6-hydroxynicotine oxidase inhibitor	0.704	0.006
Ecdysone 20-monooxygenase inhibitor	0.705	0.008
Cutinase inhibitor	0.702	0.010
Arylsulfate sulfotransferase inhibitor	0.703	0.014
Fucosterol-epoxide lyase inhibitor	0.701	0.015
Alkenylglycerophosphocholine hydrolase inhibitor	0.708	0.030
CYP2C12 substrate	0.717	0.054

Table S5. Prediction of the selected activity (Pa > 0.7) of molecule (**2d**) using PASS software. The results are expressed as probability to be active (Pa) or inactive (Pi).

	**Pa**	**Pi**
Fatty-acyl-CoA synthase inhibitor	0.886	0.003
Mycothiol-S-conjugate amidase inhibitor	0.883	0.002
Beta-adrenergic receptor kinase inhibitor	0.869	0.007
G-protein-coupled receptor kinase inhibitor	0.869	0.007
Aspulvinone dimethylallyltransferase inhibitor	0.875	0.014
Complement factor D inhibitor	0.840	0.004
Phobic disorders treatment	0.849	0.017
NADPH peroxidase inhibitor	0.834	0.008
Chloride peroxidase inhibitor	0.822	0.004
Mucomembranous protector	0.823	0.013
Cl--transporting ATPase inhibitor	0.790	0.007
Venombin AB inhibitor	0.789	0.007
Omptin inhibitor	0.783	0.009
Polyamine-transporting ATPase inhibitor	0.775	0.006
Limulus clotting factor B inhibitor	0.773	0.006
Acylcarnitine hydrolase inhibitor	0.775	0.016
Fusarinine-C ornithinesterase inhibitor	0.769	0.011
Carminative	0.758	0.005
Testosterone 17beta-dehydrogenase (NADP+) inhibitor	0.781	0.030
Ubiquinol-cytochrome-c reductase inhibitor	0.786	0.038
Pterin deaminase inhibitor	0.750	0.007
Glucose oxidase inhibitor	0.753	0.018
Glutamine-phenylpyruvate transaminase inhibitor	0.741	0.008
NADPH-cytochrome-c2 reductase inhibitor	0.742	0.012
Macrophage colony stimulating factor agonist	0.737	0.009
UDP-N-acetylglucosamine 4-epimerase inhibitor	0.738	0.012
Dimethylargininase inhibitor	0.736	0.011
Glutamyl endopeptidase II inhibitor	0.743	0.019
GST A substrate	0.741	0.017
Sugar-phosphatase inhibitor	0.748	0.025
S-alkylcysteine lyase inhibitor	0.729	0.006
Chymosin inhibitor	0.751	0.031
Saccharopepsin inhibitor	0.751	0.031
Acrocylindropepsin inhibitor	0.751	0.031
Limulus clotting factor C inhibitor	0.720	0.009
NADH kinase inhibitor	0.716	0.006
Phosphoenolpyruvate-protein phosphotransferase inhibitor	0.711	0.003
Chlordecone reductase inhibitor	0.740	0.032
GABA aminotransferase inhibitor	0.711	0.004
Exoribonuclease II inhibitor	0.719	0.015
Nicotinic alpha6beta3beta4alpha5 receptor antagonist	0.726	0.027
CYP2J substrate	0.736	0.038
Arginine 2-monooxygenase inhibitor	0.715	0.018
Hydrogen dehydrogenase inhibitor	0.706	0.009

Albendazole monooxygenase inhibitor	0.701	0.004
Pro-opiomelanocortin converting enzyme inhibitor	0.715	0.026
Membrane permeability inhibitor	0.719	0.031
Antieczematic	0.725	0.038
Lysine 2.3-aminomutase inhibitor	0.702	0.015
5-O-(4-coumaroyl)-D-quinate 3'-monooxygenase inhibitor	0.706	0.022
Glycosylphosphatidylinositol phospholipase D inhibitor	0.710	0.033
CDP-glycerol glycerophosphotransferase inhibitor	0.715	0.050

Table S6. Prediction of the selected activity (Pa > 0.7) of molecule (**3a**) using PASS software. The results are expressed as probability to be active (Pa) or inactive (Pi).

	Pa	Pi
Fatty-acyl-CoA synthase inhibitor	0.935	0.001
Aspulvinone dimethylallyltransferase inhibitor	0.916	0.005
Carminative	0.888	0.002
Ubiquinol-cytochrome-c reductase inhibitor	0.889	0.007
Beta-adrenergic receptor kinase inhibitor	0.887	0.005
G-protein-coupled receptor kinase inhibitor	0.887	0.005
Cl--transporting ATPase inhibitor	0.859	0.004
Phosphatidylcholine-retinol O-acyltransferase inhibitor	0.849	0.004
CYP2E1 substrate	0.844	0.004
Antineoplastic	0.844	0.007
CYP2E substrate	0.839	0.004
All-trans-retinyl-palmitate hydrolase inhibitor	0.835	0.004
CYP2J substrate	0.842	0.012
Apoptosis agonist	0.833	0.006
Phosphoenolpyruvate-protein phosphotransferase inhibitor	0.827	0.002
Antieczematic	0.834	0.012
Testosterone 17beta-dehydrogenase (NADP+) inhibitor	0.837	0.017
Sugar-phosphatase inhibitor	0.828	0.012
NADH kinase inhibitor	0.818	0.003
Glutamyl endopeptidase II inhibitor	0.817	0.008
Chloride peroxidase inhibitor	0.813	0.004
Complement factor D inhibitor	0.814	0.005
Gluconate 2-dehydrogenase (acceptor) inhibitor	0.816	0.013
Chlordecone reductase inhibitor	0.817	0.017
5-O-(4-coumaroyl)-D-quinate 3'-monooxygenase inhibitor	0.802	0.007
Acrocylindropepsin inhibitor	0.812	0.018
Saccharopepsin inhibitor	0.812	0.018
Chymosin inhibitor	0.812	0.018
Mucomembranous protector	0.810	0.016
Feruloyl esterase inhibitor	0.804	0.011
Macrophage colony stimulating factor agonist	0.798	0.005
Respiratory analeptic	0.801	0.008
Ribulose-phosphate 3-epimerase inhibitor	0.797	0.007
Phobic disorders treatment	0.810	0.030
Dehydro-L-gulonate decarboxylase inhibitor	0.788	0.010
UDP-N-acetylglucosamine 4-epimerase inhibitor	0.775	0.008
Carboxypeptidase Taq inhibitor	0.773	0.010
TP53 expression enhancer	0.774	0.014
Antineoplastic (breast cancer)	0.759	0.005
Fusarinine-C ornithinesterase inhibitor	0.766	0.011
Alkylacetylglycerophosphatase inhibitor	0.766	0.013
Electron-transferring-flavoprotein dehydrogenase inhibitor	0.755	0.006
CYP2J2 substrate	0.765	0.019
Gluconate 5-dehydrogenase inhibitor	0.753	0.007

Bisphosphoglycerate phosphatase inhibitor	0.753	0.009
Polyamine-transporting ATPase inhibitor	0.748	0.008
GST A substrate	0.755	0.015
2-Hydroxymuconate-semialdehyde hydrolase inhibitor	0.748	0.010
Acylcarnitine hydrolase inhibitor	0.757	0.018
IgA-specific serine endopeptidase inhibitor	0.746	0.010
Aminocarboxymuconate-semialdehyde decarboxylase inhibitor	0.740	0.004
6-Pyruvoyltetrahydropterin synthase inhibitor	0.739	0.004
General pump inhibitor	0.739	0.005
Chenodeoxycholoyltaurine hydrolase inhibitor	0.739	0.005
Phospholipid-translocating ATPase inhibitor	0.742	0.009
Antineoplastic (lung cancer)	0.734	0.005
NADPH-cytochrome-c2 reductase inhibitor	0.739	0.012
NADPH peroxidase inhibitor	0.746	0.020
Glutathione thiolesterase inhibitor	0.738	0.013
Acetylesterase inhibitor	0.731	0.009
Lysostaphin inhibitor	0.728	0.007
Membrane permeability inhibitor	0.744	0.023
Glucan 1.4-alpha-maltotriohydrolase inhibitor	0.727	0.007
Crotonoyl-[acyl-carrier-protein] hydratase inhibitor	0.723	0.005
Cutinase inhibitor	0.727	0.009
Glycosylphosphatidylinositol phospholipase D inhibitor	0.742	0.025
Dimethylargininase inhibitor	0.725	0.012
Alkenylglycerophosphocholine hydrolase inhibitor	0.739	0.026
2-Haloacid dehalogenase inhibitor	0.717	0.004
Allyl-alcohol dehydrogenase inhibitor	0.717	0.005
Steroid N-acetylglucosaminyltransferase inhibitor	0.716	0.005
4-Nitrophenol 2-monooxygenase inhibitor	0.716	0.007
Methylamine-glutamate N-methyltransferase inhibitor	0.715	0.010
Glucan endo-1.6-beta-glucosidase inhibitor	0.720	0.017
Polyneuridine-aldehyde esterase inhibitor	0.706	0.004
Omptin inhibitor	0.719	0.018
Limulus clotting factor B inhibitor	0.712	0.012
tRNA-pseudouridine synthase I inhibitor	0.707	0.007
Venombin AB inhibitor	0.713	0.015
Arginine 2-monooxygenase inhibitor	0.714	0.018
Thioredoxin inhibitor	0.706	0.010
Lipid metabolism regulator	0.706	0.010
Analeptic	0.705	0.010
Nicotinic alpha6beta3beta4alpha5 receptor antagonist	0.720	0.029
CYP2A6 substrate	0.701	0.009
Pro-opiomelanocortin converting enzyme inhibitor	0.713	0.026
Fragilysin inhibitor	0.702	0.017
Alkane 1-monooxygenase inhibitor	0.701	0.016
Polyporopepsin inhibitor	0.715	0.036
Taurine dehydrogenase inhibitor	0.706	0.030

Table S7. Prediction of the selected activity (Pa > 0.7) of molecule (**3b**) using PASS software. The results are expressed as probability to be active (Pa) or inactive (Pi).

	Pa	Pi
Ubiquinol-cytochrome-c reductase inhibitor	0.934	0.003
Fatty-acyl-CoA synthase inhibitor	0.919	0.002
Aspulvinone dimethylallyltransferase inhibitor	0.917	0.005
Phosphatidylcholine-retinol O-acyltransferase inhibitor	0.910	0.002
All-trans-retinyl-palmitate hydrolase inhibitor	0.901	0.002
CYP2J substrate	0.899	0.005
Testosterone 17beta-dehydrogenase (NADP+) inhibitor	0.897	0.007
Sugar-phosphatase inhibitor	0.892	0.005
Feruloyl esterase inhibitor	0.890	0.004
Complement factor D inhibitor	0.886	0.003
Glutamyl endopeptidase II inhibitor	0.885	0.004
Xenobiotic-transporting ATPase inhibitor	0.880	0.002
Phobic disorders treatment	0.885	0.008
Chymosin inhibitor	0.883	0.007
Acrocylindropepsin inhibitor	0.883	0.007
Saccharopepsin inhibitor	0.883	0.007
5-O-(4-coumaroyl)-D-quinate 3'-monooxygenase inhibitor	0.874	0.003
Ribulose-phosphate 3-epimerase inhibitor	0.873	0.004
Dehydro-L-gulonate decarboxylase inhibitor	0.870	0.004
2-Hydroxymuconate-semialdehyde hydrolase inhibitor	0.868	0.004
Macrophage colony stimulating factor agonist	0.865	0.003
Cl--transporting ATPase inhibitor	0.864	0.004
UDP-N-acetylglucosamine 4-epimerase inhibitor	0.861	0.004
Carboxypeptidase Taq inhibitor	0.860	0.004
Carminative	0.859	0.003
Alkylacetylglycerophosphatase inhibitor	0.857	0.005
Bisphosphoglycerate phosphatase inhibitor	0.855	0.004
IgA-specific serine endopeptidase inhibitor	0.853	0.004
Membrane integrity agonist	0.867	0.020
CYP2J2 substrate	0.851	0.007
Fusarinine-C ornithinesterase inhibitor	0.848	0.004
Alkenylglycerophosphocholine hydrolase inhibitor	0.854	0.010
Cutinase inhibitor	0.847	0.004
Acetylesterase inhibitor	0.847	0.004
Allyl-alcohol dehydrogenase inhibitor	0.845	0.003
NADPH peroxidase inhibitor	0.848	0.006
Aminocarboxymuconate-semialdehyde decarboxylase inhibitor	0.844	0.002
Acylcarnitine hydrolase inhibitor	0.848	0.008
Gluconate 5-dehydrogenase inhibitor	0.842	0.003
Polyamine-transporting ATPase inhibitor	0.842	0.004
Creatininase inhibitor	0.842	0.004
Chenodeoxycholoyltaurine hydrolase inhibitor	0.840	0.003
Electron-transferring-flavoprotein dehydrogenase inhibitor	0.839	0.003
Glutathione thiolesterase inhibitor	0.840	0.004

Pullulanase inhibitor	0.840	0.005
Glucan endo-1.6-beta-glucosidase inhibitor	0.840	0.005
Taurine dehydrogenase inhibitor	0.841	0.007
Phospholipid-translocating ATPase inhibitor	0.836	0.004
Glycosylphosphatidylinositol phospholipase D inhibitor	0.839	0.007
NADPH-cytochrome-c2 reductase inhibitor	0.835	0.004
CYP2C12 substrate	0.854	0.023
Crotonoyl-[acyl-carrier-protein] hydratase inhibitor	0.833	0.003
Lysostaphin inhibitor	0.833	0.003
Arginine 2-monooxygenase inhibitor	0.834	0.006
Fragilysin inhibitor	0.831	0.005
Dimethylargininase inhibitor	0.830	0.005
3-Hydroxybenzoate 6-monooxygenase inhibitor	0.828	0.004
Venombin AB inhibitor	0.826	0.005
Arylacetonitrilase inhibitor	0.829	0.008
Methylamine-glutamate N-methyltransferase inhibitor	0.824	0.004
Polyneuridine-aldehyde esterase inhibitor	0.821	0.003
NADH kinase inhibitor	0.821	0.003
Glucan 1.4-alpha-maltotriohydrolase inhibitor	0.820	0.004
tRNA-pseudouridine synthase I inhibitor	0.820	0.003
Nicotinic alpha6beta3beta4alpha5 receptor antagonist	0.824	0.008
Polyporopepsin inhibitor	0.830	0.015
Carboxylate reductase inhibitor	0.816	0.002
Pro-opiomelanocortin converting enzyme inhibitor	0.825	0.011
2-Haloacid dehalogenase inhibitor	0.816	0.002
Cyclohexyl-isocyanide hydratase inhibitor	0.815	0.003
Chloride peroxidase inhibitor	0.815	0.004
Alkane 1-monooxygenase inhibitor	0.816	0.005
Ferredoxin-NAD+ reductase inhibitor	0.810	0.003
Naphthalene 1.2-dioxygenase inhibitor	0.810	0.003
Prolyl aminopeptidase inhibitor	0.813	0.007
Chlordecone reductase inhibitor	0.822	0.016
Thioredoxin inhibitor	0.809	0.004
N-Acyl-D-aspartate deacylase inhibitor	0.807	0.003
Shikimate O-hydroxycinnamoyltransferase inhibitor	0.807	0.004
Limulus clotting factor B inhibitor	0.807	0.004
N-formylmethionyl-peptidase inhibitor	0.805	0.003
Omptin inhibitor	0.808	0.007
Gamma-guanidinobutyraldehyde dehydrogenase inhibitor	0.805	0.004
Sphinganine kinase inhibitor	0.813	0.012
N-acetylneuraminate 7-O(or 9-O)-acetyltransferase inhibitor	0.806	0.006
G-protein-coupled receptor kinase inhibitor	0.809	0.012
Beta-adrenergic receptor kinase inhibitor	0.809	0.012
Long-chain-aldehyde dehydrogenase inhibitor	0.798	0.003
Antiseborrheic	0.810	0.017
Arylmalonate decarboxylase inhibitor	0.795	0.003
Phenol O-methyltransferase inhibitor	0.794	0.004
Poly(alpha-L-guluronate) lyase inhibitor	0.793	0.005

Opheline kinase inhibitor	0.791	0.003
Taurocyamine kinase inhibitor	0.791	0.003
Exoribonuclease II inhibitor	0.794	0.008
Nicotinic alpha2beta2 receptor antagonist	0.796	0.010
Glucose oxidase inhibitor	0.798	0.012
4-Hydroxyglutamate transaminase inhibitor	0.788	0.003
Phthalate 4.5-dioxygenase inhibitor	0.789	0.005
Pterin deaminase inhibitor	0.788	0.005
Methylenetetrahydrofolate reductase (NADPH) inhibitor	0.801	0.018
BRAF expression inhibitor	0.783	0.002
General pump inhibitor	0.784	0.004
27-Hydroxycholesterol 7alpha-monooxygenase inhibitor	0.786	0.006
2-Hydroxyquinoline 8-monooxygenase inhibitor	0.785	0.006
Phosphoenolpyruvate-protein phosphotransferase inhibitor	0.780	0.003
2-Hydroxy-3-oxoadipate synthase inhibitor	0.780	0.003
Aspartate-ammonia ligase inhibitor	0.781	0.004
L-glutamate oxidase inhibitor	0.781	0.005
Tryptophanamidase inhibitor	0.778	0.004
Aldehyde ferredoxin oxidoreductase inhibitor	0.775	0.002
ADP-thymidine kinase inhibitor	0.782	0.009
Methanol dehydrogenase inhibitor	0.776	0.003
Sulfite reductase inhibitor	0.777	0.004
N-acylmannosamine kinase inhibitor	0.776	0.004
2-Haloacid dehalogenase (configuration-inverting) inhibitor	0.774	0.004
Pseudolysin inhibitor	0.779	0.008
IgA-specific metalloendopeptidase inhibitor	0.774	0.005
Lysine 2.3-aminomutase inhibitor	0.777	0.008
Spermidine dehydrogenase inhibitor	0.772	0.005
Xylan endo-1.3-beta-xylosidase inhibitor	0.771	0.005
Arylalkyl acylamidase inhibitor	0.769	0.004
Aminobutyraldehyde dehydrogenase inhibitor	0.768	0.004
Carbon-monoxide dehydrogenase inhibitor	0.766	0.003
Snapalysin inhibitor	0.766	0.003
CYP2B5 substrate	0.767	0.005
GST A substrate	0.771	0.013
Trimethylamine-oxide aldolase inhibitor	0.762	0.004
Formaldehyde transketolase inhibitor	0.763	0.006
Peptide alpha-N-acetyltransferase inhibitor	0.762	0.005
Poly(beta-D-mannuronate) lyase inhibitor	0.759	0.004
Limulus clotting factor C inhibitor	0.761	0.007
Mucomembranous protector	0.779	0.025
Sorbitol-6-phosphate 2-dehydrogenase inhibitor	0.755	0.003
Benzaldehyde dehydrogenase (NADP+) inhibitor	0.754	0.003
Glutarate-semialdehyde dehydrogenase inhibitor	0.755	0.004
Fucosterol-epoxide lyase inhibitor	0.761	0.010
1.4-Lactonase inhibitor	0.755	0.005
Aspartate-phenylpyruvate transaminase inhibitor	0.755	0.005
Peptide-N4-(N-acetyl-beta-glucosaminyl)asparagine amidase inhibitor	0.754	0.005

N-hydroxy-2-acetamidofluorene reductase inhibitor	0.750	0.003
Arylsulfate sulfotransferase inhibitor	0.756	0.010
Aryldialkylphosphatase inhibitor	0.748	0.004
CYP2A8 substrate	0.749	0.005
Glutamine-phenylpyruvate transaminase inhibitor	0.751	0.007
Tpr proteinase (Porphyromonas gingivalis) inhibitor	0.748	0.004
CYP2D16 substrate	0.747	0.004
JAK2 expression inhibitor	0.754	0.012
Mannitol-1-phosphatase inhibitor	0.745	0.003
2-Oxoaldehyde dehydrogenase (NADP+) inhibitor	0.744	0.003
TP53 expression enhancer	0.757	0.016
Phosphatidate phosphatase inhibitor	0.743	0.005
Gluconolactonase inhibitor	0.742	0.003
6-Pyruvoyltetrahydropterin synthase inhibitor	0.742	0.004
Dolichyl-phosphatase inhibitor	0.741	0.003
Antineoplastic	0.756	0.018
Glycolate dehydrogenase inhibitor	0.739	0.003
Mannan endo-1.6-alpha-mannosidase inhibitor	0.739	0.003
Nitrite reductase (NO-forming) inhibitor	0.739	0.004
Apoptosis agonist	0.746	0.011
4-Chlorophenylacetate 3.4-dioxygenase inhibitor	0.738	0.004
Glucan 1.4-alpha-maltotetraohydrolase inhibitor	0.737	0.004
Sulfite oxidase inhibitor	0.737	0.004
Nitrate reductase (cytochrome) inhibitor	0.741	0.009
S-alkylcysteine lyase inhibitor	0.736	0.006
Protein-disulfide reductase (glutathione) inhibitor	0.745	0.015
Ornithine cyclodeaminase inhibitor	0.733	0.003
Clavaminate synthase inhibitor	0.732	0.003
(S)-6-hydroxynicotine oxidase inhibitor	0.733	0.005
Phosphatidylserine decarboxylase inhibitor	0.736	0.008
4-Nitrophenol 2-monooxygenase inhibitor	0.732	0.006
N-benzyloxycarbonylglycine hydrolase inhibitor	0.733	0.008
Mannan endo-1.4-beta-mannosidase inhibitor	0.729	0.004
2-Dehydropantoate 2-reductase inhibitor	0.736	0.012
Respiratory analeptic	0.736	0.012
Endopeptidase So inhibitor	0.726	0.005
Dextranase inhibitor	0.728	0.008
Centromere associated protein inhibitor	0.725	0.006
Alkylglycerone-phosphate synthase inhibitor	0.723	0.005
Prostaglandin-A1 DELTA-isomerase inhibitor	0.723	0.005
Myeloblastin inhibitor	0.721	0.003
Histidine N-acetyltransferase inhibitor	0.722	0.007
Superoxide dismutase inhibitor	0.725	0.011
Fibrolase inhibitor	0.720	0.006
Di-trans.poly-cis-decaprenylcistransferase inhibitor	0.718	0.004
Mucinaminylserine mucinaminidase inhibitor	0.722	0.009
Methylumbelliferyl-acetate deacetylase inhibitor	0.720	0.007
Camphor 1.2-monooxygenase inhibitor	0.715	0.004

Opine dehydrogenase inhibitor	0.713	0.004
Anthranilate-CoA ligase inhibitor	0.713	0.005
Plastoquinol-plastocyanin reductase inhibitor	0.711	0.003
Amine dehydrogenase inhibitor	0.715	0.007
Mitochondrial processing peptidase inhibitor	0.715	0.009
Lactaldehyde reductase inhibitor	0.709	0.004
Glycine dehydrogenase (decarboxylating) inhibitor	0.706	0.004
3-Carboxyethylcatechol 2.3-dioxygenase inhibitor	0.707	0.004
Corticosteroid side-chain-isomerase inhibitor	0.710	0.008
Glyoxylate reductase inhibitor	0.707	0.005
Aldehyde dehydrogenase (pyrroloquinoline-quinone) inhibitor	0.707	0.006
Dimethylmaleate hydratase inhibitor	0.704	0.004
Trans-pentaprenyltranstransferase inhibitor	0.702	0.003
Urethanase inhibitor	0.705	0.008
Leukotriene-B4 20-monooxygenase inhibitor	0.701	0.004
Membrane permeability inhibitor	0.725	0.029
Antiinflammatory	0.710	0.014
Hydrogen dehydrogenase inhibitor	0.705	0.009
Kidney function stimulant	0.702	0.009
Glucan endo-1.3-beta-D-glucosidase inhibitor	0.703	0.011
Linoleate diol synthase inhibitor	0.702	0.011
Lysase inhibitor	0.709	0.022

Table S8. Prediction of the selected activity (Pa > 0.7) of molecule (**3c**) using PASS software. The results are expressed as probability to be active (Pa) or inactive (Pi).

`	Pa	Pi
Aspulvinone dimethylallyltransferase inhibitor	0.965	0.002
Cl--transporting ATPase inhibitor	0.918	0.002
Fatty-acyl-CoA synthase inhibitor	0.903	0.002
Cardiotonic	0.902	0.004
Chlordecone reductase inhibitor	0.900	0.005
Gluconate 2-dehydrogenase (acceptor) inhibitor	0.884	0.005
Testosterone 17beta-dehydrogenase (NADP+) inhibitor	0.885	0.008
Sugar-phosphatase inhibitor	0.879	0.006
Ubiquinol-cytochrome-c reductase inhibitor	0.879	0.009
Complement factor D inhibitor	0.871	0.004
Glutamyl endopeptidase II inhibitor	0.871	0.004
Phosphatidylcholine-retinol O-acyltransferase inhibitor	0.870	0.004
NADH kinase inhibitor	0.868	0.002
Phobic disorders treatment	0.870	0.011
5-O-(4-coumaroyl)-D-quinate 3'-monooxygenase inhibitor	0.854	0.004
Dehydro-L-gulonate decarboxylase inhibitor	0.854	0.005
CYP2J substrate	0.858	0.009
All-trans-retinyl-palmitate hydrolase inhibitor	0.851	0.003
Ribulose-phosphate 3-epimerase inhibitor	0.851	0.004
Acrocylindropepsin inhibitor	0.855	0.011
Chymosin inhibitor	0.855	0.011
Saccharopepsin inhibitor	0.855	0.011
Chloride peroxidase inhibitor	0.845	0.003
2-Hydroxymuconate-semialdehyde hydrolase inhibitor	0.845	0.004
Carboxypeptidase Taq inhibitor	0.843	0.004
Carminative	0.841	0.003
Mucomembranous protector	0.844	0.010
Feruloyl esterase inhibitor	0.841	0.008
UDP-N-acetylglucosamine 4-epimerase inhibitor	0.836	0.004
IgA-specific serine endopeptidase inhibitor	0.830	0.005
Bisphosphoglycerate phosphatase inhibitor	0.829	0.005
Membrane integrity agonist	0.849	0.024
G-protein-coupled receptor kinase inhibitor	0.832	0.010
Beta-adrenergic receptor kinase inhibitor	0.832	0.010
Alkenylglycerophosphocholine hydrolase inhibitor	0.833	0.013
Allyl-alcohol dehydrogenase inhibitor	0.823	0.004
Acylcarnitine hydrolase inhibitor	0.828	0.010
GST A substrate	0.824	0.007
Fusarinine-C ornithinesterase inhibitor	0.821	0.005
6-Pyruvoyltetrahydropterin synthase inhibitor	0.816	0.003
Alkylacetylglycerophosphatase inhibitor	0.820	0.008
Gluconate 5-dehydrogenase inhibitor	0.815	0.004
Glucan endo-1.6-beta-glucosidase inhibitor	0.817	0.006
Polyamine-transporting ATPase inhibitor	0.814	0.004

Creatininase inhibitor	0.815	0.005
Glutathione thiolesterase inhibitor	0.815	0.006
Lysostaphin inhibitor	0.812	0.004
Electron-transferring-flavoprotein dehydrogenase inhibitor	0.811	0.004
Acetylesterase inhibitor	0.811	0.005
Pullulanase inhibitor	0.813	0.007
Aldehyde dehydrogenase (pyrroloquinoline-quinone) inhibitor	0.809	0.004
NADPH-cytochrome-c2 reductase inhibitor	0.809	0.005
Arginine 2-monooxygenase inhibitor	0.811	0.008
Taurine dehydrogenase inhibitor	0.813	0.011
Chenodeoxycholoyltaurine hydrolase inhibitor	0.806	0.004
Glycosylphosphatidylinositol phospholipase D inhibitor	0.812	0.011
Fragilysin inhibitor	0.807	0.007
Amine dehydrogenase inhibitor	0.804	0.004
4-Nitrophenol 2-monooxygenase inhibitor	0.804	0.004
5 Hydroxytryptamine release stimulant	0.812	0.013
3-Hydroxybenzoate 6-monooxygenase inhibitor	0.804	0.005
Membrane permeability inhibitor	0.807	0.009
CYP2C12 substrate	0.828	0.029
Phospholipid-translocating ATPase inhibitor	0.803	0.005
Venombin AB inhibitor	0.803	0.005
Glucan 1.4-alpha-maltotriohydrolase inhibitor	0.800	0.004
Crotonoyl-[acyl-carrier-protein] hydratase inhibitor	0.797	0.003
NADPH peroxidase inhibitor	0.805	0.011
tRNA-pseudouridine synthase I inhibitor	0.797	0.004
Dimethylargininase inhibitor	0.799	0.006
Cutinase inhibitor	0.795	0.005
Methylamine-glutamate N-methyltransferase inhibitor	0.792	0.005
Polyneuridine-aldehyde esterase inhibitor	0.790	0.003
Nicotinic alpha6beta3beta4alpha5 receptor antagonist	0.798	0.011
Alkane 1-monooxygenase inhibitor	0.793	0.007
Cyclohexyl-isocyanide hydratase inhibitor	0.787	0.003
Thioredoxin inhibitor	0.788	0.004
Limulus clotting factor B inhibitor	0.787	0.005
Omptin inhibitor	0.789	0.009
Polyporopepsin inhibitor	0.799	0.020
Naphthalene 1.2-dioxygenase inhibitor	0.783	0.004
Ferredoxin-NAD+ reductase inhibitor	0.783	0.004
Pro-opiomelanocortin converting enzyme inhibitor	0.793	0.015
CYP2J2 substrate	0.790	0.014
Phosphoenolpyruvate-protein phosphotransferase inhibitor	0.778	0.003
N-formylmethionyl-peptidase inhibitor	0.776	0.004
N-acetylneuraminate 7-O(or 9-O)-acetyltransferase inhibitor	0.777	0.008
N-Acyl-D-aspartate deacylase inhibitor	0.773	0.004
Gamma-guanidinobutyraldehyde dehydrogenase inhibitor	0.771	0.005
Sphinganine kinase inhibitor	0.778	0.016
2-Haloacid dehalogenase inhibitor	0.764	0.003
Exoribonuclease II inhibitor	0.770	0.010

Arylmalonate decarboxylase inhibitor	0.764	0.004
Phenol O-methyltransferase inhibitor	0.764	0.005
Long-chain-aldehyde dehydrogenase inhibitor	0.764	0.004
Pterin deaminase inhibitor	0.763	0.006
Opheline kinase inhibitor	0.760	0.004
Taurocyamine kinase inhibitor	0.760	0.004
Phthalate 4.5-dioxygenase inhibitor	0.763	0.007
CYP2F1 substrate	0.759	0.004
Shikimate O-hydroxycinnamoyltransferase inhibitor	0.759	0.004
Arylacetonitrilase inhibitor	0.766	0.013
Antieczematic	0.774	0.024
Antiseborrheic	0.772	0.024
27-Hydroxycholesterol 7alpha-monooxygenase inhibitor	0.756	0.008
Poly(alpha-L-guluronate) lyase inhibitor	0.751	0.006
Pseudolysin inhibitor	0.755	0.011
L-glutamate oxidase inhibitor	0.749	0.007
Methylenetetrahydrofolate reductase (NADPH) inhibitor	0.764	0.023
Nicotinic alpha2beta2 receptor antagonist	0.756	0.016
Glucose oxidase inhibitor	0.756	0.017
Aspartate-ammonia ligase inhibitor	0.744	0.005
2-Hydroxyquinoline 8-monooxygenase inhibitor	0.748	0.009
Tryptophanamidase inhibitor	0.741	0.005
Carbon-monoxide dehydrogenase inhibitor	0.740	0.004
Methanol dehydrogenase inhibitor	0.739	0.004
Aminobutyraldehyde dehydrogenase inhibitor	0.739	0.004
GST P substrate	0.736	0.004
Lysine 2.3-aminomutase inhibitor	0.743	0.011
CYP2B5 substrate	0.738	0.007
Snapalysin inhibitor	0.735	0.004
Sulfite reductase inhibitor	0.737	0.006
Xylan endo-1.3-beta-xylosidase inhibitor	0.737	0.006
Trimethylamine-oxide aldolase inhibitor	0.736	0.005
Ecdysone 20-monooxygenase inhibitor	0.736	0.006
ADP-thymidine kinase inhibitor	0.741	0.011
N-acylmannosamine kinase inhibitor	0.733	0.005
Macrophage colony stimulating factor agonist	0.735	0.009
Limulus clotting factor C inhibitor	0.733	0.008
Poly(beta-D-mannuronate) lyase inhibitor	0.728	0.005
Glutarate-semialdehyde dehydrogenase inhibitor	0.727	0.004
1.4-Lactonase inhibitor	0.729	0.007
Formaldehyde transketolase inhibitor	0.731	0.008
IgA-specific metalloendopeptidase inhibitor	0.728	0.006
GST P1-1 substrate	0.723	0.003
CYP2D16 substrate	0.723	0.005
Spermidine dehydrogenase inhibitor	0.721	0.008
2-Haloacid dehalogenase (configuration-inverting) inhibitor	0.717	0.004
Glutamine-phenylpyruvate transaminase inhibitor	0.721	0.009
Mannitol-1-phosphatase inhibitor	0.716	0.004

Gluconolactonase inhibitor	0.715	0.004
Benzaldehyde dehydrogenase (NADP+) inhibitor	0.714	0.003
CYP2A8 substrate	0.716	0.006
Aspartate-phenylpyruvate transaminase inhibitor	0.716	0.008
2-Hydroxy-3-oxoadipate synthase inhibitor	0.712	0.004
Nitrite reductase (NO-forming) inhibitor	0.712	0.004
Prolyl aminopeptidase inhibitor	0.717	0.011
N-hydroxy-2-acetamidofluorene reductase inhibitor	0.710	0.003
Peptide alpha-N-acetyltransferase inhibitor	0.713	0.007
Arylalkyl acylamidase inhibitor	0.711	0.006
Glucan 1.4-alpha-maltotetraohydrolase inhibitor	0.707	0.004
Fucosterol-epoxide lyase inhibitor	0.716	0.014
Tpr proteinase (Porphyromonas gingivalis) inhibitor	0.706	0.005
Pediculicide	0.703	0.003
Protein-disulfide reductase (glutathione) inhibitor	0.718	0.018
Mannan endo-1.4-beta-mannosidase inhibitor	0.705	0.004
2-Oxoaldehyde dehydrogenase (NADP+) inhibitor	0.704	0.004
BRAF expression inhibitor	0.702	0.003
Glycolate dehydrogenase inhibitor	0.703	0.003
Sorbitol-6-phosphate 2-dehydrogenase inhibitor	0.702	0.003
Antiviral (Rhinovirus)	0.701	0.003
Aryldialkylphosphatase inhibitor	0.702	0.005
Peptide-N4-(N-acetyl-beta-glucosaminyl)asparagine amidase inhibitor	0.705	0.008
Arylsulfate sulfotransferase inhibitor	0.709	0.014
Phosphatidylserine decarboxylase inhibitor	0.707	0.011
Endopeptidase So inhibitor	0.701	0.007
Nitrate reductase (cytochrome) inhibitor	0.701	0.014

Table S9. Prediction of the selected activity (Pa > 0.7) of molecule (**3d**) using PASS software. The results are expressed as probability to be active (Pa) or inactive (Pi).

	Pa	Pi
Aspulvinone dimethylallyltransferase inhibitor	0.936	0.004
Fatty-acyl-CoA synthase inhibitor	0.914	0.002
G-protein-coupled receptor kinase inhibitor	0.911	0.004
Beta-adrenergic receptor kinase inhibitor	0.911	0.004
Apoptosis agonist	0.909	0.004
Mucomembranous protector	0.908	0.004
Antineoplastic	0.894	0.005
Phosphatidylcholine-retinol O-acyltransferase inhibitor	0.887	0.003
Ubiquinol-cytochrome-c reductase inhibitor	0.885	0.008
All-trans-retinyl-palmitate hydrolase inhibitor	0.873	0.003
CYP2J substrate	0.874	0.007
Carminative	0.861	0.003
CYP2E1 substrate	0.856	0.004
CYP2E substrate	0.851	0.004
Allyl-alcohol dehydrogenase inhibitor	0.848	0.003
Cl--transporting ATPase inhibitor	0.847	0.004
Membrane integrity agonist	0.859	0.022
Prenyl-diphosphatase inhibitor	0.832	0.003
Antineoplastic (breast cancer)	0.831	0.004
Testosterone 17beta-dehydrogenase (NADP+) inhibitor	0.839	0.017
Sugar-phosphatase inhibitor	0.827	0.012
Antieczematic	0.826	0.013
NADH kinase inhibitor	0.816	0.003
Glutamyl endopeptidase II inhibitor	0.820	0.008
Complement factor D inhibitor	0.816	0.005
Chlordecone reductase inhibitor	0.823	0.016
Retinol dehydrogenase inhibitor	0.808	0.001
Phosphoenolpyruvate-protein phosphotransferase inhibitor	0.803	0.002
Feruloyl esterase inhibitor	0.810	0.011
5-O-(4-coumaroyl)-D-quinate 3'-monooxygenase inhibitor	0.800	0.008
Gluconate 2-dehydrogenase (acceptor) inhibitor	0.807	0.015
CYP2A6 substrate	0.794	0.005
CDP-glycerol glycerophosphotransferase inhibitor	0.814	0.027
Ribulose-phosphate 3-epimerase inhibitor	0.794	0.008
Chloride peroxidase inhibitor	0.791	0.004
Dehydro-L-gulonate decarboxylase inhibitor	0.794	0.010
CYP2A substrate	0.789	0.006
BRAF expression inhibitor	0.784	0.002
Phobic disorders treatment	0.811	0.030
CYP2E1 inhibitor	0.771	0.004
Saccharopepsin inhibitor	0.789	0.023
Acrocylindropepsin inhibitor	0.789	0.023
Chymosin inhibitor	0.789	0.023
Antineoplastic (lung cancer)	0.771	0.005

Carboxypeptidase Taq inhibitor	0.775	0.009
UDP-N-acetylglucosamine 4-epimerase inhibitor	0.772	0.008
CYP2B6 substrate	0.771	0.009
Fusarinine-C ornithinesterase inhibitor	0.763	0.012
TP53 expression enhancer	0.764	0.015
CYP2J2 substrate	0.767	0.019
Bisphosphoglycerate phosphatase inhibitor	0.755	0.009
2-Hydroxymuconate-semialdehyde hydrolase inhibitor	0.755	0.009
Electron-transferring-flavoprotein dehydrogenase inhibitor	0.752	0.007
Gluconate 5-dehydrogenase inhibitor	0.750	0.007
6-Pyruvoyltetrahydropterin synthase inhibitor	0.744	0.004
Dolichyl-phosphatase inhibitor	0.741	0.003
Ecdysone 20-monooxygenase inhibitor	0.742	0.005
Transcription factor NF kappa B stimulant	0.740	0.004
Transcription factor stimulant	0.740	0.004
Polyamine-transporting ATPase inhibitor	0.745	0.008
2.3-Oxidosqualene-lanosterol cyclase inhibitor	0.737	0.001
GST A substrate	0.751	0.016
IgA-specific serine endopeptidase inhibitor	0.743	0.010
Glutathione thiolesterase inhibitor	0.745	0.013
Undecaprenyl-phosphate mannosyltransferase inhibitor	0.736	0.005
NADPH-cytochrome-c2 reductase inhibitor	0.742	0.012
Chenodeoxycholoyltaurine hydrolase inhibitor	0.735	0.005
Lysostaphin inhibitor	0.731	0.006
TRPA1 agonist	0.727	0.002
Phospholipid-translocating ATPase inhibitor	0.732	0.010
Alkenylglycerophosphocholine hydrolase inhibitor	0.747	0.025
Alkylacetylglycerophosphatase inhibitor	0.736	0.016
Membrane permeability inhibitor	0.743	0.023
Pediculicide	0.720	0.003
4-Nitrophenol 2-monooxygenase inhibitor	0.723	0.007
Steroid N-acetylglucosaminyltransferase inhibitor	0.721	0.005
Glycosylphosphatidylinositol phospholipase D inhibitor	0.741	0.025
Crotonoyl-[acyl-carrier-protein] hydratase inhibitor	0.718	0.005
tRNA-pseudouridine synthase I inhibitor	0.719	0.006
Protein-disulfide reductase (glutathione) inhibitor	0.728	0.017
Dimethylargininase inhibitor	0.722	0.012
Venombin AB inhibitor	0.720	0.014
Acylcarnitine hydrolase inhibitor	0.728	0.022
Limulus clotting factor B inhibitor	0.716	0.011
Cyclohexyl-isocyanide hydratase inhibitor	0.710	0.005
Arginine 2-monooxygenase inhibitor	0.721	0.017
Omptin inhibitor	0.721	0.018
Acetylesterase inhibitor	0.713	0.011
Polyneuridine-aldehyde esterase inhibitor	0.707	0.004
Methylamine-glutamate N-methyltransferase inhibitor	0.711	0.010
Glucan endo-1.6-beta-glucosidase inhibitor	0.717	0.017
Naphthalene 1.2-dioxygenase inhibitor	0.706	0.006

Ferredoxin-NAD+ reductase inhibitor	0.706	0.006
Alkane 1-monooxygenase inhibitor	0.712	0.015
Thioredoxin inhibitor	0.706	0.010
Macrophage colony stimulating factor agonist	0.707	0.013
3-Hydroxybenzoate 6-monooxygenase inhibitor	0.705	0.010
Nicotinic alpha6beta3beta4alpha5 receptor antagonist	0.721	0.029
Taurine dehydrogenase inhibitor	0.718	0.028
CYP2B substrate	0.701	0.011
NADPH peroxidase inhibitor	0.711	0.025
CYP2C12 substrate	0.703	0.057

Table S10. Prediction of the selected activity (Pa > 0.7) of molecule (**4a**) using PASS software. The results are expressed as probability to be active (Pa) or inactive (Pi).

	**Pa**	**Pi**
Antineoplastic (breast cancer)	0.938	0.003
Antineoplastic (lung cancer)	0.929	0.003
Antineoplastic	0.879	0.005
Epidermal growth factor antagonist	0.870	0.002
Phosphatidylcholine-retinol O-acyltransferase inhibitor	0.868	0.004
Aspulvinone dimethylallyltransferase inhibitor	0.875	0.014
Beta-adrenergic receptor kinase inhibitor	0.860	0.007
G-protein-coupled receptor kinase inhibitor	0.860	0.007
Fatty-acyl-CoA synthase inhibitor	0.826	0.004
Antineoplastic (colorectal cancer)	0.810	0.004
Growth factor antagonist	0.808	0.004
Antineoplastic (colon cancer)	0.807	0.004
Epidermal growth factor receptor kinase inhibitor	0.805	0.003
Chloride peroxidase inhibitor	0.791	0.004
Phobic disorders treatment	0.809	0.030
Membrane permeability inhibitor	0.788	0.012
Acetylesterase inhibitor	0.764	0.007
Carminative	0.758	0.005
Cl--transporting ATPase inhibitor	0.760	0.009
Cutinase inhibitor	0.758	0.007
CYP2E1 substrate	0.752	0.005
CYP2E substrate	0.748	0.006
Ubiquinol-cytochrome-c reductase inhibitor	0.777	0.041
CYP2J substrate	0.764	0.030
Sugar-phosphatase inhibitor	0.748	0.025
Testosterone 17beta-dehydrogenase (NADP+) inhibitor	0.757	0.037
All-trans-retinyl-palmitate hydrolase inhibitor	0.718	0.009
NADH kinase inhibitor	0.716	0.006
Phosphoenolpyruvate-protein phosphotransferase inhibitor	0.711	0.003
Chlordecone reductase inhibitor	0.740	0.032
Glutamyl endopeptidase II inhibitor	0.722	0.023
Complement factor D inhibitor	0.714	0.017
NADPH peroxidase inhibitor	0.711	0.025
5-O-(4-coumaroyl)-D-quinate 3'-monooxygenase inhibitor	0.706	0.022
Mucomembranous protector	0.726	0.045
Acrocylindropepsin inhibitor	0.718	0.040
Chymosin inhibitor	0.718	0.040
Saccharopepsin inhibitor	0.718	0.040
Glycosylphosphatidylinositol phospholipase D inhibitor	0.710	0.033

Table S11. Prediction of the selected activity (Pa > 0.7) of molecule (**4b**) using PASS software. The results are expressed as probability to be active (Pa) or inactive (Pi).

	**Pa**	**Pi**
Epidermal growth factor antagonist	0.980	0.001
Growth factor antagonist	0.946	0.003
Epidermal growth factor receptor kinase inhibitor	0.936	0.003
Antineoplastic (breast cancer)	0.936	0.003
Antineoplastic (lung cancer)	0.928	0.003
Phosphatidylcholine-retinol O-acyltransferase inhibitor	0.921	0.002
Antineoplastic (colorectal cancer)	0.921	0.003
Antineoplastic (colon cancer)	0.919	0.003
Phobic disorders treatment	0.884	0.008
Atherosclerosis treatment	0.879	0.004
Aspulvinone dimethylallyltransferase inhibitor	0.877	0.013
Acetylesterase inhibitor	0.864	0.003
Cutinase inhibitor	0.862	0.004
Ubiquinol-cytochrome-c reductase inhibitor	0.868	0.012
CYP2J substrate	0.845	0.011
All-trans-retinyl-palmitate hydrolase inhibitor	0.834	0.004
Testosterone 17beta-dehydrogenase (NADP+) inhibitor	0.846	0.015
Sugar-phosphatase inhibitor	0.839	0.011
Antineoplastic	0.834	0.008
Antidiabetic (type 2)	0.824	0.004
NADPH peroxidase inhibitor	0.828	0.009
Complement factor D inhibitor	0.819	0.005
Glutamyl endopeptidase II inhibitor	0.819	0.008
Feruloyl esterase inhibitor	0.820	0.010
Glycosylphosphatidylinositol phospholipase D inhibitor	0.819	0.010
Chymosin inhibitor	0.821	0.016
Saccharopepsin inhibitor	0.821	0.016
Acrocylindropepsin inhibitor	0.821	0.016
5-O-(4-coumaroyl)-D-quinate 3'-monooxygenase inhibitor	0.806	0.007
Ribulose-phosphate 3-epimerase inhibitor	0.803	0.007
Nicotinic alpha6beta3beta4alpha5 receptor antagonist	0.802	0.011
Chloride peroxidase inhibitor	0.793	0.004
Dehydro-L-gulonate decarboxylase inhibitor	0.794	0.010
Fatty-acyl-CoA synthase inhibitor	0.786	0.005
Carboxypeptidase Taq inhibitor	0.788	0.008
UDP-N-acetylglucosamine 4-epimerase inhibitor	0.783	0.007
Bisphosphoglycerate phosphatase inhibitor	0.778	0.008
Fusarinine-C ornithinesterase inhibitor	0.772	0.010
Cl--transporting ATPase inhibitor	0.769	0.008
Phthalate 4.5-dioxygenase inhibitor	0.766	0.007
Membrane permeability inhibitor	0.771	0.015
Electron-transferring-flavoprotein dehydrogenase inhibitor	0.761	0.006
CYP2J2 substrate	0.772	0.018
Nicotinic alpha2beta2 receptor antagonist	0.767	0.015

Alkylacetylglycerophosphatase inhibitor	0.764	0.013
L-glutamate oxidase inhibitor	0.756	0.006
Acylcarnitine hydrolase inhibitor	0.766	0.017
Phospholipid-translocating ATPase inhibitor	0.756	0.008
Polyamine-transporting ATPase inhibitor	0.756	0.007
Chenodeoxycholoyltaurine hydrolase inhibitor	0.748	0.005
Gluconate 5-dehydrogenase inhibitor	0.749	0.007
IgA-specific serine endopeptidase inhibitor	0.750	0.010
Pullulanase inhibitor	0.750	0.012
2-Hydroxymuconate-semialdehyde hydrolase inhibitor	0.747	0.010
Glucan 1.4-alpha-maltotriohydrolase inhibitor	0.742	0.006
NADPH-cytochrome-c2 reductase inhibitor	0.747	0.011
Glucan endo-1.6-beta-glucosidase inhibitor	0.749	0.013
Glutathione thiolesterase inhibitor	0.747	0.012
Aminocarboxymuconate-semialdehyde decarboxylase inhibitor	0.738	0.004
Arylmalonate decarboxylase inhibitor	0.737	0.004
Pterin deaminase inhibitor	0.740	0.008
Alkenylglycerophosphocholine hydrolase inhibitor	0.756	0.024
G-protein-coupled receptor kinase inhibitor	0.748	0.018
Beta-adrenergic receptor kinase inhibitor	0.748	0.018
Crotonoyl-[acyl-carrier-protein] hydratase inhibitor	0.733	0.004
Lysostaphin inhibitor	0.734	0.006
Creatininase inhibitor	0.737	0.010
Dimethylargininase inhibitor	0.734	0.011
Allyl-alcohol dehydrogenase inhibitor	0.728	0.005
Thioredoxin inhibitor	0.728	0.008
Venombin AB inhibitor	0.731	0.012
Chlordecone reductase inhibitor	0.747	0.030
Methylamine-glutamate N-methyltransferase inhibitor	0.724	0.009
Arginine 2-monooxygenase inhibitor	0.730	0.016
3-Hydroxybenzoate 6-monooxygenase inhibitor	0.723	0.009
NADH kinase inhibitor	0.719	0.006
Macrophage colony stimulating factor agonist	0.724	0.011
Membrane integrity agonist	0.756	0.045
Glutamine-phenylpyruvate transaminase inhibitor	0.720	0.009
Omptin inhibitor	0.727	0.017
Pro-opiomelanocortin converting enzyme inhibitor	0.733	0.023
Polyneuridine-aldehyde esterase inhibitor	0.712	0.004
2-Haloacid dehalogenase inhibitor	0.712	0.004
Limulus clotting factor B inhibitor	0.718	0.011
tRNA-pseudouridine synthase I inhibitor	0.713	0.007
Cyclohexyl-isocyanide hydratase inhibitor	0.709	0.005
Glucan 1.4-alpha-maltotetraohydrolase inhibitor	0.706	0.005
Taurine dehydrogenase inhibitor	0.725	0.026
(S)-6-hydroxynicotine oxidase inhibitor	0.705	0.006
Naphthalene 1.2-dioxygenase inhibitor	0.704	0.006
Ferredoxin-NAD+ reductase inhibitor	0.704	0.006
Exoribonuclease II inhibitor	0.713	0.015

Fragilysin inhibitor	0.712	0.016
Alkane 1-monooxygenase inhibitor	0.711	0.015
S-alkylcysteine lyase inhibitor	0.702	0.007
Polyporopepsin inhibitor	0.724	0.034
JAK2 expression inhibitor	0.701	0.017
Pseudolysin inhibitor	0.702	0.019
CYP2C12 substrate	0.713	0.055

Table S12. Prediction of the selected activity (Pa > 0.7) of molecule (**4c**) using PASS software. The results are expressed as probability to be active (Pa) or inactive (Pi).

	Pa	Pi
Aspulvinone dimethylallyltransferase inhibitor	0.942	0.003
Atherosclerosis treatment	0.927	0.003
Vitamin D-like	0.919	0.000
Cl--transporting ATPase inhibitor	0.881	0.003
Phobic disorders treatment	0.885	0.008
Membrane permeability inhibitor	0.874	0.004
Phosphatidylcholine-retinol O-acyltransferase inhibitor	0.871	0.004
Chlordecone reductase inhibitor	0.875	0.008
Acetylesterase inhibitor	0.855	0.004
Chloride peroxidase inhibitor	0.845	0.003
Cutinase inhibitor	0.841	0.004
Testosterone 17beta-dehydrogenase (NADP+) inhibitor	0.846	0.015
Lipoprotein disorders treatment	0.835	0.004
Sugar-phosphatase inhibitor	0.840	0.011
Fatty-acyl-CoA synthase inhibitor	0.823	0.004
Complement factor D inhibitor	0.820	0.005
NADH kinase inhibitor	0.817	0.003
Glutamyl endopeptidase II inhibitor	0.820	0.008
Glycosylphosphatidylinositol phospholipase D inhibitor	0.812	0.011
NADPH peroxidase inhibitor	0.805	0.011
5-O-(4-coumaroyl)-D-quinate 3'-monooxygenase inhibitor	0.801	0.008
Ribulose-phosphate 3-epimerase inhibitor	0.795	0.008
Nicotinic alpha6beta3beta4alpha5 receptor antagonist	0.798	0.011
Dehydro-L-gulonate decarboxylase inhibitor	0.795	0.010
Acrocylindropepsin inhibitor	0.804	0.019
Saccharopepsin inhibitor	0.804	0.019
Chymosin inhibitor	0.804	0.019
Gluconate 2-dehydrogenase (acceptor) inhibitor	0.798	0.017
Carboxypeptidase Taq inhibitor	0.789	0.008
5 Hydroxytryptamine release stimulant	0.787	0.015
UDP-N-acetylglucosamine 4-epimerase inhibitor	0.773	0.008
Bisphosphoglycerate phosphatase inhibitor	0.769	0.008
Carminative	0.763	0.004
Phthalate 4.5-dioxygenase inhibitor	0.763	0.007
GST A substrate	0.769	0.013
Ubiquinol-cytochrome-c reductase inhibitor	0.790	0.036
Feruloyl esterase inhibitor	0.768	0.015
Fusarinine-C ornithinesterase inhibitor	0.761	0.012
Acylcarnitine hydrolase inhibitor	0.766	0.017
6-Pyruvoyltetrahydropterin synthase inhibitor	0.751	0.003
Electron-transferring-flavoprotein dehydrogenase inhibitor	0.750	0.007
L-glutamate oxidase inhibitor	0.749	0.007
Pullulanase inhibitor	0.752	0.012
4-Nitrophenol 2-monooxygenase inhibitor	0.745	0.005

Nicotinic alpha2beta2 receptor antagonist	0.756	0.016
Glucan endo-1.6-beta-glucosidase inhibitor	0.750	0.013
Polyamine-transporting ATPase inhibitor	0.745	0.008
Glucan 1.4-alpha-maltotriohydrolase inhibitor	0.742	0.006
IgA-specific serine endopeptidase inhibitor	0.745	0.010
CYP2J substrate	0.765	0.030
Alkenylglycerophosphocholine hydrolase inhibitor	0.757	0.024
Pterin deaminase inhibitor	0.741	0.008
Phospholipid-translocating ATPase inhibitor	0.741	0.009
2-Hydroxymuconate-semialdehyde hydrolase inhibitor	0.742	0.010
Gluconate 5-dehydrogenase inhibitor	0.739	0.008
GST P substrate	0.735	0.004
G-protein-coupled receptor kinase inhibitor	0.748	0.018
Beta-adrenergic receptor kinase inhibitor	0.748	0.018
Lysostaphin inhibitor	0.735	0.006
Glutathione thiolesterase inhibitor	0.741	0.013
NADPH-cytochrome-c2 reductase inhibitor	0.740	0.012
Arylmalonate decarboxylase inhibitor	0.731	0.005
Creatininase inhibitor	0.734	0.010
Chenodeoxycholoyltaurine hydrolase inhibitor	0.729	0.006
Alkylacetylglycerophosphatase inhibitor	0.738	0.015
Aldehyde dehydrogenase (pyrroloquinoline-quinone) inhibitor	0.727	0.005
Thioredoxin inhibitor	0.729	0.008
Venombin AB inhibitor	0.733	0.012
GST P1-1 substrate	0.722	0.003
Arginine 2-monooxygenase inhibitor	0.732	0.016
3-Hydroxybenzoate 6-monooxygenase inhibitor	0.725	0.009
Glutamine-phenylpyruvate transaminase inhibitor	0.721	0.009
Omptin inhibitor	0.728	0.016
Dimethylargininase inhibitor	0.722	0.012
Crotonoyl-[acyl-carrier-protein] hydratase inhibitor	0.713	0.005
Limulus clotting factor B inhibitor	0.719	0.011
tRNA-pseudouridine synthase I inhibitor	0.714	0.006
Mucomembranous protector	0.742	0.038
All-trans-retinyl-palmitate hydrolase inhibitor	0.712	0.009
Glucan 1.4-alpha-maltotetraohydrolase inhibitor	0.707	0.004
Methylamine-glutamate N-methyltransferase inhibitor	0.712	0.010
Exoribonuclease II inhibitor	0.714	0.015
Cyclohexyl-isocyanide hydratase inhibitor	0.703	0.005
Fragilysin inhibitor	0.714	0.016
Alkane 1-monooxygenase inhibitor	0.712	0.015
Taurine dehydrogenase inhibitor	0.724	0.026
Polyneuridine-aldehyde esterase inhibitor	0.701	0.004
Pro-opiomelanocortin converting enzyme inhibitor	0.720	0.025
CYP2J2 substrate	0.718	0.029
Pseudolysin inhibitor	0.703	0.019
Polyporopepsin inhibitor	0.715	0.036
CYP2C12 substrate	0.716	0.054

Table S13. Prediction of the selected activity (Pa > 0.7) of molecule (**4d**) using PASS software. The results are expressed as probability to be active (Pa) or inactive (Pi).

	Pa	Pi
Vitamin D-like	0.966	0.000
Atherosclerosis treatment	0.949	0.003
Lipoprotein disorders treatment	0.911	0.003
Phosphatidylcholine-retinol O-acyltransferase inhibitor	0.872	0.003
Aspulvinone dimethylallyltransferase inhibitor	0.879	0.013
G-protein-coupled receptor kinase inhibitor	0.857	0.008
Beta-adrenergic receptor kinase inhibitor	0.857	0.008
Fatty-acyl-CoA synthase inhibitor	0.823	0.004
Phobic disorders treatment	0.810	0.030
Membrane permeability inhibitor	0.787	0.012
CYP2E1 substrate	0.769	0.005
Chloride peroxidase inhibitor	0.767	0.005
Carminative	0.766	0.004
CYP2E substrate	0.765	0.005
Acetylesterase inhibitor	0.749	0.008
CYP2J substrate	0.767	0.029
Ubiquinol-cytochrome-c reductase inhibitor	0.773	0.042
Cl--transporting ATPase inhibitor	0.738	0.011
Testosterone 17beta-dehydrogenase (NADP+) inhibitor	0.761	0.036
Sugar-phosphatase inhibitor	0.746	0.025
Chlordecone reductase inhibitor	0.748	0.030
All-trans-retinyl-palmitate hydrolase inhibitor	0.721	0.009
Cutinase inhibitor	0.717	0.009
CYP2A6 substrate	0.714	0.009
NADH kinase inhibitor	0.712	0.006
Glutamyl endopeptidase II inhibitor	0.725	0.022
Complement factor D inhibitor	0.717	0.016
Mucomembranous protector	0.727	0.045
5-O-(4-coumaroyl)-D-quinate 3'-monooxygenase inhibitor	0.703	0.022
Glycosylphosphatidylinositol phospholipase D inhibitor	0.709	0.033

Table S14. Prediction of the selected activity (Pa > 0.7) of molecule (**5a**) using PASS software. The results are expressed as probability to be active (Pa) or inactive (Pi).

	**Pa**	**Pi**
Acrocylindropepsin inhibitor	0.858	0.010
Saccharopepsin inhibitor	0.858	0.010
Chymosin inhibitor	0.858	0.010
Aspulvinone dimethylallyltransferase inhibitor	0.859	0.018
Fatty-acyl-CoA synthase inhibitor	0.803	0.004
Chloride peroxidase inhibitor	0.801	0.004
Arachidonate-CoA ligase inhibitor	0.764	0.000
Mucomembranous protector	0.779	0.024
Ubiquinol-cytochrome-c reductase inhibitor	0.786	0.038
Polyporopepsin inhibitor	0.768	0.026
Glucan endo-1.6-beta-glucosidase inhibitor	0.741	0.014
Fusarinine-C ornithinesterase inhibitor	0.739	0.015
Aldehyde dehydrogenase (pyrroloquinoline-quinone) inhibitor	0.727	0.005
Bisphosphoglycerate phosphatase inhibitor	0.731	0.011
GST A substrate	0.736	0.018
Carminative	0.723	0.005
L-glutamate oxidase inhibitor	0.722	0.009
Eye irritation. inactive	0.717	0.005
Cl--transporting ATPase inhibitor	0.722	0.012
Phospholipid-translocating ATPase inhibitor	0.719	0.011
G-protein-coupled receptor kinase inhibitor	0.720	0.021
Beta-adrenergic receptor kinase inhibitor	0.720	0.021
Glycosylphosphatidylinositol phospholipase D inhibitor	0.726	0.029
Sugar-phosphatase inhibitor	0.719	0.030
Testosterone 17beta-dehydrogenase (NADP+) inhibitor	0.729	0.046
Nicotinic alpha6beta3beta4alpha5 receptor antagonist	0.706	0.033
Chlordecone reductase inhibitor	0.705	0.039

Table S15. Prediction of the selected activity (Pa > 0.7) of molecule (**5b**) using PASS software. The results are expressed as probability to be active (Pa) or inactive (Pi).

	**Pa**	**Pi**
Saccharopepsin inhibitor	0.912	0.004
Acrocylindropepsin inhibitor	0.912	0.004
Chymosin inhibitor	0.912	0.004
Arachidonate-CoA ligase inhibitor	0.899	0.000
Ubiquinol-cytochrome-c reductase inhibitor	0.873	0.011
Polyporopepsin inhibitor	0.864	0.010
Aspulvinone dimethylallyltransferase inhibitor	0.861	0.017
Glucan endo-1,6-beta-glucosidase inhibitor	0.851	0.004
Bisphosphoglycerate phosphatase inhibitor	0.844	0.004
Interleukin 8 antagonist	0.836	0.003
Glycosylphosphatidylinositol phospholipase D inhibitor	0.829	0.009
Fusarinine-C ornithinesterase inhibitor	0.828	0.005
Testosterone 17beta-dehydrogenase (NADP+) inhibitor	0.827	0.019
Pancreatic disorders treatment	0.826	0.002
Arylacetonitrilase inhibitor	0.824	0.008
L-glutamate oxidase inhibitor	0.820	0.004
Phospholipid-translocating ATPase inhibitor	0.820	0.004
Sugar-phosphatase inhibitor	0.819	0.013
Feruloyl esterase inhibitor	0.815	0.010
Nicotinic alpha6beta3beta4alpha5 receptor antagonist	0.814	0.009
Phobic disorders treatment	0.811	0.030
Cutinase inhibitor	0.808	0.005
Dehydro-L-gulonate decarboxylase inhibitor	0.804	0.009
NADPH peroxidase inhibitor	0.804	0.012
Chloride peroxidase inhibitor	0.803	0.004
Pro-opiomelanocortin converting enzyme inhibitor	0.803	0.014
Carboxypeptidase Taq inhibitor	0.802	0.007
Glutamyl endopeptidase II inhibitor	0.798	0.010
Complement factor D inhibitor	0.796	0.007
Arylalkyl acylamidase inhibitor	0.795	0.004
CYP2J substrate	0.788	0.024
Poly(alpha-L-guluronate) lyase inhibitor	0.785	0.005
5-O-(4-coumaroyl)-D-quinate 3'-monooxygenase inhibitor	0.784	0.010
Phosphatidylcholine-retinol O-acyltransferase inhibitor	0.784	0.007
Taurine dehydrogenase inhibitor	0.784	0.015
Nicotinic alpha2beta2 receptor antagonist	0.780	0.013
Phthalate 4,5-dioxygenase inhibitor	0.780	0.005
Ribulose-phosphate 3-epimerase inhibitor	0.779	0.009
(R)-6-hydroxynicotine oxidase inhibitor	0.777	0.004
Bothrolysin inhibitor	0.768	0.004
Electron-transferring-flavoprotein dehydrogenase inhibitor	0.766	0.005
Chenodeoxycholoyltaurine hydrolase inhibitor	0.765	0.005
Pullulanase inhibitor	0.761	0.011
Fatty-acyl-CoA synthase inhibitor	0.760	0.007

Glutathione thiolesterase inhibitor	0.760	0.011
UDP-N-acetylglucosamine 4-epimerase inhibitor	0.755	0.010
Aldehyde dehydrogenase (pyrroloquinoline-quinone) inhibitor	0.754	0.005
All-trans-retinyl-palmitate hydrolase inhibitor	0.754	0.006
GST A substrate	0.754	0.015
Lysostaphin inhibitor	0.752	0.005
Creatininase inhibitor	0.751	0.009
Pterin deaminase inhibitor	0.747	0.007
CYP2J2 substrate	0.746	0.023
Mucomembranous protector	0.744	0.037
N-acylmannosamine kinase inhibitor	0.743	0.005
Acylcarnitine hydrolase inhibitor	0.738	0.021
Phosphatidylserine decarboxylase inhibitor	0.738	0.008
Spermidine dehydrogenase inhibitor	0.738	0.007
Urethanase inhibitor	0.738	0.006
Allyl-alcohol dehydrogenase inhibitor	0.737	0.005
Thioredoxin inhibitor	0.737	0.007
Carnitinamidase inhibitor	0.736	0.006
Cl--transporting ATPase inhibitor	0.734	0.011
Alkylacetylglycerophosphatase inhibitor	0.733	0.016
Glutamine-phenylpyruvate transaminase inhibitor	0.733	0.008
Polyneuridine-aldehyde esterase inhibitor	0.732	0.004
tRNA-pseudouridine synthase I inhibitor	0.732	0.005
Superoxide dismutase inhibitor	0.727	0.011
Mucinaminylserine mucinaminidase inhibitor	0.726	0.009
Polyamine-transporting ATPase inhibitor	0.726	0.010
Fragilysin inhibitor	0.725	0.014
Ferredoxin-NAD+ reductase inhibitor	0.724	0.005
Naphthalene 1,2-dioxygenase inhibitor	0.724	0.005
(S)-6-hydroxynicotine oxidase inhibitor	0.722	0.005
Alkane 1-monooxygenase inhibitor	0.721	0.014
Arylsulfate sulfotransferase inhibitor	0.721	0.013
Gluconate 5-dehydrogenase inhibitor	0.721	0.009
4-Hydroxyproline epimerase inhibitor	0.718	0.006
Lysase inhibitor	0.718	0.020
NADPH-cytochrome-c2 reductase inhibitor	0.718	0.015
Pseudolysin inhibitor	0.717	0.017
Alkenylglycerophosphocholine hydrolase inhibitor	0.715	0.029
Glucan 1,4-alpha-maltotriohydrolase inhibitor	0.715	0.008
Leucolysin inhibitor	0.715	0.005
Chlordecone reductase inhibitor	0.713	0.037
IgA-specific serine endopeptidase inhibitor	0.709	0.013
Dimethylargininase inhibitor	0.702	0.014
Omptin inhibitor	0.702	0.021

Table S16. Prediction of the selected activity (Pa > 0.7) of molecule (**5c**) using PASS software. The results are expressed as probability to be active (Pa) or inactive (Pi).

	**Pa**	**Pi**
Aspulvinone dimethylallyltransferase inhibitor	0.913	0.005
Integrin alphaVbeta3 antagonist	0.893	0.000
Saccharopepsin inhibitor	0.888	0.006
Chymosin inhibitor	0.888	0.006
Acrocylindropepsin inhibitor	0.888	0.006
Chloride peroxidase inhibitor	0.860	0.003
Ubiquinol-cytochrome-c reductase inhibitor	0.865	0.013
Aldehyde dehydrogenase (pyrroloquinoline-quinone) inhibitor	0.856	0.003
Bisphosphoglycerate phosphatase inhibitor	0.853	0.004
Cl--transporting ATPase inhibitor	0.850	0.004
Antineoplastic (breast cancer)	0.842	0.004
Phospholipid-translocating ATPase inhibitor	0.838	0.004
Glucan endo-1.6-beta-glucosidase inhibitor	0.838	0.005
L-glutamate oxidase inhibitor	0.836	0.003
Fusarinine-C ornithinesterase inhibitor	0.836	0.005
NADPH peroxidase inhibitor	0.832	0.008
Antibiotic Glycopeptide-like	0.825	0.002
Arylacetonitrilase inhibitor	0.829	0.008
Cutinase inhibitor	0.823	0.004
Chlordecone reductase inhibitor	0.827	0.015
Glycosylphosphatidylinositol phospholipase D inhibitor	0.819	0.010
Sugar-phosphatase inhibitor	0.822	0.013
Polyporopepsin inhibitor	0.823	0.016
Testosterone 17beta-dehydrogenase (NADP+) inhibitor	0.825	0.019
Poly(alpha-L-guluronate) lyase inhibitor	0.810	0.004
GST A substrate	0.813	0.008
Phobic disorders treatment	0.825	0.025
Fatty-acyl-CoA synthase inhibitor	0.803	0.004
5-O-(4-coumaroyl)-D-quinate 3'-monooxygenase inhibitor	0.801	0.007
NADH kinase inhibitor	0.796	0.004
Gluconate 2-dehydrogenase (acceptor) inhibitor	0.804	0.015
Complement factor D inhibitor	0.794	0.007
Glutamyl endopeptidase II inhibitor	0.796	0.010
Carboxypeptidase Taq inhibitor	0.793	0.008
Dehydro-L-gulonate decarboxylase inhibitor	0.791	0.010
Arylalkyl acylamidase inhibitor	0.785	0.004
Taurine dehydrogenase inhibitor	0.784	0.016
Prolyl aminopeptidase inhibitor	0.777	0.009
Spermidine dehydrogenase inhibitor	0.771	0.005
Mucomembranous protector	0.788	0.022
Phthalate 4.5-dioxygenase inhibitor	0.771	0.006
Ribulose-phosphate 3-epimerase inhibitor	0.773	0.010
(R)-6-hydroxynicotine oxidase inhibitor	0.764	0.004
N-acylmannosamine kinase inhibitor	0.762	0.004

Nicotinic alpha6beta3beta4alpha5 receptor antagonist	0.772	0.016
Chemoprotective	0.757	0.003
Bothrolysin inhibitor	0.757	0.004
GST P substrate	0.753	0.003
Acylcarnitine hydrolase inhibitor	0.766	0.017
Pterin deaminase inhibitor	0.754	0.007
Mucinaminylserine mucinaminidase inhibitor	0.747	0.008
IgA-specific serine endopeptidase inhibitor	0.748	0.010
UDP-N-acetylglucosamine 4-epimerase inhibitor	0.748	0.010
Arylsulfate sulfotransferase inhibitor	0.747	0.011
Glucan 1.4-alpha-maltotriohydrolase inhibitor	0.742	0.006
Nicotinic alpha2beta2 receptor antagonist	0.753	0.017
Lysostaphin inhibitor	0.741	0.006
GST P1-1 substrate	0.732	0.003
Creatininase inhibitor	0.738	0.010
Thioredoxin inhibitor	0.734	0.007
Pullulanase inhibitor	0.738	0.013
Alkylacetylglycerophosphatase inhibitor	0.738	0.015
Glutathione thiolesterase inhibitor	0.735	0.014
CYP2J substrate	0.754	0.033
Membrane permeability inhibitor	0.743	0.023
Aryldialkylphosphatase inhibitor	0.723	0.004
Carnitinamidase inhibitor	0.724	0.006
Electron-transferring-flavoprotein dehydrogenase inhibitor	0.727	0.009
Phosphatidylserine decarboxylase inhibitor	0.725	0.009
Carminative	0.720	0.006
Superoxide dismutase inhibitor	0.724	0.011
Urethanase inhibitor	0.720	0.007
Hyponitrite reductase inhibitor	0.717	0.005
Camphor 1.2-monooxygenase inhibitor	0.716	0.004
4-Nitrophenol 2-monooxygenase inhibitor	0.717	0.007
CYP2F1 substrate	0.714	0.005
6-Pyruvoyltetrahydropterin synthase inhibitor	0.711	0.004
Polyamine-transporting ATPase inhibitor	0.718	0.011
tRNA-pseudouridine synthase I inhibitor	0.712	0.007
Phosphatidylcholine-retinol O-acyltransferase inhibitor	0.717	0.013
Gluconate 5-dehydrogenase inhibitor	0.713	0.010
Anthranilate-CoA ligase inhibitor	0.706	0.005
Feruloyl esterase inhibitor	0.718	0.020
3-Carboxyethylcatechol 2.3-dioxygenase inhibitor	0.703	0.004
Prostaglandin-A1 DELTA-isomerase inhibitor	0.703	0.005
(S)-6-hydroxynicotine oxidase inhibitor	0.704	0.006
Fragilysin inhibitor	0.713	0.016
Cyclohexanone monooxygenase inhibitor	0.701	0.005
S-alkylcysteine lyase inhibitor	0.701	0.007
Trimethylamine-oxide aldolase inhibitor	0.701	0.008
Aspartate-phenylpyruvate transaminase inhibitor	0.702	0.009
NADPH-cytochrome-c2 reductase inhibitor	0.707	0.016

Pseudolysin inhibitor	0.708	0.018
Lysase inhibitor	0.708	0.022
Omptin inhibitor	0.701	0.021
CYP2J2 substrate	0.709	0.031
Alkenylglycerophosphocholine hydrolase inhibitor	0.708	0.030

Table S17. Prediction of the selected activity (Pa > 0.7) of molecule (**5d**) using PASS software. The results are expressed as probability to be active (Pa) or inactive (Pi).

	Pa	Pi
Antineoplastic (breast cancer)	0.934	0.003
Aspulvinone dimethylallyltransferase inhibitor	0.889	0.010
Antineoplastic	0.869	0.005
Fatty-acyl-CoA synthase inhibitor	0.846	0.003
Chymosin inhibitor	0.836	0.014
Saccharopepsin inhibitor	0.836	0.014
Acrocylindropepsin inhibitor	0.836	0.014
Ubiquinol-cytochrome-c reductase inhibitor	0.835	0.021
Mucomembranous protector	0.824	0.013
Chloride peroxidase inhibitor	0.813	0.004
Bisphosphoglycerate phosphatase inhibitor	0.797	0.006
Cl--transporting ATPase inhibitor	0.791	0.007
Carminative	0.788	0.004
Beta-adrenergic receptor kinase inhibitor	0.793	0.014
G-protein-coupled receptor kinase inhibitor	0.793	0.014
GST A substrate	0.789	0.011
Fusarinine-C ornithinesterase inhibitor	0.787	0.009
L-glutamate oxidase inhibitor	0.776	0.005
Complement factor D inhibitor	0.778	0.009
Phospholipid-translocating ATPase inhibitor	0.773	0.006
Aldehyde dehydrogenase (pyrroloquinoline-quinone) inhibitor	0.761	0.005
Sugar-phosphatase inhibitor	0.774	0.020
Testosterone 17beta-dehydrogenase (NADP+) inhibitor	0.783	0.030
Glucan endo-1,6-beta-glucosidase inhibitor	0.764	0.011
Chlordecone reductase inhibitor	0.770	0.026
Phosphoenolpyruvate-protein phosphotransferase inhibitor	0.747	0.003
NADH kinase inhibitor	0.748	0.005
Cutinase inhibitor	0.743	0.008
Glutamyl endopeptidase II inhibitor	0.749	0.018
NADPH peroxidase inhibitor	0.746	0.020
Glycosylphosphatidylinositol phospholipase D inhibitor	0.742	0.025
5-O-(4-coumaroyl)-D-quinate 3'-monooxygenase inhibitor	0.734	0.017
Polyporopepsin inhibitor	0.742	0.030
Ribulose-phosphate 3-epimerase inhibitor	0.725	0.016
Membrane permeability inhibitor	0.734	0.026
Phosphatidylcholine-retinol O-acyltransferase inhibitor	0.719	0.013
Phobic disorders treatment	0.756	0.051
Poly(alpha-L-guluronate) lyase inhibitor	0.711	0.008
CYP2J substrate	0.739	0.038
GST P substrate	0.704	0.004
Antieczematic	0.733	0.035
Nicotinic alpha6beta3beta4alpha5 receptor antagonist	0.720	0.029
Feruloyl esterase inhibitor	0.709	0.021
Dehydro-L-gulonate decarboxylase inhibitor	0.701	0.022

Table S18. Prediction of the selected activity (Pa > 0.7) of molecule (**6b**) using PASS software. The results are expressed as probability to be active (Pa) or inactive (Pi).

	**Pa**	**Pi**
Chymosin inhibitor	0.889	0.006
Acrocylindropepsin inhibitor	0.889	0.006
Saccharopepsin inhibitor	0.889	0.006
Ubiquinol-cytochrome-c reductase inhibitor	0.861	0.014
Arylacetonitrilase inhibitor	0.849	0.006
Fusarinine-C ornithinesterase inhibitor	0.844	0.004
Phobic disorders treatment	0.849	0.017
NADPH peroxidase inhibitor	0.834	0.008
Complement factor D inhibitor	0.814	0.005
Arachidonate-CoA ligase inhibitor	0.808	0.000
Polyporopepsin inhibitor	0.823	0.016
Pro-opiomelanocortin converting enzyme inhibitor	0.815	0.012
Glutamine-phenylpyruvate transaminase inhibitor	0.780	0.005
Superoxide dismutase inhibitor	0.779	0.008
L-glutamate oxidase inhibitor	0.775	0.005
Polyamine-transporting ATPase inhibitor	0.775	0.006
Fatty-acyl-CoA synthase inhibitor	0.772	0.006
Aspulvinone dimethylallyltransferase inhibitor	0.799	0.034
Glucan endo-1,6-beta-glucosidase inhibitor	0.774	0.010
Chloride peroxidase inhibitor	0.767	0.005
Acylcarnitine hydrolase inhibitor	0.775	0.016
Bisphosphoglycerate phosphatase inhibitor	0.767	0.008
Phospholipid-translocating ATPase inhibitor	0.765	0.007
Pancreatic disorders treatment	0.760	0.002
Phosphatidylserine decarboxylase inhibitor	0.761	0.005
Nicotinic alpha6beta3beta4alpha5 receptor antagonist	0.770	0.017
Testosterone 17beta-dehydrogenase (NADP+) inhibitor	0.781	0.030
Mucinaminylserine mucinaminidase inhibitor	0.755	0.007
Glucose oxidase inhibitor	0.762	0.016
Albendazole monooxygenase inhibitor	0.745	0.003
Venombin AB inhibitor	0.747	0.011
Omptin inhibitor	0.749	0.013
Glycosylphosphatidylinositol phospholipase D inhibitor	0.756	0.022
GST A substrate	0.750	0.016
Pterin deaminase inhibitor	0.740	0.008
Leucolysin inhibitor	0.735	0.004
NADPH-cytochrome-c2 reductase inhibitor	0.742	0.012
Limulus clotting factor B inhibitor	0.737	0.009
Macrophage colony stimulating factor agonist	0.737	0.009
UDP-N-acetylglucosamine 4-epimerase inhibitor	0.738	0.012
Dimethylargininase inhibitor	0.736	0.011
Glutamyl endopeptidase II inhibitor	0.743	0.019
Sugar-phosphatase inhibitor	0.748	0.025
S-alkylcysteine lyase inhibitor	0.729	0.006

Carnitinamidase inhibitor	0.725	0.006
Pseudolysin inhibitor	0.730	0.015
Phthalate 4,5-dioxygenase inhibitor	0.725	0.011
Mucomembranous protector	0.747	0.036
Fibrolase inhibitor	0.715	0.006
Exoribonuclease II inhibitor	0.719	0.015
Fragilysin inhibitor	0.719	0.015
(R)-6-hydroxynicotine oxidase inhibitor	0.707	0.007
Carboxypeptidase Taq inhibitor	0.716	0.017
Arylalkyl acylamidase inhibitor	0.705	0.006
CYP2J substrate	0.736	0.038
Arginine 2-monooxygenase inhibitor	0.715	0.018
Nicotinic alpha2beta2 receptor antagonist	0.720	0.023
Hydrogen dehydrogenase inhibitor	0.706	0.009
Threonine aldolase inhibitor	0.706	0.009
Dehydro-L-gulonate decarboxylase inhibitor	0.716	0.020
Preneoplastic conditions treatment	0.702	0.008
5-O-(4-coumaroyl)-D-quinate 3'-monooxygenase inhibitor	0.706	0.022
Feruloyl esterase inhibitor	0.702	0.021
Taurine dehydrogenase inhibitor	0.708	0.030
Antiseborrheic	0.711	0.036
CDP-glycerol glycerophosphotransferase inhibitor	0.710	0.051

Table S19. Prediction of the selected activity (Pa > 0.7) of molecule (**6c**) using PASS software. The results are expressed as probability to be active (Pa) or inactive (Pi).

	**Pa**	**Pi**
Aspulvinone dimethylallyltransferase inhibitor	0.858	0.018
NADPH peroxidase inhibitor	0.839	0.007
Saccharopepsin inhibitor	0.842	0.013
Chymosin inhibitor	0.842	0.013
Acrocylindropepsin inhibitor	0.842	0.013
Fusarinine-C ornithinesterase inhibitor	0.834	0.005
Phobic disorders treatment	0.841	0.019
Arylacetonitrilase inhibitor	0.823	0.009
Chloride peroxidase inhibitor	0.813	0.004
Ubiquinol-cytochrome-c reductase inhibitor	0.831	0.023
Fatty-acyl-CoA synthase inhibitor	0.790	0.005
Complement factor D inhibitor	0.789	0.008
GST A substrate	0.786	0.011
L-glutamate oxidase inhibitor	0.772	0.005
Cl--transporting ATPase inhibitor	0.772	0.008
Acylcarnitine hydrolase inhibitor	0.775	0.016
Antibiotic Glycopeptide-like	0.761	0.003
Phospholipid-translocating ATPase inhibitor	0.763	0.007
Aldehyde dehydrogenase (pyrroloquinoline-quinone) inhibitor	0.752	0.005
Bisphosphoglycerate phosphatase inhibitor	0.749	0.010
Mucinaminylserine mucinaminidase inhibitor	0.745	0.008
Mucomembranous protector	0.762	0.030
Polyamine-transporting ATPase inhibitor	0.739	0.009
S-alkylcysteine lyase inhibitor	0.727	0.006
Phosphatidylserine decarboxylase inhibitor	0.724	0.009
Testosterone 17beta-dehydrogenase (NADP+) inhibitor	0.753	0.038
Superoxide dismutase inhibitor	0.722	0.012
Polyporopepsin inhibitor	0.739	0.031
Omptin inhibitor	0.725	0.017
Pterin deaminase inhibitor	0.716	0.010
Glucan endo-1,6-beta-glucosidase inhibitor	0.718	0.017
Glucose oxidase inhibitor	0.722	0.021
Venombin AB inhibitor	0.710	0.015
Limulus clotting factor B inhibitor	0.707	0.012
Sugar-phosphatase inhibitor	0.722	0.029
Glutamine-phenylpyruvate transaminase inhibitor	0.702	0.010
Glutamyl endopeptidase II inhibitor	0.713	0.024
NADPH-cytochrome-c2 reductase inhibitor	0.701	0.017
Chlordecone reductase inhibitor	0.718	0.036
Glycosylphosphatidylinositol phospholipase D inhibitor	0.711	0.032

Table S20. Prediction of the selected activity (Pa > 0.7) of molecule (**6d**) using PASS software. The results are expressed as probability to be active (Pa) or inactive (Pi).

	Pa	Pi
Antineoplastic (breast cancer)	0.856	0.004
Fatty-acyl-CoA synthase inhibitor	0.835	0.004
Mycothiol-S-conjugate amidase inhibitor	0.827	0.003
Aspulvinone dimethylallyltransferase inhibitor	0.819	0.028
Mucomembranous protector	0.800	0.018
Fusarinine-C ornithinesterase inhibitor	0.784	0.009
Antineoplastic	0.788	0.013
G-protein-coupled receptor kinase inhibitor	0.783	0.014
Beta-adrenergic receptor kinase inhibitor	0.783	0.014
Complement factor D inhibitor	0.772	0.009
Ubiquinol-cytochrome-c reductase inhibitor	0.794	0.035
Chloride peroxidase inhibitor	0.753	0.005
GST A substrate	0.760	0.014
Acrocylindropepsin inhibitor	0.771	0.027
Saccharopepsin inhibitor	0.771	0.027
Chymosin inhibitor	0.771	0.027
NADPH peroxidase inhibitor	0.756	0.018
Phobic disorders treatment	0.778	0.042
Omptin inhibitor	0.713	0.019

Table S21. Cartesian coordinates of molecule (**1**) according to B3LYP/6-31G(d) level theory in gas phase.

	X	Y	Z
C	0.60178500	1.75126200	0.00000000
H	-0.32603400	2.31946700	0.00000000
H	1.52404700	2.32415700	0.00000000
C	0.60178500	0.41084200	0.00000000
H	1.55138800	-0.12559100	0.00000000
C	-0.60178500	-0.41084200	0.00000000
H	-1.55138800	0.12559100	0.00000000
C	-0.60178500	-1.75126200	0.00000000
H	0.32603400	-2.31946700	0.00000000
H	-1.52404700	-2.32415700	0.00000000

Table S22. Cartesian coordinates of molecule (**2a**) according to B3LYP/6-31G(d) level theory in gas phase.

	X	Y	Z
C	2.51497100	-0.22115500	0.00439300
H	2.60789100	-1.30502700	0.01076900
H	3.43979100	0.34722500	0.00018300
C	1.31578900	0.38457300	-0.00104300
H	1.28307500	1.47579300	-0.01140700
C	0.03007600	-0.28459800	0.00828500
H	0.02984100	-1.37553000	0.01409300
C	-1.14614100	0.37869800	0.00149300
H	-1.15443600	1.46754100	-0.00526600
N	-2.41171900	-0.18727100	-0.08414700
H	-2.45931900	-1.17629200	0.13262000
H	-3.15298300	0.33207700	0.36926100

Table S23. Cartesian coordinates of molecule (**2b**) according to B3LYP/6-31G(d) level theory in gas phase.

	X	Y	Z
C	1.81232500	-0.40433300	0.01054300
H	1.78962700	-1.49304400	0.01502300
C	0.66105900	0.29764900	0.01080700
H	0.71219800	1.38849100	0.00093700
C	-0.66105900	-0.29764900	0.01080700
H	-0.71219800	-1.38849100	0.00093700
C	-1.81232500	0.40433300	0.01054300
H	-1.78962700	1.49304400	0.01502300
N	-3.10556700	-0.12657600	-0.09844300
H	-3.15355000	-1.12273100	0.09165900
H	-3.79971700	0.36660500	0.45338400
N	3.10556700	0.12657600	-0.09844300
H	3.15355000	1.12273100	0.09165900
H	3.79971700	-0.36660500	0.45338400

Table S24. Cartesian coordinates of molecule (**2c**) according to B3LYP/6-31G(d) level theory in gas phase.

	X	Y	Z
C	-1.17940200	1.41833500	0.00001900
H	-0.49111600	2.25466200	0.00003600
H	-2.24626300	1.62758400	0.00001800
C	-0.72324100	0.15600300	0.00000200
C	0.72324100	-0.15600300	0.00000200
C	1.17940200	-1.41833500	0.00001900
H	0.49111600	-2.25466200	0.00003400
H	2.24626300	-1.62758400	0.00001700
N	-1.60176500	-1.00034000	-0.00002100
H	-2.21362300	-0.96242500	0.81337200
H	-2.21363700	-0.96238100	-0.81340200
N	1.60176500	1.00034000	-0.00002000
H	2.21363300	0.96241600	0.81336600
H	2.21362800	0.96239000	-0.81340800

Table S25. Cartesian coordinates of molecule (**2d**) according to B3LYP/6-31G(d) level theory in gas phase.

C	2.79514700	-0.17566300	0.02798800
H	3.01198700	-1.22192200	0.23187500
H	3.64755500	0.48599700	-0.08887500
C	1.53256500	0.27602900	-0.08065700
H	1.39965500	1.34004100	-0.28966400
C	0.33353800	-0.52536800	0.02767400
H	0.46559900	-1.60163000	0.12219600
C	-0.94279300	-0.05292700	0.01316500
N	-2.06573300	-0.87794700	-0.11829600
H	-1.84262800	-1.86637100	-0.12143100
H	-2.79692900	-0.66654400	0.55531000
N	-1.31542800	1.29008900	0.12643800
H	-0.53677500	1.93419300	0.17977000
H	-1.99107200	1.57881000	-0.57519500

Table S26. Cartesian coordinates of molecule (**3a**) according to B3LYP/6-31G(d) level theory in gas phase.

	X	Y	Z
C	-2.53053700	-0.26001300	0.00001100
H	-2.57467300	-1.34702800	0.00006300
H	-3.48059200	0.26563900	0.00011500
C	-1.36054400	0.39546600	-0.00003400
H	-1.36199900	1.48623000	0.00009600
C	-0.05304400	-0.24511100	0.00000700
H	-0.04499600	-1.33647000	-0.00019000
C	1.11448300	0.41908400	-0.00005800
H	1.09280600	1.51041600	-0.00002100
C	2.47198100	-0.21694200	0.00002700
H	3.05525200	0.08855500	-0.87972200
H	3.05482900	0.08784200	0.88036200
H	2.40534200	-1.31009400	-0.00041800

Table S27. Cartesian coordinates of molecule (**3b**) according to B3LYP/6-31G(d) level theory in gas phase.

	X	Y	Z
C	-1.80448400	-0.43313800	-0.00003900
H	-1.73254300	-1.52230200	-0.00011100
C	-0.66922100	0.28532700	0.00001200
H	-0.73319700	1.37513800	0.00005900
C	0.66922100	-0.28532700	0.00001200
H	0.73319700	-1.37513800	0.00005900
C	1.80448400	0.43313800	-0.00003900
H	1.73254300	1.52230200	-0.00011100
C	3.19011600	-0.14008500	0.00001500
H	3.75989100	0.19004700	-0.87990400
H	3.75967400	0.18970200	0.88023100
H	3.17287700	-1.23532000	-0.00020300
C	-3.19011600	0.14008500	0.00001500
H	-3.75989100	-0.19004700	-0.87990400
H	-3.75967400	-0.18970200	0.88023100
H	-3.17287700	1.23532000	-0.00020300

Table S28. Cartesian coordinates of molecule (**3c**) according to B3LYP/6-31G(d) level theory in gas phase.

	X	Y	Z
C	-1.02797700	1.53223800	0.00000100
H	-0.28459900	2.32212100	0.00000200
H	-2.06595900	1.85302400	0.00000100
C	-0.70566100	0.22711800	0.00000000
C	0.70566100	-0.22711800	0.00000000
C	1.02797700	-1.53223800	0.00000100
H	0.28459900	-2.32212100	0.00000300
H	2.06595900	-1.85302400	0.00000100
C	1.79838900	0.81904600	-0.00000100
H	2.78628200	0.34994400	0.00000000
H	1.73254000	1.46852500	-0.88175800
H	1.73254000	1.46852800	0.88175400
C	-1.79838900	-0.81904600	-0.00000100
H	-1.73254000	-1.46852500	-0.88175800
H	-1.73254000	-1.46852800	0.88175400
H	-2.78628200	-0.34994400	0.00000000

Table S29. Cartesian coordinates of molecule (**3d**) according to B3LYP/6-31G(d) level theory in gas phase.

	X	Y	Z
C	2.81737200	-0.21557200	-0.00006400
H	2.98087300	-1.29125300	-0.00016200
H	3.70388300	0.41141200	-0.00008700
C	1.58140000	0.30800400	0.00005800
H	1.47936000	1.39168000	0.00010800
C	0.36403800	-0.48983000	0.00008000
H	0.52421800	-1.56894300	0.00008700
C	-0.91260600	-0.05062400	0.00006300
C	-2.06098700	-1.02806900	-0.00003500
H	-2.70450700	-0.88261700	0.87956400
H	-2.70429100	-0.88277700	-0.87981500
H	-1.71556400	-2.06650300	0.00010500
C	-1.32279800	1.40019900	-0.00002800
H	-1.94237900	1.62596400	0.87922600
H	-0.47827800	2.09246600	0.00023000
H	-1.94182400	1.62591900	-0.87970400

Table S30. Cartesian coordinates of molecule (**4a**) according to B3LYP/6-31G(d) level theory in gas phase.

	X	Y	Z
C	3.63073100	0.27707300	0.00000100
H	3.59814100	1.36418800	0.00001600
H	4.61478700	-0.18137300	-0.00002000
C	2.51003100	-0.45852100	-0.00002500
H	2.57790600	-1.54593100	-0.00005400
C	1.17324600	0.11560200	0.00001600
H	1.10322000	1.20155200	0.00006000
C	0.04526800	-0.60841700	-0.00002800
H	0.05588200	-1.69531700	-0.00008000
C	-1.31937500	-0.00579800	-0.00004300
F	-2.02937700	-0.39806000	1.08535000
F	-1.29563900	1.34435300	0.00020100
F	-2.02935600	-0.39770900	-1.08549000

Table S31. Cartesian coordinates of molecule (**4b**) according to B3LYP/6-31G(d) level theory in gas phase.

	X	Y	Z
C	0.60735400	1.73890000	0.00013500
H	-0.31247900	2.31784200	0.00001600
C	0.60735400	0.39917000	0.00048000
H	1.55642900	-0.13194700	0.00029100
C	-0.60735400	-0.39917000	0.00048000
H	-1.55642900	0.13194700	0.00029100
C	-0.60735400	-1.73890000	0.00013500
H	0.31247900	-2.31784200	0.00001600
C	-1.85561500	-2.56075200	-0.00015800
C	1.85561500	2.56075200	-0.00015800
F	-1.90505500	-3.36650300	1.08547800
F	-2.97485600	-1.80733700	0.00070400
F	-1.90577400	-3.36514600	-1.08652100
F	2.97485600	1.80733700	0.00070400
F	1.90505500	3.36650300	1.08547800
F	1.90577400	3.36514600	-1.08652100

Table S32. Cartesian coordinates of molecule (**4c**) according to B3LYP/6-31G(d) level theory in gas phase.

	X	Y	Z
C	0.36976200	-1.83015300	0.00001300
H	-0.61090800	-2.28610100	0.00001900
H	1.22201100	-2.49796900	0.00001400
C	0.54495200	-0.50107800	0.00000400
C	-0.54495200	0.50107800	0.00000300
C	-0.36976200	1.83015300	0.00001300
H	0.61090800	2.28610100	0.00001900
H	-1.22201100	2.49796900	0.00001400
C	-1.96487900	-0.02981700	-0.00000200
C	1.96487900	0.02981700	-0.00000200
F	-2.88374100	0.95426500	0.00001800
F	-2.20821000	-0.79926700	-1.08718200
F	-2.20820400	-0.79930600	1.08715100
F	2.20820800	0.79927100	-1.08717900
F	2.20820500	0.79930100	1.08715400
F	2.88374100	-0.95426500	0.00001300

Table S33. Cartesian coordinates of molecule (**4d**) according to B3LYP/6-31G(d) level theory in gas phase.

	X	Y	Z
C	-3.55000900	-1.29223200	-0.00004700
H	-3.41876300	-2.37191300	-0.00018000
H	-4.57146400	-0.92477000	0.00024500
C	-2.50175500	-0.45477700	0.00003600
H	-2.66711900	0.61768100	0.00050200
C	-1.13338600	-0.94575500	-0.00001900
H	-1.01764100	-2.02646000	0.00004100
C	-0.00747800	-0.20762700	0.00001400
C	1.34797200	-0.86432100	0.00000000
C	-0.03398700	1.30340800	-0.00007600
F	-0.68477200	1.78623000	-1.08482700
F	1.20002800	1.83537900	0.00024700
F	-0.68529600	1.78626000	1.08450100
F	2.06869000	-0.51670100	-1.08725900
F	2.06890800	-0.51621800	1.08709100
F	1.24875800	-2.21125200	0.00024200

Table S34. Cartesian coordinates of molecule (**5a**) according to B3LYP/6-31G(d) level theory in gas phase.

	X	Y	Z
C	3.26196900	0.27419800	0.00001400
H	3.22790400	1.36095000	0.00006200
H	4.24583300	-0.18434000	-0.00000200
C	2.14063200	-0.46358700	-0.00002100
H	2.20663500	-1.55037300	-0.00006900
C	0.81555600	0.12341700	0.00000200
H	0.72678100	1.20716000	0.00004300
C	-0.31518400	-0.59818800	0.00000200
H	-0.38901000	-1.67745600	-0.00002700
N	-1.61539900	0.04482900	0.00002100
O	-1.67602200	1.27666000	-0.00003000

Table S35. Cartesian coordinates of molecule (**5b**) according to B3LYP/6-31G(d) level theory in gas phase.

	X	Y	Z
C	-1.74891500	-0.54133200	0.00004200
H	-1.74667200	-1.62341300	0.00006000
C	-0.67479900	0.26011500	0.00006200
H	-0.83450500	1.33499200	0.00003900
C	0.67483400	-0.26009600	0.00011400
H	0.83454100	-1.33497400	0.00014000
C	1.74894900	0.54135000	0.00012600
H	1.74673000	1.62343100	0.00009900
N	3.09592200	-0.01454200	0.00019300
O	3.23207000	-1.23800800	-0.00014300
O	4.01110700	0.80952300	-0.00018200
N	-3.09591600	0.01453300	-0.00001100
O	-3.23214300	1.23799200	-0.00007400
O	-4.01110200	-0.80953200	-0.00006100

Table S36. Cartesian coordinates of molecule (**5c**) according to B3LYP/6-31G(d) level theory in gas phase.

	X	Y	Z
C	0.29898100	1.84963800	-0.00028900
H	-0.70197400	2.26644200	-0.00044600
H	1.13193800	2.54343100	-0.00028400
C	0.49903800	0.53219700	-0.00012000
C	-0.49903800	-0.53219600	-0.00008300
C	-0.29898000	-1.84963700	-0.00016300
H	0.70197400	-2.26644100	-0.00029800
H	-1.13193700	-2.54343000	-0.00011000
N	1.89964500	0.02727000	0.00006200
O	2.41540400	-0.17012300	-1.09410200
O	2.41535600	-0.16948300	1.09436300
N	-1.89964400	-0.02726900	0.00007100
O	-2.41543100	0.16999000	-1.09410500
O	-2.41533000	0.16961300	1.09436100

Table S37. Cartesian coordinates of molecule (**5d**) according to B3LYP/6-31G(d) level theory in gas phase.

	X	Y	Z
C	-3.46368700	-0.59452200	-0.00001300
H	-3.44415900	-1.68185100	-0.00002100
H	-4.44190400	-0.12356900	-0.00002200
C	-2.33411300	0.13953300	0.00000800
H	-2.36772000	1.21939600	0.00001800
C	-1.05652800	-0.53322000	0.00001800
H	-1.09946600	-1.61860000	0.00000100
C	0.21684500	-0.06900000	0.00004200
N	1.30432100	-1.09350900	0.00004000
O	0.93006600	-2.26929300	-0.00008400
O	2.47183700	-0.74135900	0.00006300
N	0.57163700	1.35926300	0.00006400
O	-0.38716300	2.13870300	-0.00000300
O	1.74106600	1.70789800	-0.00010600

Table S38. Cartesian coordinates of molecule (**6b**) according to B3LYP/6-31G(d) level theory in gas phase.

	X	Y	Z
C	-2.61761600	0.37660300	-0.00000900
H	-2.50052800	1.46652800	-0.00004000
C	-1.51928300	-0.39916600	0.00001400
H	-1.64552000	-1.47943900	0.00004700
C	-0.18446700	0.15440700	-0.00000600
H	-0.06762300	1.23565600	-0.00005000
C	0.93224600	-0.59079200	0.00000200
H	0.98386900	-1.67115600	0.00003600
N	2.24276900	0.02663900	-0.00002000
O	2.32818500	1.25791300	0.00002200
O	3.20424100	-0.74782900	-0.00000700
N	-3.92797400	-0.19302700	0.00000600
H	-4.44923500	0.14311600	0.81140400
H	-4.44921500	0.14302500	-0.81144100

Table S39. Cartesian coordinates of molecule (**6c**) according to B3LYP/6-31G(d) level theory in gas phase.

	X	Y	Z
C	-0.05681200	1.90984900	-0.00000800
H	-1.02332100	2.39816200	-0.00000700
C	-0.00905800	0.57727400	-0.00000300
C	-1.15621400	-0.34578500	0.00000500
C	-1.01954600	-1.67956100	0.00001800
H	-0.05184100	-2.16997400	0.00002200
H	0.84412700	2.51335400	-0.00001200
H	-1.89309300	-2.32449400	0.00002300
N	1.32716400	-0.07016000	-0.00000300
O	1.82690400	-0.32307200	-1.09270100
O	1.82692700	-0.32302500	1.09269500
N	-2.42928200	0.33447200	-0.00000400
H	-2.97095600	0.06543200	-0.81839900
H	-2.97095900	0.06544700	0.81839300

Table S40. Cartesian coordinates of molecule (**6d**) according to B3LYP/6-31G(d) level theory in gas phase.

	X	Y	Z
C	0.23726900	0.35450800	-0.00005100
C	-0.88051100	-0.38102600	0.00005000
C	-2.22255100	0.17804900	0.00000400
C	-3.32893100	-0.57923900	0.00009100
H	-3.28069200	-1.66590400	0.00020100
H	-4.32005700	-0.13624300	0.00005500
N	0.32278300	1.76001100	-0.00021700
H	0.81542500	2.10243200	0.82442400
H	0.81540200	2.10224100	-0.82495100
N	1.55580700	-0.35748000	0.00003400
O	2.06644600	-0.59104400	-1.09354000
O	2.06663400	-0.59041500	1.09365400
H	-0.78371600	-1.46475200	0.00017300
H	-2.29278600	1.26242400	-0.00010500
